# The Use of Residual Blood Specimens in Seroprevalence Studies for Vaccine-Preventable Diseases: A Scoping Review

**DOI:** 10.3390/vaccines13030321

**Published:** 2025-03-18

**Authors:** Monica Pilewskie, Christine Prosperi, Abigail Bernasconi, Ignacio Esteban, Lori Niehaus, Connor Ross, Andrea C. Carcelen, William J. Moss, Amy K. Winter

**Affiliations:** 1 International Vaccine Access Center, Department of International Health, Johns Hopkins Bloomberg School of Public Health, Baltimore, MD 21205, USA; 2 Gavi, the Vaccine Alliance, 1218 Geneva, Switzerland; 3 Department of Epidemiology and Biostatistics, College of Public Health, University of Georgia, Athens, GA 30602, USA; 4 Department of Epidemiology, Johns Hopkins Bloomberg School of Public Health, Baltimore, MD 21205, USA; 5 Center for the Ecology of Infectious Diseases, University of Georgia, Athens, GA 30602, USA

**Keywords:** vaccine-preventable diseases, serology, seroprevalence, blood, residual

## Abstract

*Background*: Residual blood specimens offer a cost- and time-efficient alternative for conducting serological surveys. However, their use is often criticized due to potential issues with the representativeness of the target population and/or limited availability of associated metadata. We conducted a scoping review to examine where, when, how, and why residual blood specimens have been used in serological surveys for vaccine-preventable diseases (VPDs) and how potential selection biases are addressed. *Methods*: The review followed the Preferred Reporting Items for Systematic Reviews and Meta-Analyses extension for Scoping Reviews (PRISMA-ScR) guidelines and identified relevant papers published in 1990–2022. *Results*: A total of 601 articles met the inclusion criteria after title, abstract screening, and full-text review. The most studied VPDs using residual blood specimens were COVID-19 (27%), hepatitis E (16%), hepatitis B (10%), influenza (9%), HPV (7%), and measles (7%). Residual blood specimens were primarily sourced from diagnostic specimens (61%) or blood and plasma donations (37%). Almost all articles used specimens linked to basic demographic data (e.g., age and sex), with 47% having access to extended demographic data (e.g., geographic location). Common strategies to address potential biases included comparing results with published estimates (78%) and performing stratified analyses (71%). *Conclusions*: Residual blood specimens are widely used in seroprevalence studies, particularly during emerging disease outbreaks when rapid estimates are critical. However, this review highlighted inconsistencies in how researchers analyze and report the use of residual specimens. We propose a set of recommendations to improve the analysis, reporting, and ethical considerations of serological surveys using residual specimens.

## 1. Introduction

Immunoglobulin G (IgG) antibodies can be used to identify persons with prior exposure to vaccine-preventable diseases (VPDs) through infection or vaccination. Serological surveys (serosurveys) link serological testing of IgG antibodies results with individual demographic and epidemiologic data. These rich data sets can be used to directly estimate population seroprevalence profiles by characteristics of interest (e.g., age or space) and identify immunity gaps. Serosurveys can also be used to estimate disease burden [1,2], infer key epidemiological parameters (e.g., basic reproduction number or rate of infection) [1,3,4], evaluate surveillance and vaccination program performance [5,6], and estimate outbreak risk [7,8]. The results generated from high-quality serosurveys of VPDs can inform vaccination strategies and control the spread of disease [9].

High-quality serosurveys are population-based household surveys which use probability-based sampling designs to accurately include a representative sample of the population of interest. They require standardized laboratory methods with excellent quality assurance and control for the data to be appropriately analyzed and interpreted. The main limitations to conducting high-quality serosurveys include the substantial staff and resource requirements; the necessary sampling, laboratory, and analytic capacity; and the long timeframe needed to plan, conduct, test, analyze, and disseminate the results [10,11]. Consequently, routinely collected vaccination coverage and case surveillance data are relied upon to inform vaccination programs. While immunity profiles can be indirectly estimated for vaccine-preventable diseases given historical vaccination coverage and surveillance data, the quality of these data are highly variable [12], and serosurveys provide a less biased and more direct estimation of immunity profiles [7,13].

One way to increase the feasibility of serosurveys is to use residual blood specimens. Residual blood consists of remnant specimens collected for another purpose (for example, blood donations, past serosurveys, or other laboratory tests) that are available for an objective not originally intended. Using residual specimens in serosurveys reduces the resource commitment, including time and staff, for specimen collection, which is often the highest cost of a serosurvey [11]. Residual specimens also protect data collectors from exposure to emergent pathogens, a concern during the COVID-19 pandemic [14,15]. However, a critical limitation of using residual specimens is the potential for a biased sample that is not representative of the population of interest and conclusions that lack external validity. Additional limitations include ethical or legal constraints for the local context and resources constraints to maintain residual specimens at a research institute or health facility or to transport specimens to a central laboratory for testing [16,17]. The quality of seroprevalence studies utilizing residual specimens is directly related to the quality and availability of the linked metadata available for the specimens to allow investigators to assess and potentially adjust for biases.

In this scoping review, we evaluated the use of residual specimens in seroprevalence studies of VPDs. Specifically, we aimed to (1) characterize serological studies that used residual specimens; (2) describe the objectives of serological studies that use residual specimens; and (3) evaluate if and how authors handled or discussed potential selection bias. This review follows the authors’ observations that since the emergence of COVID-19, there has been an increase in published papers on serosurveys that use residual specimens. Additionally, with the development of multiplex technology for testing serum for antibodies to multiple antigens and integrated serosurveillance systems [18], there is potential for residual specimens to be more widely used to understand a broad array of pathogen exposures to guide interventions to better control the spread of infectious diseases. We sought to systematically evaluate our observation of an increase in serosurveys using residual specimens and understand the past and potential future role of residual specimens in the field of seroepidemiology to inform surveillance and response to outbreaks of VPDs.

## 2. Materials and Methods

We summarize the protocol below, but see Text S1 for the full protocol. This review is reported according to the Preferred Reporting Items for Systematic Reviews and Meta- Analyses extension for Scoping Reviews (PRISMA-ScR) statement (Text S2).

### 2.1. Inclusion and Exclusion Criteria

The inclusion criteria were studies that used residual human blood for serological testing of pathogens causing VPDs of interest: *Vibrio cholerae* (cholera), dengue viruses, *Corynebacterium diphtheriae* (diphtheria), hepatitis A virus, hepatitis B virus, hepatitis E virus, *Haemophilus influenza* type b (Hib), human papillomavirus virus (HPV), influenza virus, Japanese encephalitis virus, measles virus, *Neisseria meningitidis* (meningococcal meningitis), mumps virus, *Bordetella pertussis* (pertussis), *Streptococcus pneumoniae* (pneumococcus), poliovirus (poliomyelitis), rotavirus, rubella virus, SARS-CoV-2 virus (COVID-19), *Clostridium tetani* (tetanus), tick-borne encephalitis virus, *Mycobacterium tuberculosis* (tuberculosis), *Salmonella typhi* (typhoid fever), varicella-zoster virus (herpes zoster/shingles and varicella/chickenpox), and yellow fever virus. We defined serosurveys using residual blood as studies that tested remnant, surplus, existing, archived, or left-over blood samples for purposes beyond the original reason for sample collection. The serological testing must have been conducted to measure exposure or immune status and not acute infection; for example, testing conducted with IgG immunoassays, neutralization, or hemagglutinin-inhibition tests and not nucleic acid detection assays or IgM immunoassays. The objective of this scoping review was to evaluate the use of residual blood in serological surveys measuring population-level metrics; therefore, we excluded studies that used residual blood to evaluate diagnostic tests (e.g., [19]) or inform diagnostic test laboratory protocols or that used residual blood as a comparison group (e.g., [20,21]).

### 2.2. Information Sources and Literature Search

The screening of articles was conducted using the English, French, Spanish, Italian, Russian, Chinese, and Portuguese language literature, including the peer-reviewed literature, grey literature, and pre-prints, between January 1990 and August 2022. We excluded editorials, letters, commentaries, narrative reviews, and conference abstracts. Initial screening was conducted in June 2021 and re-run in August 2022 to include studies published after the initial screening. Using search terms for vaccine-preventable diseases, serologic testing, and residual samples, the following electronic databases were used: PubMed, Scopus, Embase, Cochrane, and the World Health Organization (WHO) Institutional Repository for Information Sharing (IRIS) database. Additional studies were identified through suggestions from experts at the United States Centers for Disease Control and Prevention (CDC) and WHO. We worked with librarians at Johns Hopkins University to create a list of search terms based on our inclusion and exclusion criteria. Database-specific search terms can be found in the protocol (see Text S1).

### 2.3. Screening Process

The screening criteria were established per the protocol and standardized among the investigators. After deleting duplicates, two members of the literature review group systematically screened the title and abstract of papers for the inclusion and exclusion criteria using the Covidence systematic review software [22]. Those that met the criteria underwent full-text review. The title and abstract and full-text screenings were conducted by two reviewers with disagreements resolved through discussion and arbitration with a third reviewer.

### 2.4. Data Extraction Process and Data Points

A data extraction form was developed a priori utilizing the KoboToolbox data collection platform and later calibrated following full-text review (see Text S3 for data extraction survey). Data were extracted by one reviewer. A second reviewer was consulted if questions arose about adherence to inclusion and exclusion criteria or the data extraction process. The main data points collected included the following: VPD of interest, specimen countries of origin, year of original sample collection, the objectives of the serological study based on the abstract or summary alone (see Table S1 for more detail), original population from which the specimens were collected (see Table S2 for more detail), metadata linked to residual samples (e.g., age, sex), permission and ethical considerations around testing residual samples, the investigator’s approach to exploring, discussing, or addressing potential selection biases (see Table S3 for more detail), and if collection was part of a larger serological surveillance system (see Text S4). No attempts were made to contact study investigators to obtain or confirm data or critically appraise the source of data.

### 2.5. Data Synthesis

Data synthesis was conducted using descriptive statistics. Data cleaning was conducted with SAS Version 9.4. All figures were created with the R statistical computing software (version 4.3.1; R Core Team 2023) using packages *ggplot2* (version 1.1.2), *sf* (version 1.0.14), *rnaturalearthdata* for the global shapefile (version 0.1.0), and *unikn* (version 0.8.0) to define color palettes.

## 3. Results

**Figure 1:**
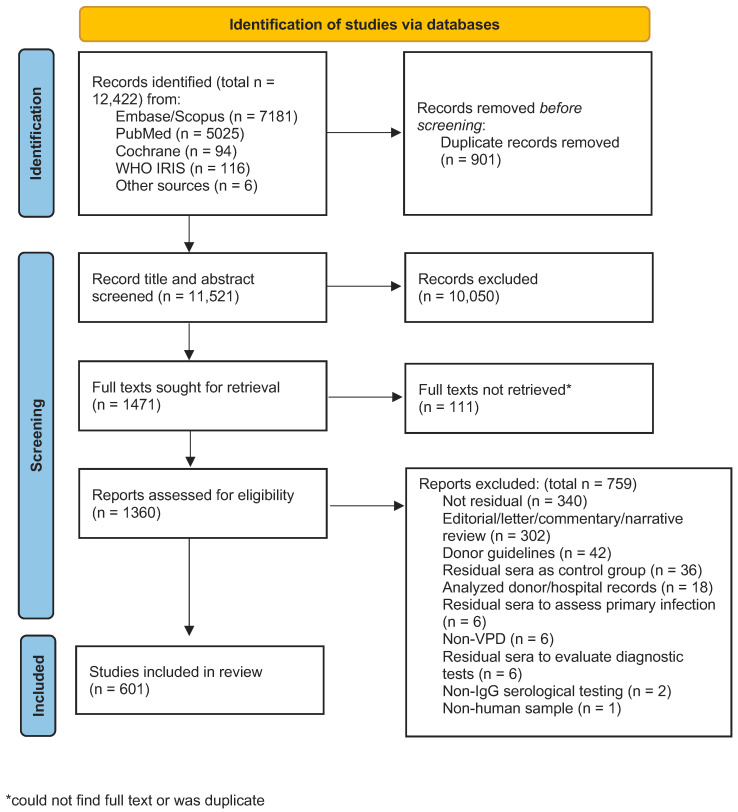
Flow diagram of seroprevalence studies in the scoping review, from [23].

The initial and secondary database searches yielded 11,521 unique articles eligible for screening (Figure 1). Of the 11,521 articles, 10,050 (87%) were excluded via title and abstract review. Among the 1360 articles in the full-text review, 340 (25%) were excluded because the study did not use residual specimens, 302 (22%) were editorials, commentaries, or narrative reviews, and 117 articles (8%) were excluded based on other remaining criteria. Data extraction was conducted on 601 unique articles to be included in the final analysis (see Table S4 for a list and bibliography of all 601 articles).

### 3.1. VPDs Studied, Trends, and Objectives

**Figure 2:**
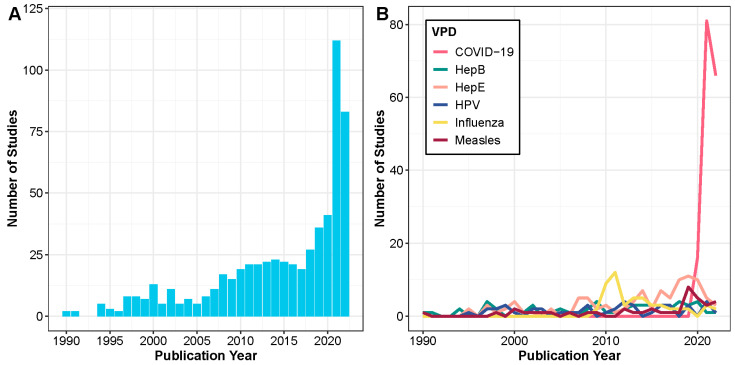
Time series of studies’ publication year (**A**) across all VPDs (N = 601 studies) and (**B**) by the six most studied VPDs.

**Figure 3:**
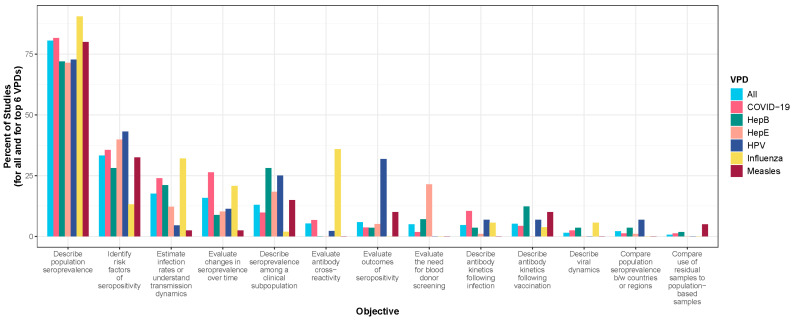
Bar plot displaying percent of studies by objective for all VPDs and for the six most studied VPDs (N = 601 studies). Note the categories are not mutually exclusive meaning that one study could have conducted testing for multiple pathogens and have had multiple objectives. Refer to Table S2 for additional information.

Over 75% of the articles studied one or more of the following six VPDs: COVID-19, hepatitis E, hepatitis B, influenza, HPV, and measles. COVID-19 was the most common vaccine-preventable disease studied between 1990 and 2022 (27.1% of articles), even though articles on COVID-19 were only published beginning in 2020 (Table 1). Seroprevalence studies of hepatitis E, hepatitis B, influenza, HPV, and measles were the next most common VPDs using residual specimens (Table 1). Overtime, an increase was observed in the number of published serological studies that use residual specimens (Figure 2A). The increase in serological articles using residual blood after 2020 was due to serological studies on COVID-19 (Figure 2B). There was an increase in articles studying measles from 2019 to 2020 during the global resurgence, hepatitis E from 2018 to 2019 following outbreaks in Bangladesh and South Sudan, and influenza from 2010 to 2011 following the 2009 H1N1 influenza virus pandemic (Figure 2B). The objective of serological surveys that used residual specimens was most often to describe population seroprevalence (80.5%), followed by the desire to identify risk factors for seropositivity (33.3%), estimate infection rates (17.6%), and evaluate trends in seropositivity (15.8%) (Figure 3, Text S5, Table S5). Studies on emergent pathogens such as COVID-19 and influenza disproportionally focused on the latter two objectives.

### 3.2. Original Use of Specimens

**Figure 4:**
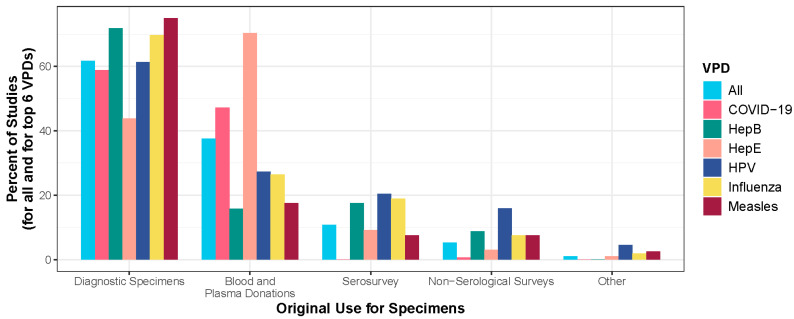
Bar plot displaying the percent of studies by the original use of the specimens for all VPDs and for the six most studied VPDs (N = 601 studies). Note the categories are not mutually exclusive, meaning that one study could have conducted testing for multiple pathogens or have used specimens from multiple original sources.

The original use of residual specimens for most articles was as clinical diagnostic specimens (Figure 4). However, the most common original use for hepatitis E articles was for blood and plasma donations. Sixteen percent of specimens were originally collected for research purposes (serological survey or non-serological survey). This included surveys for specific medical conditions (e.g., hypertension, heart disease, and lead exposure) or VPDs besides what the residual specimens were subsequently tested for.

### 3.3. Sources of Residual Specimens

**Figure 5:**
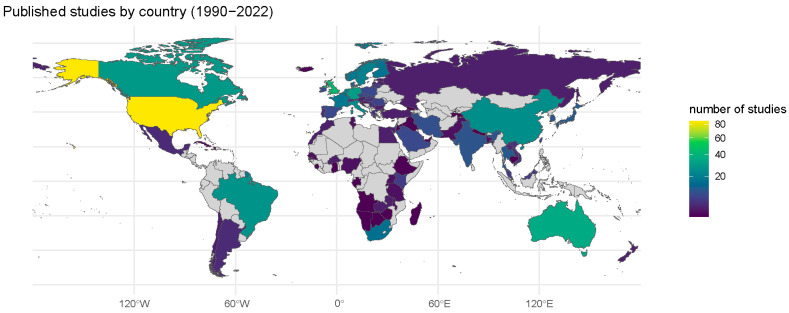
Map of published studies by country of original source population (N = 601 studies).

The original population from which specimens were collected, and the collection site, was highly related to the original purpose for specimen collection. For example, given that most of the residual specimens’ original use was for diagnostic specimens, the main sources of residual specimens were patient populations from hospitals, clinics, or diagnostic centers (see Text S5, Figures S1 and S2, and Table S6). The mean time between collection of specimens and publication of the study was 6.5 years, but the time between collection and publication was faster for COVID-19 at 1.7 years (Text S5, Figure S3). In terms of location of source, a higher proportion of studies were conducted in the United States than in any other country, making up 14% (85 of 601) of articles published between 1990 and 2022 (Figure 5). Studies using specimens collected in low- and middle-income countries made up 35% (210 of 601) of articles. Moreover, 28 (4.7%) of the 601 articles reported that the testing was part of a larger serological surveillance system (Text S4, Table S7), including the Health Protection Agency National Seroepidemiology Program from the UK (12 articles) European Seroepidemiology Network (ESEN) (11 articles), Australia’s National Centre for Immunisation Research and Surveillance (4 articles), and Vietnam’s serosurveillance (1 article).

### 3.4. Metadata Linked to Residual Specimens

Almost all articles used specimens linked to basic demographic data (e.g., age and/or sex), with 47% having access to extended demographic data such as geographic location (Table S8). When specimens from a previous serosurvey were used (11% of articles), 55% and 42% of these articles had access to extended demographic and epidemiologic metadata (e.g., vaccination status), respectively. However, most articles (92%) used diagnostic specimens or specimens from blood or plasma donations, and fewer than half (46%) had extended metadata, and only 11% had epidemiologic data.

### 3.5. Ethical Considerations in the Use of Residual Specimens

Just over two-thirds (70.4%) of the articles reported receiving approval by an ethics board or committee to access and test the residual specimens (Table S9), with 35% of those studies using specimens from the United States, Australia, Canada, or the United Kingdom. One-third (33.1%) of articles mentioned that broad individual consent for additional testing was obtained at the time of specimen collection, with most collected for blood or plasma donation or being clinical specimens; only 21.6% were from a survey. One-third (33.1%) mentioned the residual specimens were deidentified; among these, all reported at least one additional ethical step was taken (i.e., 72% indicating a committee approved additional testing, 27% indicated broad individual consent for additional testing, 14% received waiver for reconsenting, and 4% were exempt as public health surveillance) (Table S10). Studies that used specimens collected for clinical purposes were least likely to report that broad individual consent for additional testing was obtained but were most likely to report that specimens were deidentified. Fifteen percent of articles did not include any statement on ethics or permissions.

### 3.6. Selection Bias

**Figure 6:**
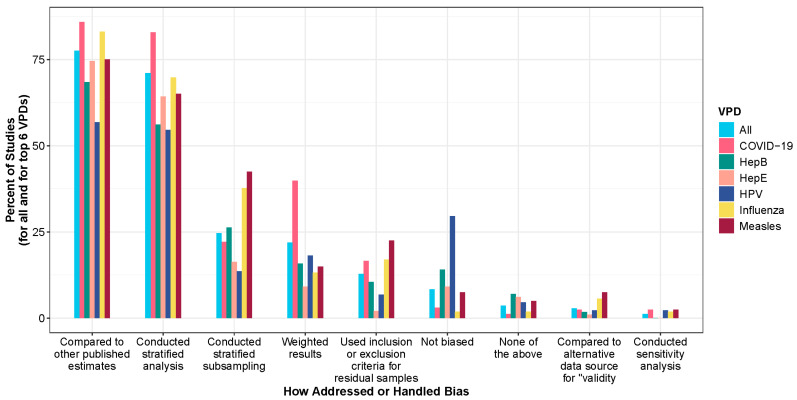
Exploring or addressing selection bias for all VPDs and for the six most studied VPDs (N = 601 studies). Note the categories are not mutually exclusive, meaning that one study could have conducted testing for multiple pathogens or have discussed or handled bias in multiple ways. Refer to Table S3 for additional information.

A variety of approaches were used to explore or address potential selection bias due to the use of residual specimens (Table S3). Figure 6 shows the percentage of studies that use different approaches to address or handle bias overall and by the top six VPDs. The most common approach was to compare results to other published estimates (77.5%) (Figure 6). Conducting stratified analyses to control for differences in the sample and target populations (e.g., by age, sex, or location) was the second most common method (71%) across all VPD groups. About one quarter of studies used design-related considerations, such as stratified subsampling, to better reflect the population of interest (24.6%); this approach was particularly prevalent for influenza and measles studies. For example, Carcelen et al., 2022 sub-sampled HIV serosurvey specimens in Zambia by age, province, and HIV infection status to obtain a representative sample and estimate measles seroprevalence by age and province [8]. Another frequently used approach included weighting results using characteristics linked to residual specimens (e.g., age, sex, or location) to align with the distribution of characteristics in the target population (22%). For example, Ho et al., 2020 estimated seroprevalence to hepatitis E virus in Belgium through weighting by province and age to account for differences in sampling [24]. Some studies (12.8%) relied on inclusion and exclusion criteria to reduce selection bias from the use of residual specimens (e.g., exclusion of specimens from a specific medical ward at a facility, specimens from patients admitted for respiratory illness, or patients who were immunocompromised [25]). Sensitivity analyses that varied the input data, model assumptions, or model parameters were used in only 1% of the studies. For example, a study that examined associations between esophageal squamous cell carcinoma and HPV serological markers in serum from six existing case–control studies conducted sensitivity analysis by excluding two studies in which blood collection procedures differed for the case and control subjects [26]. Lastly, 8.3% of studies used a non-biased sample of specimens originating from the population of interest, particularly among hepatitis B, hepatitis E, and HPV studies. For example, a hepatitis E serosurvey used residual blood donor specimens to evaluate the need to include hepatitis E in blood donor screening [27], and an HPV serosurvey used residual diagnostic specimens from patients with confirmed squamous-cell carcinoma of the oropharynx to assess the risk of oropharyngeal cancer as an outcome of HPV seropositivity [28].

## 4. Discussion

We sought to characterize serological studies of VPDs that use residual blood specimens. The benefits of high-quality population-based serological surveys to understand and inform the control of infectious diseases are well documented [9,29]. However, high quality population-based serosurveys require resources of time, money, technical expertise, and cooperation of the population [11,30,31]. Residual specimens can be leveraged to reduce the time and cost of serosurveys, but the potential lack of representativeness of the population of interest and limited metadata are limitations.

Serosurveys utilizing residual specimens are advisable for various purposes, particularly for providing rapid insights during outbreaks of emergent or re-emergent infectious diseases. We found sharp increases in the number of serosurveys using residual specimens during the 2009–2010 H1N1 influenza outbreak, the 2019–2020 measles outbreaks, and the 2019–2021 COVID-19 pandemic. While most of these studies aimed to determine population seroprevalence (e.g., [32]), a smaller fraction focused on estimating parameters related to the magnitude or timing of infection, such as incidence, prevalence, and force of infection. For example, Bassal et al., 2021 used stored samples from 2015 to assess measles seropositivity among an Israeli population to understand age-specific incidence rates of the 2018–2019 measles outbreak [33]. Similarly, Routledge et al., 2022 tested for COVID-19 seroprevalence using residual specimens from blood draws to estimate the probability of prior infection among geographic regions in San Francisco, USA [34]. Residual specimens may be particularly critical when new data collection may be risky or difficult to conduct. For instance, Uyoga et al., 2021 used residual specimens from Kenyan blood donations in April–June 2020 to estimate COVID-19 seroprevalence and burden of disease during a time of movement restrictions in the country [14]. Residual specimens are also advisable to evaluate trends over time. Assuming the type and magnitude of selection bias stays constant across the period of interest, using residual specimens for this purpose can also overcome potential selection bias to obtain useful information. Both influenza and COVID-19 had disproportionally more studies assessing trends over time than all studies using residual specimens (e.g., [35]), highlighting the role of serosurveys that use residual specimens during emergent events.

The use of residual specimens is also advantageous when they accurately represent the target population. For example, many articles on hepatitis E serology used residual specimens from blood donors to evaluate the need for blood donor screening (e.g., [36,37,38]). Hepatitis B and HPV serosurveys have used residual specimens from patients exhibiting potential sequelae of these infections to investigate the outcomes of seropositivity [28,39]. In such cases, the common concern regarding the representativeness of residual specimens is mitigated. However, it is important to note that the primary objective of most studies using residual specimens is to describe population-level seroprevalence or to identify risk factors for seropositivity, relying on samples that may not represent the population of interest. For example, we saw an increase in the number of hepatitis E serological surveys using residual specimens in 2016–2019, mostly from blood donor specimens (e.g., [40]), that we attribute to a rising interest in estimating population immunity due to the availability of the Hecolin hepatitis E vaccine that was licensed in China in 2011 and the need to understand the incidence of hepatitis E virus infection [41]. In these instances, approaches to assess or account for selection bias are needed.

Studies have adopted various approaches to assess or mitigate selection bias (Table S3). Understanding these approaches is crucial for interpreting the results and determining the generalizability of findings from residual specimens [42]. Over 70% of the studies utilized straightforward methods such as comparing their results with other published estimates or conducting stratified analyses. Both approaches can be applied post-testing of residual specimens; the latter only requires basic demographic metadata like age and sex, which were accessible in almost all studies. While comparison can enhance the external validity of findings, it is not always a reliable method. Stratifying results helps control for differences between the sample and target populations by ensuring that each homogeneous subgroup is represented in the analysis, thus providing a more nuanced understanding of seroprevalence across key demographic characteristics. However, without adjusting the weight of each subgroup to match their actual proportions in the overall population, the analysis might not accurately reflect these proportions, potentially skewing results.

For more rigorous bias mitigation, design-phase methods like stratified subsampling and the use of specific inclusion and exclusion criteria, as well as analytic methods such as post hoc weighting, are preferred. These approaches, which require detailed metadata linked to characteristics known to influence seropositivity, are ideal but more complex to implement. They also necessitate an understanding of the population distributions of the relevant characteristics. For instance, Murhekar et al. (2021) utilized stored samples from a dengue serosurvey with comprehensive demographic data to estimate diphtheria seroprevalence across different regions and rural or urban areas of India [43].

The extent of adjustment needed depends on the initial bias of the residual population. We identified three studies that directly compared seroprevalence estimates from residual clinical specimens with those from a population-based serosurvey to address the utility of residual specimens; two estimated COVID-19 seroprevalence in the US [44,45] and one estimated seroprevalence to VPDs in Australia [46]. All three employed intentional design-phase and/or analytic-phase strategies to mitigate selection bias, finding that seroprevalence estimates from residual specimens were comparable to those from population-based surveys. This supports the credibility of using residual specimens in certain contexts, provided that selection bias is adequately addressed.

The general recommendation is to consider the added risks for bias when using residual specimens. If possible, address these risks during the study design and implementation phases. If that is insufficient, strategies should also be devised for adjusting during the analysis phase. For example, Choisy et al. (2019) analyzed residual samples stored at a Vietnamese national biobank for clinical purposes to estimate measles seroprevalence. They tackled potential selection bias by subsampling according to age, sex, and location, stratifying the results by these demographics and comparing their estimates to known measles cases and vaccination coverage [47].

Beyond helping account for selection bias, metadata accompanying residual specimens—such as extended demographic data (e.g., residence, socioeconomic status, and occupation) and epidemiologic data (e.g., vaccination status or infection history)—are vital for interpreting serological markers. For example, Yan et al. (2019) assessed hepatitis A seroprevalence using stored samples from a hepatitis B serosurvey that included demographic and epidemiologic metadata on hepatitis A vaccination. This allowed them to examine changes in seroprevalence pre- and post-vaccine introduction and to make comparisons between vaccinated and unvaccinated groups [48]. Epidemiologic metadata are especially valuable if concerns about cross-reactivity in antibody testing exist. Establishing a standard set of basic metadata linked and reported with residual specimens could prove useful, acknowledging ethical considerations and the necessity for informed consent when collecting or abstracting additional data. The COVID-19 Immunity Task Force in Canada recommended a core set of metadata, including demographics and a history of vaccination or infection, to standardize analyses across residual specimens collected during the early years of the COVID-19 pandemic [49].

Two-thirds of the articles used specimens from high-income countries, despite the larger burden of VPDs in low- and middle-income countries. Scaling up serosurveys using residual specimens may prove to be of greater value in resource-limited settings, given that using residual specimens require less time, cost, and resource capacity compared to population-based serosurveys. For example, Kelly et al., 2002 found that a serosurvey using residual clinical specimens was 11 times less costly than a population-based school serosurvey [46]. However, there remain important cost considerations after specimen collection regardless of the savings gained by using residual specimens. For example, health facilities may not have the capacity to store residual specimens collected for routine testing purposes. To use these specimens for additional testing, specimens need to be transported to another laboratory (e.g., research institute or district-level health facility) for storage until testing, along with linked data. This requires staff to process specimens and extract data as well as transportation to move specimens between facilities. Similarly, specimen repositories, such as those from past serosurveys, require sustained infrastructure to maintain frozen specimens.

In addition to the logistical considerations, there are ethical considerations to using specimens for a different purpose than originally intended. Much of the bioethics work related to residual specimens is focused on genomics or molecular disease surveillance, which have specialized considerations related to the types of data available and purposes (e.g., “readily identifiable” genetic information and tracking transmission networks) [50,51]. For serological surveys using residual specimens to develop population-level estimates of VPDs, information is typically presented as summary-level measures and ideally used by a public health agency to inform vaccination programs. Although this is generally accepted as benefiting the community, there are still important ethical considerations that are relevant for any research using residual specimens. These include understanding the population whose specimens were collected (e.g., whether they include vulnerable populations), the original purpose of the specimens, whether the individuals whose specimens were collected were aware of the potential for future research, if there were any formal consent or “opt-in/opt-out” requirements related to future research, and the policy and regulatory environment in the setting where the specimens were collected. Information about future research on residual specimens is typically provided during the consent process for research studies. We found many inconsistencies in how articles discussed permission to use residual specimens and ethical considerations. Many articles reported that ‘broad consent’ was obtained at the time of specimen collection, although not all countries or settings allow this [52]. We identified only two articles that described reconsenting individuals and both involved contacting individuals or their caregivers to obtain consent for the additional testing [53,54]. How information about future research is communicated to blood or plasma donors or patients varies by setting. Studies using clinical specimens often described the specimens as being de-identified or anonymized; however, these terms may be used or interpreted differently, and policies and procedures related to these vary by country [55]. For example, in the United States, there are 18 specified identifiers that must be removed for the data to be considered “de-identified” [56]. Few of these identifiers are relevant for seroprevalence studies, but data on age and geographic location are usually important, so special considerations to access these data may be needed depending on the research questions.

Our analysis identified articles that used residual specimens collected as part of serological surveillance systems. For example, the European Sero-Epidemiology Network (ESEN) is a network of European countries that aim to standardize serological surveillance for comparison across space and time. Some of the countries rely on residual specimens, while others use population-based serosurveys [57]. This supports the idea that serological surveillance systems can leverage residual specimens (e.g., diagnostic specimens). In turn, specimens from serosurveillance systems can be stored in biorepositories for future use. Serological surveillance systems can also be integrated, relying on multiplex technology to test for IgG antibodies to many antigens and pathogens simultaneously. The expansion of integrated serosurveillance to more low- and middle-income settings is where there is highest value [18,58]. Integrated serological surveillance systems, particularly those that use multiplex assay technologies to simultaneously test for multiple pathogens, have the potential to improve the scale-up and utility of serological surveys, thus requiring less specimen volume [59]. Integrated serosurveillance can prospectively collect new specimens, as in the Netherlands [60], or take advantage of residual specimens, including from population-based serosurveys for HIV [61] or malaria [62].

The design of a serosurvey using residual specimens varies by the VPD, target population, and research question of interest. Given the specified challenges in screening for articles that report the use of residual specimens and inconsistencies in how authors discussed the use of residual specimens, we recommend a standard set of descriptors for serological studies using residual specimens (Table 2). These recommendations are motivated by the following: (1) the need to detail the original specimen source to understand the potential for selection bias; (2) the ethics such that it is clear whether permissions were obtained and ethics of using residual specimens was considered; (3) documentation of broad learnings from serological surveys using residual specimens so that we continue to evaluate the limitations and potential strengths of these studies for the control of VPDs; and (4) the ability to use seroprevalence estimates for further analyses including comparisons across settings or meta-analyses.

There were several limitations in conducting this scoping review. Despite using a broad search strategy to capture the varied terms used to describe residual specimens, there may have been articles that published results from VPD serosurveys that used residual specimens missed by our search. For example, for many articles, particularly those using specimens from patients and blood donors, it was difficult to ascertain if the specimens were left over from clinical blood collection or diagnostic testing, or if they were purposefully collected from convenience populations for a serosurvey and, therefore, were not residual specimens. Additionally, there was a class of articles that came from large national cross-sectional serosurveys in which it was difficult to determine the original purpose of the specimen collection to ascertain if the specimens were indeed residual (e.g., PIENTER or NHANES). There was a second class of articles from serosurveillance systems (e.g., ESEN) for which it was also challenging to determine if residual specimens were used. Ultimately, if there was no clear evidence the specimens were residual, then the article was excluded. This rule likely excluded articles that used residual specimens but did not explicitly describe them as such. Given that only 10% of all articles met the screening criteria, there were challenges in narrowing the search strategy and screening. Lastly, among the 25 VPDs, 10 VPDs were studied by fewer than 1% of the included articles (Hib, Japanese encephalitis, tick-borne encephalitis, yellow fever, meningococcal meningitis, pneumococcus, rotavirus, tuberculosis, typhoid fever, and cholera). This low number likely reflects the fact that serological tests are not well suited to study these pathogens [9,29], or that under-represented geographies in serological surveys are also where the burden of these pathogens are most prevalent.

## 5. Conclusions

Serological surveys that rely on residual specimens have been used for decades. Their value to estimate seroprevalence to inform disease burden estimates (e.g., COVID-19 [63]), assess time trends in seroprevalence, estimate infection rates, or assess risk factors for exposure (e.g., pandemic influenza H1N1 [64]) is especially noted during outbreaks of novel pathogens. During the COVID-19 pandemic when countries wanted rapid assessments of the prevalence of infection, vaccine effectiveness, waning immunity, and population susceptibility, residual specimens were a key source of serological data [65]. Serological surveys using residual specimens were also a valuable source to inform preventative strategies (e.g., measles [66]) or epidemiologically understand re-emergent outbreaks (e.g., measles [33]). The primary concern with residual specimens is whether they are representative of the target population and if relevant metadata are available to assess and adjust for biases in the design or analyses. By reporting key parameters about study design and results, we can better evaluate the potential biases of studies using residual specimens to alleviate concerns of external validity or extract valuable information despite these biases. Ultimately, having a serosurveillance system in place that leverages residual specimens provides a platform to test for emerging pathogens, supporting pandemic preparedness initiatives [67,68].

## Figures and Tables

**Table 1:** Number of articles reporting serological surveys using residual blood specimens by VPD. Note the categories below are not mutually exclusive, meaning that one article could have conducted testing for multiple pathogens.

Vaccine-Preventable Disease	Total No. (%) (N = 601)
COVID-19	163 (27.1)
Hepatitis E (HepE)	98 (16.3)
Hepatitis B (HepB)	57 (9.5)
Influenza	53 (8.8)
Human papillomavirus (HPV)	44 (7.3)
Measles	40 (6.7)
Hepatitis A (HepA)	39 (6.5)
Rubella	33 (5.5)
Dengue	31 (5.2)
Varicella/Herpes zoster	30 (5)
Diphtheria	19 (3.2)
Mumps	17 (2.8)
Pertussis	17 (2.8)
Poliomyelitis	8 (1.3)
Tetanus	7 (1.2)
Japanese encephalitis	4 (0.7)
Hemophilus influenza type b (Hib)	3 (0.5)
Yellow fever	3 (0.5)
Meningococcal meningitis	2 (0.3)
Tick-borne encephalitis	2 (0.3)
Rotavirus	1 (0.2)
Typhoid fever	1 (0.2)
Cholera	0 (0.0)
Tuberculosis	0 (0.0)
Pneumococcal	0 (0.0)
Studies investigating single pathogen	560 (93.2)
Studies investigating multiple pathogens	41 (6.8)

**Table 2:** Recommendations on reporting studies using residual specimens.

Component	Considerations
Study population	Details on the source of the specimens and description of the original population or study (including sample size and sociodemographic characteristics) to assess the generalizability of findings from residual specimens
	Original reason or objective for specimen collection prior to storage
	How residual specimens were sampled including inclusion and exclusion criteria (e.g., by time period and individual characteristics)
Ethics	Whether the individuals whose specimens were collected were aware of potential for future research; if there were any formal consent or “opt-in/opt-out” requirements related to future research; ethical approvals or waivers obtained, whether or not specimens were identifiable
Metadata	How data were collected from the original population (including if part of a larger surveillance collection system) and what metadata were obtained, including the following: Age (e.g., DOB and age category); Sex/gender; Geographic location (e.g., GPS, community, health facility catchment area, and administrative level); Vaccination history data Any other available sociodemographic data.
Serological testing	Serological testing that was performed for the original purpose prior to being stored
Selection bias and generalizability	How the study handled and assessed selection biases in the residual specimen source population relative to the target population. This explanation is key to providing readers with an understanding of how representative the findings may be of the target population. This could also include any potential limitations in the generalizability of the findings.

## Data Availability

The data presented in this study are openly available in Github at https://github.com/HopkinsIVAC/ScopingReview_SerosurveyResidual.

## Supplementary Materials

The following supporting information can be downloaded at: https://www.mdpi.com/article/10.3390/vaccines13030321/s1, Text S1: Protocol, Text S2: Preferred Reporting Items for Systematic reviews and Meta-Analyses extension for Scoping Reviews (PRISMA-ScR) Checklist, Text S3: Kobo Toolbox Data Extraction Survey, Text S4: Defining Serological Surveillance System, Text S5: Supplemental Results, Figure S1. Bar plot displaying percent of studies by original sample population for all VPDs and for the six most studied VPDs, Figure S2. Time series of studies by publication year based on (A) original source population specified in the studies and (B) original use for specimens specified in the studies, Figure S3. Histogram of difference between publication year and year of original sample collection, Table S1. Handling of paper objectives based on article’s abstract, Table S2. Examples of original sample populations, Table S3. Handling of selection bias in papers using residual specimens, Table S4. Studies selected for data extraction and analysis, Table S5. Paper objective excluding articles describing population seropositivity, Table S6. Collection site and Storage sites of the sample, Table S7. Number of articles utilizing residual specimens from serological surveillance systems, Table S8. Meta-data linked to residual samples by the original use of the sample, Table S9. Permissions and ethical considerations by original use of specimens, Table S10. Permissions and ethical considerations for articles mentioning residual specimens were de-identified.
