# Supplementary material for: The Use of Residual Blood Specimens in Seroprevalence Studies for Vaccine-Preventable Diseases: A Scoping Review
Source: Vaccines (Basel). 2025 Mar 18;13(3):321. doi: 10.3390/vaccines13030321 (PMC11945995; doi:10.3390/vaccines13030321)
Supplement: Supplementary file 1 [file vaccines-13-00321-s001.zip › vaccines-3492577-supplementary.pdf]

Supplemental Materials

The Use of Residual Blood Specimens in Seroprevalence Studies for Vaccine-Preventable Diseases: A Scoping Review

Monica Pilewskie[1], Christine Prosperi[1], Abigail Bernasconi[1], Ignacio Esteban[1,2], Lori Niehaus[1], Connor Ross[3], Andrea C Carcelen[1], William J Moss[1,4], Amy K Winter[3,5,*]

1 International Vaccine Access Center, Department of International Health, Johns Hopkins Bloomberg School of Public Health, Baltimore, MD 21205, USA

2 Gavi, the Vaccine Alliance, 1218 Geneva, Switzerland

3 Department of Epidemiology and Biostatistics, College of Public Health, University of Georgia, Athens, GA 30602, USA

4 Department of Epidemiology, Johns Hopkins Bloomberg School of Public Health, Baltimore, MD 21205, USA

5 Center for the Ecology of Infectious Diseases, University of Georgia, Athens, GA 30602, USA

* Correspondence: awinter@uga.edu

Text S1: Protocol

Review Question

What are the characteristics, objectives, and considerations of serological studies that use residual blood specimens to measure population-level metrics?

What are residual specimens within the scope of serological surveys?

They are residual, remnant, surplus, existing, archived, or left-over serum that are serologically tested for reasons beyond the objective of the original sample collection.

Examples of residual sera include:

- Sera originally collected for other serosurveys, studies, or surveillance systems and stored.
- Sera originally collected for clinical purposes at a health facility or laboratory and stored.
- Sera originally collected for donation purposes and stored.

Inclusion Criteria

- Uses any residual serum samples for serological testing of infectious disease to measure immune status.
    - Note *any* means at least some of the serum samples are residual.
    - Residual serum samples for serological testing are remnant, surplus, existing, archived, or left-over serum that are serologically tested for reasons beyond the objective of the original sample collection.
    - The following are a list of diagnostic tests that measure immune status (i.e., not acute infection):
        - neutralization tests (NTs) - plaque reduction neutralization tests
        - hemagglutinin-inhibition (HI)
        - IgG immunoassays - enzyme immunoassay (EIA), enzyme-linked immunosorbent assay (ELISA), florescent immunoassays (FIA), radio-immunoassays (RIA), chemiluminescence immunoassays
        - Multiplex
        - Rapid test IgG
- Serological testing related to the 25 pathogens of interest (i.e., vaccine preventable diseases except for rabies, anthrax, and smallpox). The following are the pathogens of interest:
    - *Vibrio cholerae* (cholera), dengue, *Corynebacterium diptheriae* (diphtheria), hepatitis A (HAV), hepatitis B (HBV), hepatitis E (HEV), *Haemophilus influenza* type b (Hib), human papillomavirus (HPV), influenza, Japanese encephalitis, measles, *Neisseria meningitidis* (meningococcal meningitis), mumps, *Bordetella pertussis* (pertussis), *Streptococcus pneumoniae* (pneumococcal), poliovirus (poliomyelitis), rotavirus, rubella, SARS-CoV-2 (COVID-19), *Clostridium tetani* (tetanus), tick-borne encephalitis, *Mycobacterium tuberculosis* (tuberculosis), *Salmonella* Typhi (typhoid fever), varicella-zoster (herpes zoster/shingles & varicella/chickenpox), yellow fever
- Peer-reviewed literature, grey literature, and pre-prints
- English, French, Spanish, Portuguese, Italian, Russian, Chinese language articles published between January 1, 1990 and August 22, 2022

Exclusion Criteria

- Serological testing of pathogens of non-interest: all non-VPDs (e.g., HIV, Hepatitis C, malaria, etc), and three VPDs (rabies, anthrax, smallpox)

- Use residual sera samples for serological testing of infectious disease to test for primary or acute infection only. The following are a list of diagnostic tests that measure acute infection (i.e., not immune status):
    - nucleic acid detection assays (e.g., PCR)
    - IgM immunoassays
    - Complement fixation test (CFT)
    - Point-of-care (POC) tests
- Use residual sera samples to test for Hepatitis B Surface Antigen (HBsAg) only
- Use residual sera samples to evaluate diagnostic tests or inform diagnostic tests laboratory protocols
- Use residual sera samples as a control (or comparison) group to a non-residual population of interest
- Use residual samples to modify existing blood donor screening guidelines/protocols; *existing* meaning we exclude studies that use residual specimens to determine *how to screen* for an already routinely screened pathogen (note: this differs from studies that are attempting to determine *whether to screen* for a new pathogen, which would be included).
- Biological samples are not blood sera. The following are a list of biological specimens that are not a specimen of interest:
    - placental/umbilical cord sera, cerebrospinal fluid sample, oral fluid sample, ocular specimens/fluid, tissue sample
- Animal studies and studies in other non-human specimens
- Editorials, letters, commentaries, narrative reviews, conference abstracts

Databases

- PubMed
- Scopus
- Embase
- Cochrane
- WHO IRIS

Search Terms

<u>PubMed (first search conducted 6/23/2021; second search conducted on 8/22/2022)</u>
("measles"[Mesh] OR "measles virus"[Mesh] OR "measles"[tiab] OR "sars-cov-2"[MeSH Terms] OR "sars-cov-2"[All Fields] OR "sars cov 2"[All Fields] OR "sars-cov-2"[tiab] OR "whooping cough"[Mesh] OR "pertussis"[tiab] OR "rubella"[Mesh] OR "rubella"[tiab] OR "papillomaviridae"[Mesh] OR "human papillomavirus"[tiab] OR "influenza, human"[Mesh] OR "cholera"[Mesh] OR "cholera"[tiab] OR "neisseria meningitidis"[Mesh] OR "meningococcal infections"[Mesh] OR "diphtheria"[Mesh] OR "diptheria"[tiab] OR "mumps"[Mesh] OR "mumps"[tiab] OR "tetanus"[Mesh] OR "tetanus toxoid"[Mesh] OR "tetanus"[tiab] OR "Hepatitis A"[Mesh] OR "Hepatitis B"[Mesh] OR "Hepatitis E"[Mesh] OR "hepatitis"[tiab] OR "Tuberculosis"[Mesh] OR "Pneumococcal Infections"[Mesh] OR "Typhoid Fever"[Mesh] OR "Poliomyelitis"[Mesh] OR "haemophilus influenzae type b"[mesh] OR "Herpes Zoster"[Mesh] OR "Herpesvirus 3, Human"[Mesh] OR "varicella"[tiab] OR "Rotavirus"[Mesh] OR "Rotavirus Infections"[Mesh] OR "rotavirus"[tiab] OR "Yellow Fever"[Mesh] OR "Encephalitis, Japanese"[Mesh] OR "Dengue"[Mesh] OR "dengue"[tiab] OR "Encephalitis, Tick-Borne"[Mesh])
AND
("serology"[MeSH Terms] OR "serology"[All Fields] OR "serologic tests"[MeSH Terms] OR ("serologic"[All Fields] AND "tests"[All Fields]) OR "serologic tests"[All Fields] OR "serologies"[All Fields] OR "seroepidemiology"[All Fields] OR "seroepidemiologic"[All Fields] OR “seroepidemiologic studies”[Mesh] OR "seroepidemiological"[All Fields] OR "seroprevalence"[All Fields] OR

"seropositive"[All Fields] OR "seroconversion"[All Fields] OR "sero-conversion"[All Fields] OR ("serologic"[All Fields] AND "surveillance"[All Fields]) OR ("serological"[All Fields] AND "surveillance"[All Fields]) OR "serosurvey"[All Fields])
AND
((“residual"[All Fields] OR "stored”[All Fields] OR "leftover"[All Fields] OR "existing"[All Fields] OR "remnant"[All Fields] OR “archived”[All Fields] OR "convenience"[All Fields]) AND ("serum"[All Fields] OR "sera"[All Fields] OR "sample*"[All Fields] OR "specimen*"[All Fields])
OR
“biorepository”[All Fields] OR “biobank*”[All Fields] OR ("serum"[All Fields] AND "bank*"[All Fields]) OR "blood donor*"[All Fields] OR “population-based”[all fields] OR “general population”[all fields])
AND 1990/01/01:2021/06/23[dp]

<u>Embase (first search conducted 6/23/2021; second search conducted on 8/22/2022)</u>
('measles'/exp OR 'measles virus'/exp OR measles:ab,ti OR measles OR 'sars-cov-2'/exp OR 'sars-cov-2':ab,ti OR 'whooping cough'/exp OR pertussis:ab,ti OR 'rubella'/exp OR rubella:ab,ti OR 'papillomaviridae'/exp OR 'human papillomavirus':ab,ti OR 'influenza next/2 human' OR influenza OR 'cholera'/exp OR cholera:ab,ti OR 'neisseria meningitidis'/exp OR 'meningococcal infections'/exp OR 'diphtheria'/exp OR diphtheria:ab,ti OR 'mumps'/exp OR mumps:ab,ti OR 'tetanus'/exp OR 'tetanus toxoid'/exp OR tetanus:ab,ti OR 'hepatitis a'/exp OR 'hepatitis b'/exp OR 'hepatitis e'/exp OR hepatitis:ab,ti OR 'tuberculosis'/exp OR 'pneumococcal infections'/exp OR 'typhoid fever'/exp OR 'poliomyelitis'/exp OR 'haemophilus influenzae type b'/exp OR 'herpes zoster'/exp OR 'herpesvirus 3 next/2 human' OR varicella:ab,ti OR 'rotavirus'/exp OR 'rotavirus infections'/exp OR rotavirus:ab,ti OR 'yellow fever'/exp OR 'japanese encephalitis'/exp OR 'dengue'/exp OR dengue:ab,ti OR 'tick-borne encephalitis'/exp)
AND
('serology'/exp OR serology OR 'serologic tests'/exp OR (serologic NEAR/3 tests) OR 'serolog*' OR 'seroepidemiolog*' OR (‘seroepidemiolog*’ NEAR/3 studies) OR 'seroprevalence'/exp OR seroprevalence OR 'seropositiv*' OR seropositive OR 'seroconversion'/exp OR 'sero conversion' OR (serologic* NEAR/3 surveillance) OR 'serosurvey' OR serosurvey)
AND
(((residual OR stored OR leftover OR existing OR remnant OR archived OR convenience) NEAR/3 (serum OR sera OR sample* OR specimen*)) OR 'biorepository'/exp OR biorepository OR 'biobank*' OR (serum NEXT/2 bank*) OR 'blood donor*' OR ‘population-based’ OR ‘general population’)
AND [1990-2021]/py

<u>Scopus (first search conducted 6/24/2021; second search conducted on 8/22/2022)</u>
TITLE-ABS-KEY("measles" OR "measles virus" OR "sars-cov-2" OR "sars cov 2" OR "whooping cough" OR "pertussis" OR "rubella" OR "papillomaviridae" OR "human papillomavirus" OR "influenza, human" OR "cholera" OR "neisseria meningitidis" OR "meningococcal infections" OR "diphtheria" OR "mumps" OR "tetanus" OR "tetanus toxoid" OR "Hepatitis A" OR "Hepatitis B" OR "Hepatitis E" OR "hepatitis" OR "Tuberculosis" OR "Pneumococcal Infection*" OR "Typhoid Fever" OR "Poliomyelitis" OR "haemophilus influenzae type b" OR "Herpes Zoster" OR "Herpesvirus 3, Human" OR "varicella" OR "rotavirus" OR "Yellow Fever" OR "Japanese Encephalitis" OR "dengue" OR "Tick-Borne Encephalitis")
AND
ALL("serology" OR "serolog* tests" OR ("serologic" W/3 "tests") OR "serolog*" OR "seroepidemiolog*" OR “seroepidemiolog* studies” OR "seroprevalence" OR "seropositive" OR "seroconversion" OR "sero-conversion" OR ("serologic*" W/3 "surveillance") OR "serosurvey")
AND

ALL((“residual" OR "stored” OR "leftover" OR "existing" OR "remnant" OR “archived” OR "convenience") AND ("serum" OR "sera" OR "sample*" OR "specimen*") OR “biorepository” OR “biobank*” OR "serum bank*" OR "blood donor*" OR “population-based” OR “general population”) AND PUBYEAR > 1990

<u>Cochrane (first search conducted 6/28/2021; second search conducted on 8/22/2022)</u>
("measles" OR "measles virus" OR "sars-cov-2" OR "sars cov 2" OR "whooping cough" OR "pertussis" OR "rubella" OR "papillomaviridae" OR "human papillomavirus" OR "influenza, human" OR "cholera" OR "neisseria meningitidis" OR "meningococcal infections" OR "diphtheria" OR "mumps" OR "tetanus" OR "tetanus toxoid" OR "Hepatitis A" OR "Hepatitis B" OR "Hepatitis E" OR "hepatitis" OR "Tuberculosis" OR "Pneumococcal Infection*" OR "Typhoid Fever" OR "Poliomyelitis" OR "haemophilus influenzae type b" OR "Herpes Zoster" OR "Herpesvirus 3, Human" OR "varicella" OR "rotavirus" OR "Yellow Fever" OR "Japanese Encephalitis" OR "dengue" OR "Tick-Borne Encephalitis") in Title Abstract Keyword
AND ("serology" OR "serolog* tests" OR "serologic tests" OR "serolog*" OR "seroepidemiolog*" OR “seroepidemiolog* studies” OR "seroprevalence" OR "seropositive" OR "seroconversion" OR "sero-conversion" OR "serologic* surveillance" OR "serosurvey") in All Text
AND ((“residual" OR "stored” OR "leftover" OR "existing" OR "remnant" OR “archived” OR "convenience") AND ("serum" OR "sera" OR "sample*" OR "specimen*") OR “biorepository” OR “biobank*” OR "serum bank*" OR "blood donor*" OR “population-based” OR “general population”) in All Text

Search strategy
Screening was first conducted through an online PubMed search for English-language and French-language literature published between January 1990 and June 2021. Between June 2021 and July 2021, we searched several electronic databases with reference to the expanded Medical Subject Headings (MeSH) thesaurus, using search terms for vaccine-preventable diseases (VPDs), serologic testing, and residual samples. The databases included the following: PubMed, Scopus, Embase, Cochrane, and the WHO iris database. Additional studies were identified through manual searches of the reference lists of identified papers including systematic reviews and papers reviewing biobank utility. Suggestions from experts in the field and investigations from two international organizations (CDC and WHO) were consulted.

Screening Strategy
After deleting duplicates, two members of the literature review group systematically screened the title and abstract of papers identified in the electronic searches for the inclusion and exclusion criteria using Covidence. Those that met the criteria or have remaining uncertainty for the criteria were read in full text. Full text screening was done by two reviewers with all disagreements resolved through discussion and arbitration with a third reviewer. Prior to the final analysis, the search was re-run (August 2022) to include studies published while reviewers conducted the initial screening.

Condition or domain being studied
The scoping review aims to describe the uses of residual sera samples in serological testing studies of VPDs.

Participants/Population
Humans of all ages, regardless of location and characteristics.

Main Outcome(s)
The purpose for using residual samples in the study, i.e., seroprevalence of a pathogen. The following will be considered to inform the development of key themes describing the use of residual sera samples:

- VPD of interest
- Country and region from where samples were collected
- Collection and storage site of specimen
- Specimen type (venous or finger-prick)
- Sample size
- Original use for the samples
- Study year(s)
- Year(s) of original sample collection
- Population from where samples were collected
- Serological tests that were performed
- Statement on permissions and ethical considerations associated with using residual sera
- Meta-data of the residual sample
- Objectives of the paper
- If and how authors talk about bias of residual specimens
- If collection was part of a larger serological surveillance system

Data Extraction
A standardized data extraction form will be developed in Kobo Toolbox and piloted on 5-10 known publications. Any issues will be addressed, and the final version will be used. One reviewer will conduct data extraction. A second reviewer will be consulted if questions arise about adherence to inclusion and exclusion criteria or the data extraction process. The following data will be extracted: publication details, type of study, study design and methods, results, and methodological quality items according to the type of study. The main outcomes will be extracted from publications in the following manner:

Identification

- Publication year
- Country and region: codelist [country codes]
- Study year(s): codelist [1980 – 2022]
- Year(s) of original sample collection: dropdown [1900 – 2022]

Population

- Population that sample was collected from: codelist [general population, pregnant women, blood donors, plasma donors, patient population, school children, injection drug users, non-injecting drug users, military, MSM, homeless population, healthcare workers, tattoo artists, indigenous people, migrants, inmates/incarcerated individuals, university students, not specified]
- Details of the original population: free text
- Original use for the samples: codelist [blood donation, plasma donation, seroprevalence study, diagnostic sample, other/non-serological survey]
- Collection site of original sample: codelist [household, school, hospital/clinic, blood or plasma donation site, diagnostic center/laboratory, homeless shelter, prison, unknown]
- Storage site of residual specimens: codelist [research biorepository, blood/plasma donation center biorepository, diagnostic center/laboratory biorepository, unspecified biorepository]
- Total sample size of the whole analysis: free text
- Sample size of residual specimens: free text
- Specimen type: codelist [fingerprick, venous]

Methods

- Paper objective: codelist [to describe population seropositivity, to describe seroprevalence among a specific clinical subpopulation, to evaluate the need for blood donor screening, to describe antibody kinetics following vaccination (e.g., vaccine effectiveness), to describe antibody kinetics following natural infection (e.g., immunogenicity of infection), to estimate infection rates or

understand transmission dynamics, to compare the use of residual samples to population-based samples, to identify risk factors of seropositivity, to evaluate outcomes of seropositivity, to evaluate changes in seropositivity overtime, to evaluate cross-reactivity, to describe viral dynamics, to compare population seropositivity between countries or international regions]

- VPD of interest is main objective or subobjective: binary [yes, no]
- Residual sample came from a national sero-surveillance system or a national serial cross-sectional serosurvey: binary [yes, no]
  - If yes: name the national sero-surveillance system or a national serial cross-sectional serosurvey
- Inclusion criteria for residual samples: free text
- Exclusion criteria for residual samples: free text
- Serological tests that were performed: codelist [enzyme-linked immunosorbent assay or enzyme-based immunoassay (ELISA or EIA), hemagglutination inhibition (HI) assay, neutralization test (NT or PRNT), chemiluminescence immunoassay (CLIA), time-resolved fluoroimmunoassay (TR-FIA), radioimmunoassay/immunofluorescence technique (IF), multiplex immunoassay, lateral flow rapid test, epitope-blocking assay, isoelectric focusing, Western blot, latex agglutination test, protein microarray, fluorescent-antibody-to-membrane-antigen (FAMA) test, toxin binding inhibition assay]
- VPD of interest: codelist [cholera, dengue, diptheria, HAV, HBV, HEV, Hib, HPV, influenza, Japanese encephalitis, measles, mumps, Neisseria meningitis, pertussis, pneumococcal, polio, rotavirus, rubella, Sars-CoV-2, tetanus, tick-borne encephalitis, tuberculosis, typhoid, varicella/herpes zoster, yellow fever]
- Meta-data linked to residual samples and used in the analysis: codelist [basic demographic data (age, sex, race, ethnicity), extended demographic data (e.g., residence, occupation, SES), epidemiologic data on VPD of interest (e.g., vaccination status, history of infections), epidemiologic data not on VPD of interest (e.g., history or current infection status of other diseases, biomarkers), none]

Quality Assessment

- Authors included a statement about ethical considerations associated with retesting specimens: codelist [approved by an ethics committee/board, broad consent given at time of specimen collection, consent given in retrospect, received waiver on re-consenting, samples deidentified/anonymized, exempt as public health surveillance, did not include a statement]
- Acknowledgement of bias from residual samples: codelist [no mention of bias, acknowledged bias, residual sample not biased (because the target population of the residual sample is the same as the target population of the original sample)]
- Handling of bias: codelist [conducted stratified subsampling, used inclusion/exclusion criteria for the samples, conducted stratified analysis, weighted final results, compared to alternative data source for "validity", compared to other published estimates, tested samples multiple times for the same pathogen, conducted sensitivity analysis, did not account for bias/none of the above]

Strategy for data synthesis

Data synthesis will be descriptive. The scoping review will be a narrative on the uses of residual samples in serologic studies. VPDs will be categorized based on primary mode of transmission for data synthesis (airborne, vector-borne, food- or water-borne, human physical contact, other physical contact). The data synthesis will include enumeration of the main outcomes and presented in tables, maps, and graphs.

Text S2: Preferred Reporting Items for Systematic reviews and Meta-Analyses extension for Scoping Reviews (PRISMA-ScR) Checklist

SECTION	ITEM	PRISMA-ScR CHECKLIST ITEM	REPORTED ON PAGE #
**TITLE**			
Title	1	Identify the report as a scoping review.	See Title Page
**ABSTRACT**			
Structured summary	2	Provide a structured summary that includes (as applicable): background, objectives, eligibility criteria, sources of evidence, charting methods, results, and conclusions that relate to the review questions and objectives.	See Abstract
**INTRODUCTION**			
Rationale	3	Describe the rationale for the review in the context of what is already known. Explain why the review questions/objectives lend themselves to a scoping review approach.	Reported in introduction section
Objectives	4	Provide an explicit statement of the questions and objectives being addressed with reference to their key elements (e.g., population or participants, concepts, and context) or other relevant key elements used to conceptualize the review questions and/or objectives.	Reported in introduction section
**METHODS**			
Protocol and registration	5	Indicate whether a review protocol exists; state if and where it can be accessed (e.g., a Web address); and if available, provide registration information, including the registration number.	Reported in method section that protocol exists in supplementary materials (Text S1)
Eligibility criteria	6	Specify characteristics of the sources of evidence used as eligibility criteria (e.g., years considered, language, and publication status), and provide a rationale.	Summarized in methods section and detailed in protocol (Text S1)
Information sources*	7	Describe all information sources in the search (e.g., databases with dates of coverage and contact with authors to identify additional sources), as well as the date the most recent search was executed.	Summarized in methods section and detailed in protocol (Text S1)
Search	8	Present the full electronic search strategy for at least 1 database, including any limits used, such that it could be repeated.	Included in the protocol (Text S1)
Selection of sources of evidence†	9	State the process for selecting sources of evidence (i.e., screening and eligibility) included in the scoping review.	Summarized in methods section and detailed in protocol (Text S1)
Data charting process‡	10	Describe the methods of charting data from the included sources of evidence (e.g., calibrated forms or forms that have been tested by the team before their use, and whether data charting was done independently or in duplicate) and any processes for obtaining and confirming data from investigators.	Summarized in methods section and detailed in data extraction survey (Text S3)

Data items	11	List and define all variables for which data were sought and any assumptions and simplifications made.	Summarized in methods section and detailed in data extraction survey (Text S3 and S4) and supplementary material (Table S1-S3)
Critical appraisal of individual sources of evidence§	12	If done, provide a rationale for conducting a critical appraisal of included sources of evidence; describe the methods used and how this information was used in any data synthesis (if appropriate).	Articles were not critically appraised; stated in the methods section
Synthesis of results	13	Describe the methods of handling and summarizing the data that were charted.	Reported in the methods section and supplementary material (Table S1-S3)
**RESULTS**			
Selection of sources of evidence	14	Give numbers of sources of evidence screened, assessed for eligibility, and included in the review, with reasons for exclusions at each stage, ideally using a flow diagram.	Summarized in results section and detailed in Figure 1
Characteristics of sources of evidence	15	For each source of evidence, present characteristics for which data were charted and provide the citations.	Summary of the articles organized by VPD is included in supplementary materials (Table S4)
Critical appraisal within sources of evidence	16	If done, present data on critical appraisal of included sources of evidence (see item 12).	Not applicable
Results of individual sources of evidence	17	For each included source of evidence, present the relevant data that were charted that relate to the review questions and objectives.	Summary of the articles organized by VPD is included in supplementary materials (Table S4)
Synthesis of results	18	Summarize and/or present the charting results as they relate to the review questions and objectives.	Reported throughout the results section, Table 1, Figure 3-6, and supplementary materials (Text S5, Figures S1-S3, Tables S5-S9)
**DISCUSSION**			
Summary of evidence	19	Summarize the main results (including an overview of concepts, themes, and types of evidence available), link to the review questions and objectives, and consider the relevance to key groups.	Reported throughout the discussion section
Limitations	20	Discuss the limitations of the scoping review process.	Reported in discussion section
Conclusions	21	Provide a general interpretation of the results with respect to the review questions and objectives, as well as potential implications and/or next steps.	Reported in discussion section
**FUNDING**			
Funding	22	Describe sources of funding for the included sources of evidence, as well as sources of	Reported in funding section

		funding for the scoping review. Describe the role of the funders of the scoping review.	

JBI = Joanna Briggs Institute; PRISMA-ScR = Preferred Reporting Items for Systematic reviews and Meta-Analyses extension for Scoping Reviews.

* Where *sources of evidence* (see second footnote) are compiled from, such as bibliographic databases, social media platforms, and Web sites.

† A more inclusive/heterogeneous term used to account for the different types of evidence or data sources (e.g., quantitative and/or qualitative research, expert opinion, and policy documents) that may be eligible in a scoping review as opposed to only studies. This is not to be confused with *information sources* (see first footnote).

‡ The frameworks by Arksey and O'Malley (6) and Levac and colleagues (7) and the JBI guidance (4, 5) refer to the process of data extraction in a scoping review as data charting.

§ The process of systematically examining research evidence to assess its validity, results, and relevance before using it to inform a decision. This term is used for items 12 and 19 instead of "risk of bias" (which is more applicable to systematic reviews of interventions) to include and acknowledge the various sources of evidence that may be used in a scoping review (e.g., quantitative and/or qualitative research, expert opinion, and policy document).

From: Tricco AC, Lillie E, Zarin W, O'Brien KK, Colquhoun H, Levac D, et al. PRISMA Extension for Scoping Reviews (PRISMAScR): Checklist and Explanation. Ann Intern Med. 2018;169:467–473. doi: 10.7326/M18-0850.

Text S3: Kobo Toolbox Data Extraction Survey

Residual Sera Scoping Review – Data Extraction Questions

1. Title of study
2. Last name of first author
3. Publication year
4. Covidence number
5. Year(s) that study was conducted: [1900 – 2022]
6. Study country(s): [list country codes]
7. Is a VPD the main objective of the research paper? Yes / No.
8. Did the residual sample come from a national sero-surveillance system or a national serial cross-sectional serosurvey? Yes / No. If Yes, specify.
9. Population that the residual sample was collected from:
   - General population
   - Pregnant women
   - Blood donors
   - Plasma donors
   - Patient population
   - School children
   - Injection drug users
   - Non-injecting drug users
   - Military
   - MSM
   - Homeless population
   - Healthcare workers
   - Tattoo artists
   - Indigenous people
   - Migrants
   - Inmates/Incarcerated individuals
   - University students
   - Not specified
   - Other: specify
10. Provide details of the original population.
11. Original use for samples:
    - Blood donation
    - Plasma donation
    - Serosurvey
    - Diagnostic sample (including routine testing and chronic patients)
    - Other/non-serological survey
    - Other: specify
12. Did the residual population have inclusion criteria? Yes / No. If Yes, provide details.
13. Did the residual population have exclusion criteria? Yes / No. If Yes, provide details.
14. Collection site of residual specimens:
    - Household

School
Hospital/clinic
Blood donation site
Plasma donation site
Diagnostic center/laboratory
Homeless shelter
Prison
Unknown
Other: specify

15. Storage site of residual specimens:
    Unspecified biorepository
    Research biorepository
    Blood/plasma donation center biorepository
    Diagnostic center/laboratory biorepository

16. Individual meta-data linked to residual samples and used in the analysis:
    Basic demographic data (age, sex, race, ethnicity)
    Extended demographic data (e.g., residence, occupation, SES)
    Epi data on VPD of interest (e.g., vaccination status, history of infections)
    Epi data not on VPD of interest (e.g., history or current infection status of other diseases, biomarkers)
    None

17. Did the authors include a statement about ethical considerations with retesting specimens?
    Yes, approved by an ethics committee/board
    Yes, broad consent given at time of specimen collection
    Yes, consent given in retrospect
    Yes, received waiver on re-consenting
    Yes, samples de-identified/anonymized
    Yes, exempt as public health surveillance
    Yes, other: specify
    No

18. Total sample size of the study

19. Sample size of residual specimens

20. Sample size of the original sample from which residual sample was pulled

21. Specimen type:
    Fingerprick
    Venous

22. Serological tests performed:
    Neutralization test
    Hemagglutination inhibition assay
    Enzyme-linked immunosorbent assay or enzyme-based immunoassay (ELISA or EIA)
    Chemiluminescence immunoassay (CLIA)
    Time-resolved fluoroimmunoassay (TR-FIA)
    Radioimmunoassay, immunofluorescence technique (IF)
    Multiplex immunoassay
    Lateral flow immunoassay (rapid test)
    Epitope-blocking assay using monoclonal antibodies
    Isoelectric focusing

Immunoblot (Western blot)
Latex agglutination (LA) test
Protein microarray (PA)
Fluorescent-Antibody-to-Membrane-Antigen (FAMA) test
Toxin binding inhibition assay
Other: specify

23. VPD(s) of interest:
    Cholera
    Dengue
    Diphtheria
    Hepatitis A (HAV)
    Hepatitis B (HBV)
    Hepatitis E (HEV)
    Hemophilus influenza (Hib)
    Human Papillomavirus (HPV)
    Influenza
    Japanese Encephalitis
    Measles
    Mumps
    Neisseria Meningitis (meningococcal meningitis)
    Pertussis (whooping cough)
    Pneumococcal
    Poliomyelitis (polio)
    Rotavirus
    Rubella
    Sars-CoV-2 (COVID-19)
    Tetanus
    Tick-borne Encephalitis
    Tuberculosis
    Typhoid (Salmonella Typhi or typhoid fever)
    Varicella/Herpes Zoster (shingles & chicken pox)
    Yellow Fever

24. Acknowledgement of bias from residual samples:
    No mention of bias
    Acknowledged bias
    Residual sample not biased (b/c the target population of the residual sample is the same as the target population of the original sample)

25. Exploring bias of residual samples:
    Conducted stratified subsampling
    Used inclusion/exclusion criteria for the samples to address the bias
    Conducted stratified analysis (results)
    Weighted final results
    Compared it to alternative data source for "validity"
    Compared to other published estimates (discussion)
    Did not account for bias/none of the above
    Conducted sensitivity analysis

26. Paper objective (identified in title/abstract):
    to describe population seropositivity
    to describe seroprevalence among a specific clinical subpopulation

to evaluate the need for blood donor screening (e.g., HEV)
to describe antibody kinetics following vaccination (e.g., vaccine effectiveness)
to describe antibody kinetics following natural infection (e.g., immunogenicity of infection)
to estimate infection rates or understand transmission dynamics
to compare the use of residual samples to population-based samples
to identify risk factors of seropositivity
to evaluate outcomes of seropositivity
to evaluate changes in seropositivity overtime
to evaluate cross-reactivity
to describe viral dynamics
to compare population seropositivity between countries or international regions
Other: specify

27. Other comments

Text S4: Defining Serological Surveillance System

In question #8 above of the data extraction tool, we asked “Did the residual sample come from a national sero-surveillance system or a national serial cross-sectional serosurvey? Yes / No. If Yes, specify.” We began with a short list of surveillance sero-surveillance systems and national serial cross-sectional serosurveys we were aware of. Then we added onto the list as data extraction continued. In the end we delineated the following:

1. Unspecified serial cross-sectional survey
2. Unspecified sero-surveillance system
3. NHANES (USA)
4. PIENTER (Netherlands)
5. ESEN (European) Studies
6. KiGGS (Germany)
7. NCIRS (Australia)
8. HPA sero-surveillance/seroepidemiology unit (England) - formerly PHLS Serological Surveillance Programme
9. UK biobank cohort
10. Vietnam sero-surveillance (serum biobank project)
11. CIRN (Canada)
12. ID Surveillance Center or National Serum Reference Bank (SRB) from National Institute of Infectious Diseases (Japan)
13. Israel national serum bank by Center for Disease Control (ICDC)
14. Blood and Organ Transmissible Infectious Agents (BOTIA) project
15. U.S. Defense Medical Surveillance System (DMSS) and Department of Defense Serum Repository
16. Hungarian serial cross-sectional sero-surveillance (National Centre of Epidemiology)
17. Finnish Maternity Cohort (FMC)
18. Singapore National Health Survey (NHS)
19. Iceland Maternity Cohort
20. National Health and Nutrition Survey (ENSANUT) Mexico
21. German Health Interview and Examination Survey for Adults (DEGS)
22. KNHANES (Korea)
23. National Pediatric Seroprevalence Survey (NPSS) Singapore
24. JANUS (Norway) cancer registry
25. GUMCAD STI Surveillance System (England)
26. German National Nutrition Survey (NVS)
27. Retrovirus Epidemiology Donor Study (REDS) Allogenic Donor Recipient (RADAR) repository
28. Swiss HIV Cohort Study (SHCS)
29. Israel Defense Force Health Surveillance Bank
30. US National Cancer Institute (NCI) cohort consortium
31. Diabetomobile (Germany)
32. Other

Text S5: Supplemental Results

Serosurvey objectives

Within the article abstract or summary, researchers identified one or more of twelve study objectives delineated in Table S2. Figure 3 shows the percent of studies that report each objective overall and by the top 6 VPDs. Most articles (80.5%) specified at least one objective was to describe population seroprevalence (e.g., Ekong et al 2022 estimated dengue seroprevalence in Nigeria using stored specimens originally tested for acute brucellosis or malaria infection [7]). Over half of articles that did not have this objective described seroprevalence among a specific clinical subpopulation (e.g., Toyoda et al. 2008 used stored serum samples from hemophilia patients to assess the prevalence of HEV antibody in Japanese patients with hemophilia [213]) (Table S5). Other objectives were to identify risk factors of seropositivity (33.3%) (e.g., Chin et al 1991 evaluated age, sex, and socio-economic risk factors of hepatitis A seropositivity in Hong Kong by using residual specimens originally tested for hepatitis B [55]), estimate parameters relating to magnitude or timing of infection such as incidence, prevalence, force of infection (17.6%) (e.g., Routledge et al 2022 tested for COVID-19 seroprevalence using residual specimens from blood draws to estimate the probability of prior infection among geographic regions in San Francisco, USA [534]), and/or evaluate changes in seroprevalence over time (15.8%). A significantly larger proportion of influenza, HPV, and Hepatitis E studies evaluate cross-reactivity, evaluate clinical outcomes of seropositivity, and evaluate the need for blood donor screening, respectively (Figure 3).

Sources of residual specimens

The main source of residual specimens for all articles as well as those studying COVID-19, hepatitis B, HPV, influenza, and measles was from patient populations (Figure S1). However, hepatitis E articles used blood and plasma donor samples more than patient samples (Figure S1). Residual blood from general populations were the second or third most used source of specimens for all VPD groups, particularly for HPV and influenza (Figure S1). As defined in Table S1, we categorized specimens from general populations if they were representative of a country, region or other geographic unit (e.g., Ang et al. 2015 tested residual specimens for dengue seropositivity that were originally collected as part of Singapore’s population-based National Health Survey to determine the prevalence of chronic conditions among adults [24]) or not otherwise specified by other source populations such as vaccine trials or cohort studies (e.g., Thiry et al. 2002 used specimens from a serum bank collected from Belgium volunteers in a vaccine trial to test for varicella zoster virus seropositivity [25]). The proportion of articles using samples from vulnerable populations and drug users was higher for hepatitis B.

Over time, specimens collected from patients for routine or specialized testing made up the highest proportion of original populations per year between 2004 to 2018 (Figure S2A). Before 2004, blood and plasma donors were equal or greater in proportion than patients, but after 2018, blood and plasma donors were used in comparable amounts to patients (Figure S2A). Over time, clinical testing and blood or plasma donations were the leading purposes for original specimen collection (Figure S2B). Serosurveys as a purpose for original data collection are consistent over time (Figure S2B).

Time since collection

Articles tended to use recently collected specimens, as the mean difference in years between publication and original sample collection was 6.5 years (Figure S3A). Eighty percent and ninety-four percent of articles used specimens collected within 10 or 20 years of publication, respectively. The mean number of years between data collection and publication for COVID-19 was 1.7 years (Figure S3B).

Figure S1. Bar plot displaying percent of studies by original sample population for all VPDs and for the six most studied VPDs (N = 601 studies). Note, the categories below are not mutually exclusive meaning that one study could have conducted testing for multiple pathogens and have had multiple original sample population. Also note that occupational populations include military, healthcare workers, farmers, and other occupational cohorts, and vulnerable populations include indigenous people, inmates/incarcerated individuals, homeless population, men who have sex with men (MSM), and migrants.

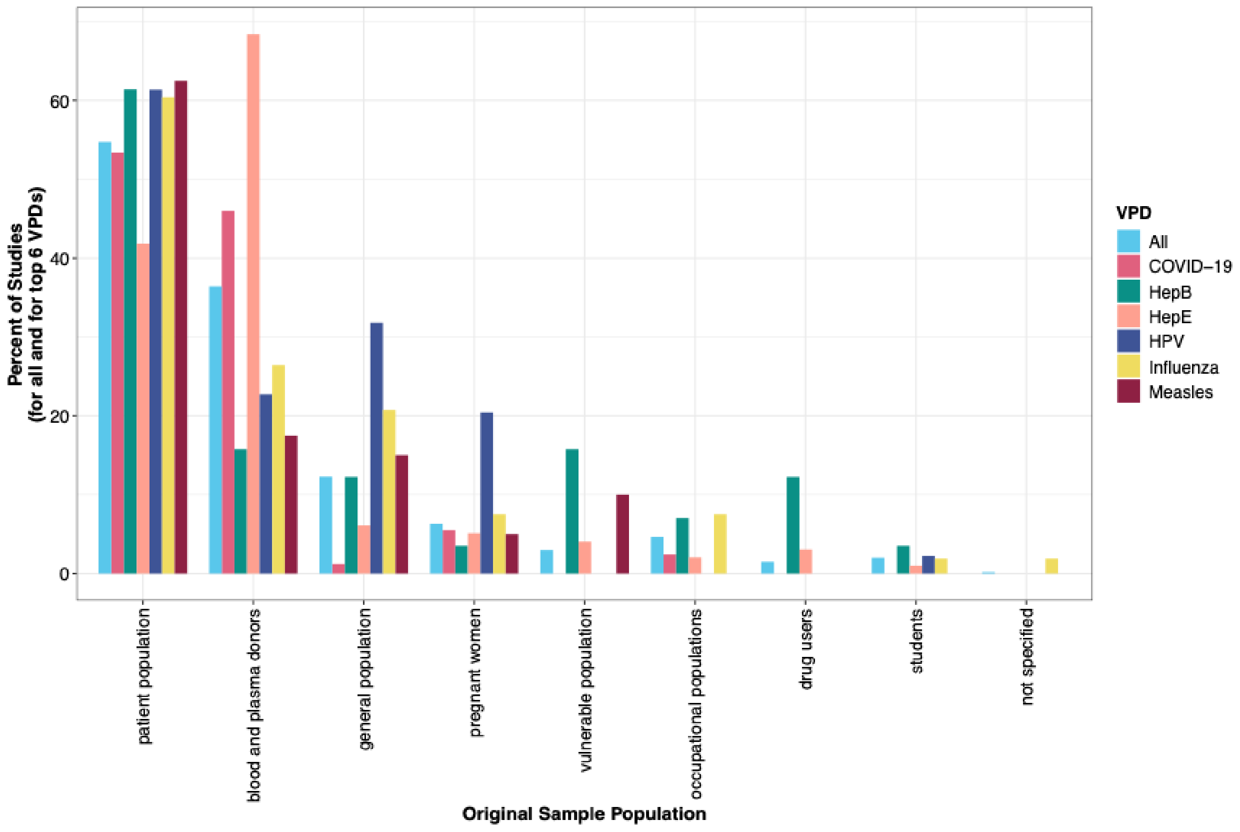

Figure S2. Time series of studies by publication year based on A) original source population specified in the studies and B) original use for specimens specified in the studies (N = 601 studies).

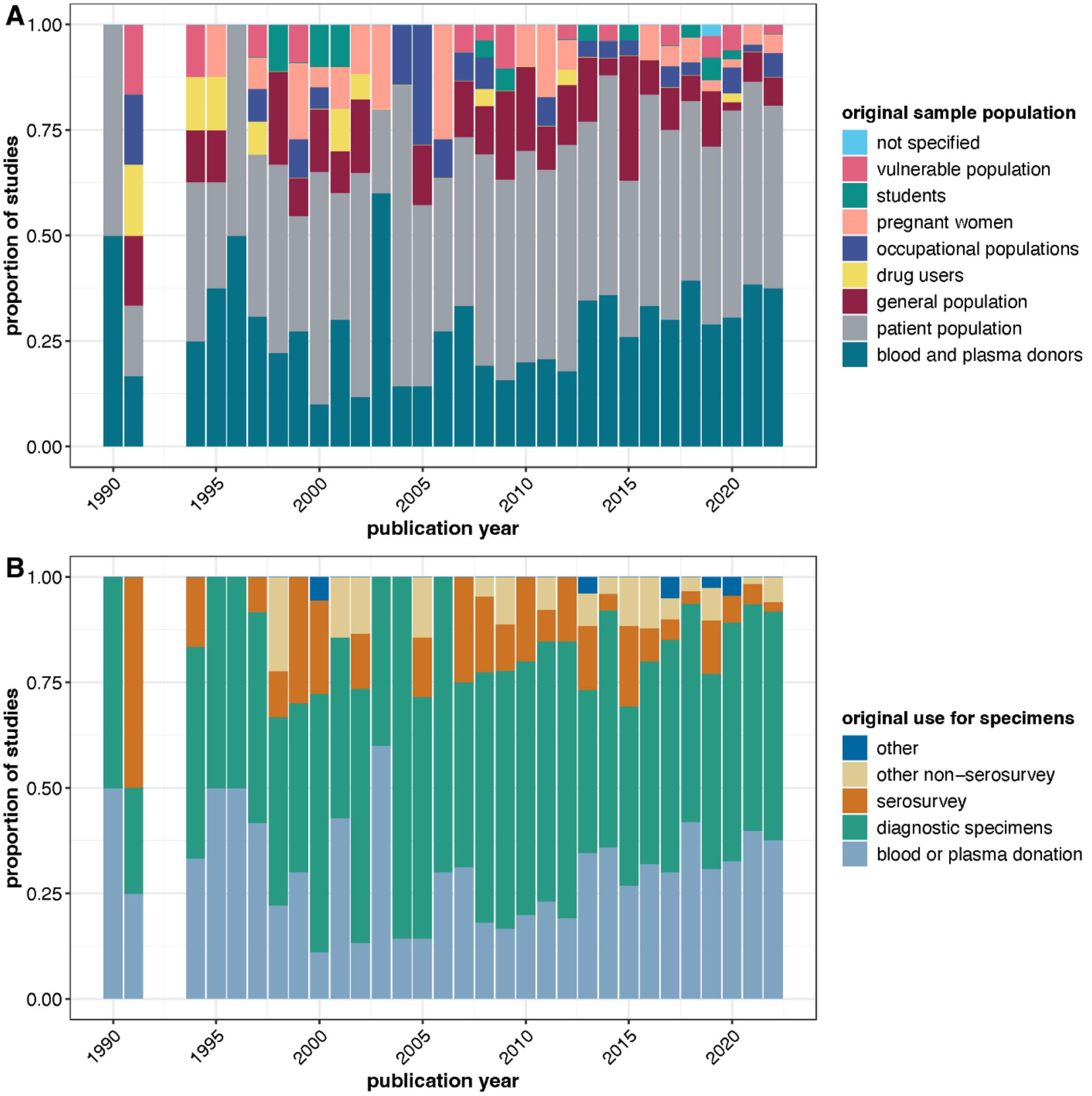

Figure S3. Histogram of difference between publication year and year of original sample collection. A) All studies (N = 568 studies), note not all studies listed the year of original sample collection. B) By six most studies VPDs. The red line and number represent the mean number of years.

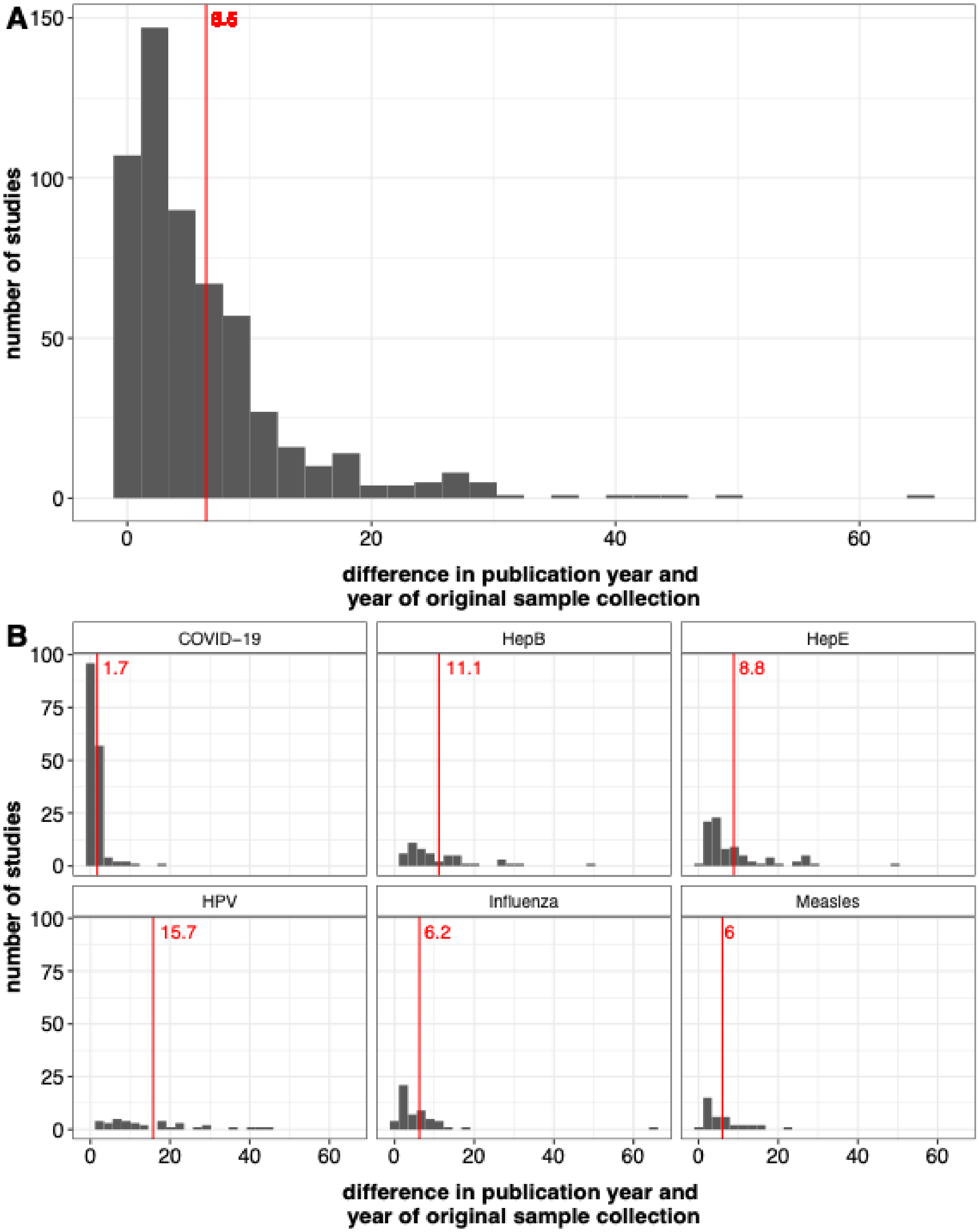

Table S1. Handling of paper objectives based on article's abstract

Original population	Description
Describe population seropositivity	Most articles fell into this category, either alone or in combination with other objectives. If the paper was descriptive of seroprevalence, or if no other objectives were clear, or very limited information in the abstract, then this objective was selected alone.
Identify risk factors of seropositivity	Abstracts included associations with characteristics of interest (e.g., p-value or odds ratios reported, mention of significant or non-significant differences).<br>If stratified seroprevalence was described in the abstract without an indication the authors conducted a test or were evaluating an association then this objective was not selected.
Estimate parameters related to the magnitude or timing of infection or understand transmission dynamics	Abstracts included incidence rates, force of infection, prevalence of recent infection (e.g., pertussis, dengue) and other transmission dynamics estimated using IgG alone or in combination with IgM.<br>If estimates were based only on IgM results and studies where the infection rates described were based on surveillance data then this objective was not selected.
Evaluate changes in seropositivity over time	Specimens from multiple timepoints tested as part of the study where at least one time point included residual specimens.<br>If residual specimens from a single time point were qualitatively compared with seroprevalence findings from other studies then this objective was not selected.
Describe seroprevalence among a clinical subpopulation	Question of interest was focused on the clinical subpopulation (e.g., seroprevalence among patients with renal cancer or people living with HIV).<br>Pregnant women were not considered clinical subpopulations. If the clinical subpopulation was one of many populations with no direct interest in the findings for that clinical subpopulation then this objective was not selected.

Evaluate health or disease outcomes associated with seropositivity	Association between seropositivity and development of outcomes such as cancer, liver disease, HIV, and severity of the VPD of interest.
Evaluate the need for blood donor screening	Studies to evaluate if screening for a VPD should be conducted in settings where it was not already routine practice.
Evaluate antibody cross-reactivity	Cross-reactivity of virus variants (e.g., influenza or SARS-CoV-2) or between viruses (e.g., West Nile, yellow fever, Japanese encephalitis).
Describe antibody kinetics following vaccination	Given known vaccination status of individuals, e.g., recorded evidence or individuals who were part of a prior study that administered the vaccine, the study evaluated quantitative antibody concentrations following vaccination.<br>Studies comparing infection-induced and vaccine-induced antibody responses fell into both categories (antibody kinetics following vaccination and antibody kinetics following infection).
Describe antibody kinetics following infection	Given known infection status of individuals, the study evaluated quantitative antibody concentrations following infection.<br>Studies comparing infection-induced and vaccine-induced antibody responses fell into both categories (antibody kinetics following vaccination and antibody kinetics following infection).
Compare population seropositivity between countries or international regions	Smallest administrative unit included for this objective was at the country-level.<br>If comparisons were between regions, districts, states, etc. within the country then this objective was not selected.
Compare the use of residual samples to population-based samples	Authors tested specimens from both residual and population-based sources and reported on the comparison.<br>If residual specimens were qualitatively compared with seroprevalence findings from separate population-based study then this objective was not selected.

Describe viral dynamics	Investigating how a virus changes over time (viral strains or lineages). Most relevant for influenza and SARS-Cov-2.<br>If the study estimated serotype/strain/variant at a given time point (cross-sectional study) then this objective was not selected.

Note 1: Objective of residual specimen serological testing categories are not mutually exclusive.
Note 2: Objective for the residual specimens only as specified in the article's abstract. Objectives for any non-residual specimens that were mentioned in the article's abstract were not captured in data abstraction.
Note 3: We ignored any policy-specific objectives, such as evaluating effectiveness of a mass vaccination program, and abstracted any other objectives in the paper or treated them as 'describe general seropositivity'.

Table S2. Examples of original sample populations

**Original population**	**Examples**
Patient populations	Definitions: Specimens from routine or specialized laboratory testing from health facilities or diagnostic laboratories;<br>Examples: included clinical subpopulations such as cancer patients or patients on dialysis, patients seeking emergency care, patients attending a specialty health clinic (e.g., HIV clinic), patients with the VPD of interest (e.g., SARS-CoV-2).<br>*Note: Patient populations often used as convenience samples. If it was unclear if the specimen was residual then the article was excluded at the full-text screening stage.*
Blood and plasma donors	Definition: Specimens collected from blood or plasma donors as part of routine screening.<br>*Notes:*<br>*Blood and plasma donors were often used as convenience samples. If it was unclear if the specimen was residual then the article was excluded at the full-text screening stage.*<br>Excluded testing of blood or plasma donors for VPDs as part of routine screening within that country; policies for routine screening varied by country and timepoint.<br>Excluded studies where blood or plasma donors were only used as a control group to compare with a non-residual specimen source.<br>Excluded convalescent plasma donors for SARS-CoV-2.
Pregnant women	Definition: Specimens collected from pregnant women.<br>Examples: Residual specimens from routine antenatal care testing or cord blood collected at delivery, and pregnant women who were part of a cohort or prior study.<br>Note:<br>*Pregnant women patients often used as convenience samples. If it was unclear if the specimen was residual then the article was excluded at the full-text screening stage.*<br>Excluded routine testing for VPDs (e.g., rubella).
Drug users (injection and non-injection)	Definitions: Specimens collected from drug users (injection and non-injection).

	Examples: Residual specimens from drug treatment centers or health clinics, and specimens from drug users collected as part of a cohort or prior study.
Students	Definition: Specimens collected from students in a school setting (pre-primary, primary, secondary or university).<br>Examples: Residual specimens collected from a prior study (e.g., vaccination coverage or anemia studies), collected as part of a vaccination program (e.g., prevaccination rubella testing) or collected during a medical check-up of school children.
Vulnerable populations	Vulnerable populations: Specimens collected from indigenous populations, migrants, incarcerated individuals, MSM, homeless individuals.<br>Examples: Residual specimens collected during health examinations for asylum seekers or migrants, intake screening of incarcerated individuals, residual specimens from a prior study, or residual specimens collected at a health clinic.
Occupational populations	Definitions: Specimens collected from specified occupational populations.<br>Examples: Residual specimens from pre-employment medical assessments (e.g., from healthcare workers, military) and residual specimens collected from occupational populations during a prior study (e.g., pig farmers, fish mongers, tattoo artists).
General population	Definition: Specimens collected from populations representative of a country, region or other geographic unit, or not otherwise specified above.<br>Examples: Residual specimens from prior studies (e.g., population-based HIV seroprevalence study).<br>Other research studies such as cohort studies or vaccine clinical trials where the population does not fit into one of the other categories.

Note: Population categories were not mutually exclusive.

Table S3. Handling of selection bias in papers using residual specimens

Stage	Handling of bias	Description	Authors' opinion on the ease and value of each approach*
Interpretation	Compared to other published estimates	Qualitatively compared seroprevalence findings from the residual specimens with that from other published estimates or administrative sources, typically done in the Discussion section.	Low difficulty.<br>Low relative importance<br>Can lend some external validity of the findings, but of little reliability
Analysis	Conducted stratified analysis	Stratified seroprevalence estimates by characteristics associated with seropositivity because if the distribution of the characteristics are not representative of the population of interest then the overall estimate of seroprevalence may be biased (e.g., age, geographic area).	Low difficulty.<br>High relative importance.<br>This approach requires linked meta-data but is an important first step if assume that the samples are not representative across all strata.
Design	Conducted stratified subsampling	Purposefully over or undersampled from specified subgroups to obtain a study population more representative of the target population (e.g., limit the number of specimens from a specific medical ward at a facility)	Medium difficulty.<br>High relative importance.<br>This approach requires linked meta-data as well as knowledge of population distributions of the strata of interest.
Design	Used inclusion/exclusion criteria for residual samples	Restricted selection of residual specimens by certain characteristics that may influence seroprevalence to obtain a study population more representative of the target population (e.g., exclude specimens from a specific medical ward at a facility, or excluded specimens admitted for respiratory illness).	Medium difficulty.<br>High relative importance.<br>This approach requires linked meta-data of characteristics that likely result in biased sample.
Analysis	Weighted or standardized final results	Seroprevalence estimates are weighted or standardized to align with the distribution of characteristics in the target population (e.g., age, gender, geographic distribution).	Medium difficulty.<br>High relative importance.<br>This approach requires linked meta-data as well as knowledge of population distributions of the strata of interest.
Analysis	Quantitatively compared to	Quantitatively compared seroprevalence findings from the residual specimens with that from other seroprevalence estimates,	High difficulty.<br>High relative importance.

	alternative data source	typically done in the Results section. Some instances may have included formal statistical comparative tests.	If possible, being able to quantify the amount of bias is very valuable and can allow for extrapolation to other studies with the same specimen source.
Analysis	Conducted sensitivity analysis	Reanalysis varying how the input data were handled or other assumptions or parameters used in models.<br>Sensitivity analysis varying thresholds or how equivocal specimens were treated (e.g., treating as positive in primary analysis and negative in sensitivity) was not considered a method of handling of bias.	Low difficulty.<br>Medium relative importance. The importance of this approach depends highly on the research question at hand.
N/A	No selection bias because the residual sample represented the population of interest	Seroprevalence estimated from residual specimens originating from the population of interest (e.g., Hepatitis E serosurvey from residual blood donor specimens to evaluate the need to screen blood donors for Hepatitis E).	

Note: Handling of selection bias categories were not mutually exclusive.
* Difficulty was assigned based on authors' opinion and experience with implementing residual clinical specimen serosurveys. Difficulty was based on logistics and operational considerations. Relative importance was based on how likely to address selection bias. Categories were defined as low, medium, and high

Table S4. **Studies selected for data extraction and analysis (N=601 articles)**

**VPD**	**References**
Dengue	[1-31]
Diphtheria	[32-50]
Hepatitis A (HAV)	[45, 51-88]
Hepatitis B (HBV)	[5, 44, 45, 49, 53, 58, 62, 77, 79, 82, 89-135]
Hepatitis E (HEV)	[53, 59, 60, 62, 69, 77, 80, 82-84, 114, 136-222]
Hemophilus influenza type B (Hib)	[223-225]
Human papillomavirus (HPV)	[226-269]
Influenza	[270-322]
Japanese Encephalitis	[3, 14, 323, 324]
Measles	[38, 44, 45, 49, 56, 108, 325-358]
Mumps	[38, 45, 108, 327, 331, 347, 352, 355, 357, 359-366]
Meningitis	[367, 368]
Pertussis	[39, 369-384]
Pneumococcal	
Poliomyelitis	[39, 385-391]
Rotavirus	[392]
Rubella	[38, 44, 45, 49, 108, 327, 329, 331, 332, 339, 342, 343, 352, 355-357, 393-409]
Sars-CoV-2	[410-572]
Tetanus	[33, 34, 38, 41, 44, 50, 368]
Tick-borne Encephalitis	[573, 574]
Tuberculosis	
Typhoid	[575]
Varicella/Herpes Zoster	[44, 45, 49, 61, 108, 329, 576-599]
Yellow Fever	[14, 600, 601]

1. Ang, L.W., et al., *Seroprevalence of past dengue virus infection among children and adolescents in Singapore.* J Med Virol, 2015. **87**(12): p. 2159-62.
2. Ang, L.W., et al., *Seroepidemiology of dengue virus infection in the adult population in tropical Singapore.* Epidemiol Infect, 2015. **143**(8): p. 1585-93.
3. Aubry, M., et al., *Seroprevalence of arboviruses among blood donors in French Polynesia, 2011-2013.* Int J Infect Dis, 2015. **41**: p. 11-2.
4. Chisenga, C.C., et al., *Sero-prevalence of arthropod-borne viral infections among Lukanga swamp residents in Zambia.* PLoS One, 2020. **15**(7): p. e0235322.
5. Collenberg, E., et al., *Seroprevalence of six different viruses among pregnant women and blood donors in rural and urban Burkina Faso: A comparative analysis.* J Med Virol, 2006. **78**(5): p. 683-92.
6. Darcy, A., et al., *Solomon Islands dengue seroprevalence study--previous circulation of dengue confirmed.* P N G Med J, 2001. **44**(1-2): p. 43-7.

7. Ekong, P.S., et al., *A Retrospective Study of the Seroprevalence of Dengue Virus and Chikungunya Virus Exposures in Nigeria, 2010-2018.* Pathogens, 2022. **11**(7).
8. Faddy, H.M., et al., *Implications of dengue outbreaks for blood supply, Australia.* Emerg Infect Dis, 2013. **19**(5): p. 787-9.
9. Flichman, D.M., et al., *Epidemiology of Dengue in Argentina: Antibodies seroprevalence in blood donors and circulating serotypes.* J Clin Virol, 2022. **147**: p. 105078.
10. Gao, Z., et al., *Dengue virus infections among blood donors in Guangxi of China, 2013-2014.* Transfus Med, 2018. **28**(3): p. 236-242.
11. Hesse, E.M., et al., *Dengue Virus Exposures Among Deployed U.S. Military Personnel.* Am J Trop Med Hyg, 2017. **96**(5): p. 1222-1226.
12. Humphrey, J.M., et al., *Dengue and chikungunya seroprevalence among Qatari nationals and immigrants residing in Qatar.* PLoS One, 2019. **14**(1): p. e0211574.
13. Jain, A., S. Jain, and N. Chowdhury, *Seroprevalence of dengue in blood donors in an outbreak: experience of a blood bank in north India.* Trop Doct, 2019. **49**(3): p. 212-215.
14. Johnson, B.W., et al., *West Nile virus infection and serologic response among persons previously vaccinated against yellow fever and Japanese encephalitis viruses.* Vector Borne Zoonotic Dis, 2005. **5**(2): p. 137-45.
15. Khan, E., et al., *Co-circulations of two genotypes of dengue virus in 2006 out-break of dengue hemorrhagic fever in Karachi, Pakistan.* J Clin Virol, 2008. **43**(2): p. 176-9.
16. Kwan, T.H., et al., *Assessing the risk of dengue virus transmission in a non-endemic city surrounded by endemic and hyperendemic areas.* Int J Infect Dis, 2017. **55**: p. 99-101.
17. Larrieu, S., et al., *Dengue outbreaks: a constant risk for Reunion Island. Results from a seroprevalence study among blood donors.* Trans R Soc Trop Med Hyg, 2014. **108**(1): p. 57-9.
18. Lee, Y.H., et al., *Retrospective Seroepidemiology study of dengue virus infection in Taiwan.* BMC Infect Dis, 2021. **21**(1): p. 96.
19. Li, L., et al., *Epidemiological survey and screening strategy for dengue virus in blood donors from Yunnan Province.* BMC Infect Dis, 2021. **21**(1): p. 104.
20. Lo, C.L., S.P. Yip, and P.H. Leung, *Seroprevalence of dengue in the general population of Hong Kong.* Trop Med Int Health, 2013. **18**(9): p. 1097-1102.
21. Low, S.L., et al., *Dengue seroprevalence of healthy adults in Singapore: serosurvey among blood donors, 2009.* Am J Trop Med Hyg, 2015. **93**(1): p. 40-45.
22. Mohammed, H., et al., *Prevalence of anti-dengue immunoglobulin G antibodies among American Red Cross blood donors in Puerto Rico, 2006.* Transfusion, 2012. **52**(8): p. 1652-6.
23. Ochieng, C., et al., *Seroprevalence of Infections with Dengue, Rift Valley Fever and Chikungunya Viruses in Kenya, 2007.* PLoS One, 2015. **10**(7): p. e0132645.
24. Overbosch, F.W., et al., *High prevalence of previous dengue virus infection among first-generation Surinamese immigrants in the Netherlands.* BMC Infect Dis, 2014. **14**(1): p. 493.
25. Pollett, S., et al., *The seroepidemiology of dengue in a US military population based in Puerto Rico during the early phase of the Zika pandemic.* PLoS Negl Trop Dis, 2022. **16**(1): p. e0009986.
26. Shauri, H.S., et al., *Seroprevalence of Dengue and Chikungunya antibodies among blood donors in Dar es Salaam and Zanzibar, Tanzania: a cross-sectional study.* BMC Infect Dis, 2021. **21**(1): p. 911.
27. Slavov, S.N., et al., *Dengue RNA detection and seroprevalence in blood donors during an outbreak in the Sao Paulo State, Brazil, 2016.* J Med Virol, 2021. **93**(6): p. 3344-3349.
28. Sule, W.F., et al., *Probable primary and secondary dengue viral infections and associated host factors among university undergraduates in Osun State, Nigeria.* Alexandria Journal of Medicine, 2019. **55**(1): p. 25-30.
29. Tan, L.K., et al., *Force of Infection and True Infection Rate of Dengue in Singapore: Implications for Dengue Control and Management.* Am J Epidemiol, 2019. **188**(8): p. 1529-1538.

30. Tinto, B., et al., *Serological Evidence of Zika Virus Circulation in Burkina Faso.* Pathogens, 2022. **11**(7).
31. Yew, Y.W., et al., *Seroepidemiology of dengue virus infection among adults in Singapore.* Annals of the Academy of Medicine, Singapore, 2009. **38**(8): p. 667-75.
32. Ang, L.W., et al., *Seroprevalence of IgG antibodies against diphtheria antitoxin among migrant workers in Singapore, 2016-2019.* BMC Public Health, 2022. **22**(1): p. 111.
33. Ang, L.W., L. James, and K.T. Goh, *Prevalence of diphtheria and tetanus antibodies among adults in Singapore: a national serological study to identify most susceptible population groups.* J Public Health (Oxf), 2016. **38**(1): p. 99-105.
34. Aue, A., et al., *Immunity against diphtheria and tetanus in German blood donors.* Med Microbiol Immunol, 2003. **192**(2): p. 93-7.
35. Comodo, N., et al., *Low prevalence of diphtheria immunity in the population of Florence, Italy.* Eur J Epidemiol, 1996. **12**(3): p. 251-5.
36. Edmunds, W.J., et al., *The sero-epidemiology of diphtheria in Western Europe. ESEN Project. European Sero-Epidemiology Network.* Epidemiol Infect, 2000. **125**(1): p. 113-25.
37. Gupta, R.K., et al., *Diphtheria antitoxin levels in US blood and plasma donors.* J Infect Dis, 1996. **173**(6): p. 1493-7.
38. Heijstek, M.W., et al., *Differences in persistence of measles, mumps, rubella, diphtheria and tetanus antibodies between children with rheumatic disease and healthy controls: a retrospective cross-sectional study.* Ann Rheum Dis, 2012. **71**(6): p. 948-54.
39. Lai, F.Y., et al., *Comparative seroepidemiology of pertussis, diphtheria and poliovirus antibodies in Singapore: waning pertussis immunity in a highly immunized population and the need for adolescent booster doses.* Vaccine, 2012. **30**(24): p. 3566-71.
40. Maksimova, N.M., et al., *Results of the next scheduled revaccination against diphtheria in the adult population in 2014-2016 according to serological testing data.* Profilakticheskaya meditsina, 2021. **24**(9): p. 38-38.
41. Maple, P.A., et al., *Immunity to diphtheria and tetanus in England and Wales.* Vaccine, 2000. **19**(2-3): p. 167-73.
42. Mathei, C., et al., *Diphtheria immunity in Flanders.* Eur J Clin Microbiol Infect Dis, 1997. **16**(9): p. 631-6.
43. Murhekar, M.V., et al., *Immunity against diphtheria among children aged 5-17 years in India, 2017-18: a cross-sectional, population-based serosurvey.* Lancet Infect Dis, 2021. **21**(6): p. 868-875.
44. Ng, Y., et al., *Seroprevalence of vaccine-preventable diseases among children and adolescents in Singapore: Results from the National Paediatric Seroprevalence Survey 2018.* Int J Infect Dis, 2020. **92**: p. 234-240.
45. Osborne, K., et al., *Ten years of serological surveillance in England and Wales: methods, results, implications and action.* Int J Epidemiol, 2000. **29**(2): p. 362-8.
46. Pelletier, L., et al., *Immunity to diphtheria in a sample of the Canadian adult population.* Can J Infect Dis, 1998. **9**(6): p. 367-71.
47. Pimenta, F.P., et al., *Diphtheria-neutralizing antibody levels in healthy adults from Rio de Janeiro, Brazil.* Mem Inst Oswaldo Cruz, 2006. **101**(4): p. 459-62.
48. Schwarz, T.F., et al., *Hepatitis C and arboviral antibodies in the island populations of Mauritius and Rodrigues.* J Med Virol, 1994. **44**(4): p. 379-83.
49. Staehelin, C., et al., *Seroprotection rates of vaccine-preventable diseases among newly arrived Eritrean asylum seekers in Switzerland: a cross-sectional study.* J Travel Med, 2019. **26**(6).
50. Wagner, K.S., et al., *Immunity to tetanus and diphtheria in the UK in 2009.* Vaccine, 2012. **30**(49): p. 7111-7.
51. Arslan, M., et al., *Hepatitis A antibodies in liver transplant recipients: evidence for loss of immunity posttransplantation.* Liver Transpl, 2000. **6**(2): p. 191-5.

52. Bassal, R., et al., *Seroprevalence of Hepatitis A Twelve Years After the Implementation of Toddlers' Vaccination: A Population-Based Study in Israel.* Pediatr Infect Dis J, 2017. **36**(10): p. e248-e251.
53. Binotto, E., et al., *A serological re-evaluation of acute non-A non-B hepatitis from the early 1970s.* Aust N Z J Med, 2000. **30**(6): p. 668-74.
54. Bodner, C., et al., *Childhood exposure to infection and risk of adult onset wheeze and atopy.* Thorax, 2000. **55**(5): p. 383-7.
55. Chin, K.P., et al., *Current seroepidemiology of hepatitis A in Hong Kong.* J Med Virol, 1991. **34**(3): p. 191-3.
56. Ciencewicki, J.M., et al., *Plasma Donors in the Southwestern United States Positively Contribute to the Diverse Therapeutic Antibody Profile of Immune Globulin Products.* Sci Rep, 2020. **10**(1): p. 6850.
57. Crum-Cianflone, N.F., et al., *Long-term durability of immune responses after hepatitis A vaccination among HIV-infected adults.* J Infect Dis, 2011. **203**(12): p. 1815-23.
58. da Silva, E.F., et al., *HAV and HBV seroprevalence in 1,000 patients with chronic HCV infection in a Tertiary Care Center in Sao Paulo, Brazil.* Ann Hepatol, 2016. **15**(5): p. 691-5.
59. Dalton, H.R., et al., *Autochthonous hepatitis E in Southwest England: a comparison with hepatitis A.* Eur J Clin Microbiol Infect Dis, 2008. **27**(7): p. 579-85.
60. de Almeida, E.A.D.C., et al., *Declining prevalence of hepatitis A and silent circulation of hepatitis E virus infection in southeastern Brazil.* Int J Infect Dis, 2020. **101**: p. 17-23.
61. de Ory, F., et al., *Is there a change in cytomegalovirus seroepidemiology in Spain?* Eur J Epidemiol, 2004. **19**(1): p. 85-9.
62. Engle, R.E., et al., *Transfusion-associated hepatitis before the screening of blood for hepatitis risk factors.* Transfusion, 2014. **54**(11): p. 2833-41.
63. Godoi, E.R., et al., *Loss of hepatitis A virus antibodies after bone marrow transplantation.* Bone Marrow Transplant, 2006. **38**(1): p. 37-40.
64. Hennessey, K.A., et al., *Hepatitis A seroprevalence and risk factors among homeless adults in San Francisco: should homelessness be included in the risk-based strategy for vaccination?* Public Health Rep, 2009. **124**(6): p. 813-7.
65. Heywood, A.E., et al., *Changes in seroprevalence to hepatitis A in Victoria, Australia: a comparison of three time points.* Vaccine, 2012. **30**(42): p. 6020-6.
66. Krumbholz, A., et al., *Prevalence of antibodies against hepatitis A virus among children and adolescents in Germany.* Med Microbiol Immunol, 2013. **202**(6): p. 417-24.
67. Kurkela, S., et al., *Comparative hepatitis A seroepidemiology in 10 European countries.* Epidemiol Infect, 2012. **140**(12): p. 2172-81.
68. Lee, H., et al., *Seroepidemiology of hepatitis A in Korea: changes over the past 30 years.* J Korean Med Sci, 2011. **26**(6): p. 791-6.
69. Lopes, T., et al., *Racial differences in seroprevalence of HAV and HEV in blood donors in the Western Cape, South Africa: a clue to the predominant HEV genotype?* Epidemiol Infect, 2017. **145**(9): p. 1910-1912.
70. Louati, N., et al., *Comparison of hepatitis A seroprevalence in blood donors in South Tunisia between 2000 and 2007.* Archives de l'Institut Pasteur de Tunis, 2009. **86**(1-4): p. 69-74.
71. Martin, D.J., et al., *The current epidemiology of hepatitis A infection in South Africa: implications for vaccination.* Trans R Soc Trop Med Hyg, 1994. **88**(3): p. 288-91.
72. Medic, S., et al., *Declining seroprevalence of hepatitis A in Vojvodina, Serbia.* PLoS One, 2019. **14**(6): p. e0217176.
73. Morris, M.C., et al., *The changing epidemiological pattern of hepatitis A in England and Wales.* Epidemiol Infect, 2002. **128**(3): p. 457-63.
74. Mosley, J.W., et al., *Hepatitis A virus transmission by blood products in the United States. Transfusion Safety Study Group.* Vox Sang, 1994. **67 Suppl 1**(SUPPL. 1): p. 24-8.

75. Nevin, R.L. and D.W. Niebuhr, *Rising hepatitis A immunity in U.S. military recruits.* Mil Med, 2007. **172**(7): p. 787-93.
76. Pramoolsinsap, C., et al., *Susceptibility to hepatitis A virus infection among chronic liver disease patients and healthy blood donors in Thailand.* The Southeast Asian journal of tropical medicine and public health, 1999. **30**(1): p. 91-5.
77. Queirós, L., et al., *[The seroprevalence for hepatitis E viral antibodies in the northern region of Portugal (among the donor population)].* Acta medica portuguesa, 1997. **10**(6-7): p. 447-53.
78. Schoch, S., et al., *Hepatitis A Virus Incidence Rates and Biomarker Dynamics for Plasma Donors, United States.* Emerg Infect Dis, 2021. **27**(11): p. 2718-2824.
79. Schwarz, T.F., et al., *Prevalence of antibodies against hepatitis A virus, hepatitis B virus, and Treponema pallidum in Mauritius.* Scand J Infect Dis, 1991. **23**(5): p. 535-41.
80. Shinohara, N., et al., *Hepatitis A virus and hepatitis E virus prevalence relates to human immunodeficiency virus infection in Japanese male blood donors.* Microbiol Immunol, 2020. **64**(5): p. 392-395.
81. Sun, P., et al., *Prevalence of hepatitis A viral RNA and antibodies among Chinese blood donors.* Genetics and Molecular Research, 2015. **14**(4): p. 16431-16437.
82. Thomas, D.L., et al., *Seroreactivity to hepatitis E virus in areas where the disease is not endemic.* J Clin Microbiol, 1997. **35**(5): p. 1244-7.
83. Traore, K.A., et al., *Seroprevalence of fecal-oral transmitted hepatitis A and E virus antibodies in Burkina Faso.* PLoS One, 2012. **7**(10): p. e48125.
84. Trinta, K.S., et al., *Hepatitis E virus infection in selected Brazilian populations.* Mem Inst Oswaldo Cruz, 2001. **96**(1): p. 25-9.
85. Vilibic-Cavlek, T., et al., *Seroepidemiology of hepatitis a in the croatian population.* Hepat Mon, 2011. **11**(12): p. 997-9.
86. Yamamoto, C., et al., *Very low prevalence of anti-HAV in Japan: high potential for future outbreak.* Sci Rep, 2019. **9**(1): p. 1493.
87. Yan, B.Y., et al., *Changes in seroprevalence of hepatitis A after the implementation of universal childhood vaccination in Shandong Province, China: A comparison between 2006 and 2014.* Int J Infect Dis, 2019. **82**: p. 129-134.
88. Young, M.K., et al., *Hepatitis A virus antibodies in Australian blood donors: implications for immunoglobulin sufficiency.* Vaccine, 2015. **33**(39): p. 5135-9.
89. Ang, L.W., et al., *Seroepidemiology of hepatitis B virus infection among adults in Singapore: a 12-year review.* Vaccine, 2013. **32**(1): p. 103-10.
90. Ang, L.W., et al., *Seroprevalence of hepatitis B virus infection among children and adolescents in Singapore, 2008-2010.* J Med Virol, 2013. **85**(4): p. 583-8.
91. Attaran, M.S., et al., *Serological and molecular characterization of hepatitis B virus in asymptomatic blood donors in Iran.* Iran J Microbiol, 2018. **10**(1): p. 59-64.
92. Bae, E., et al., *Prevalence and clinical significance of occult hepatitis B virus infection among renal transplant recipients in Korea.* Scand J Infect Dis, 2012. **44**(10): p. 788-92.
93. Bassig, B.A., et al., *Serologic markers of viral infection and risk of non-Hodgkin lymphoma: A pooled study of three prospective cohorts in China and Singapore.* Int J Cancer, 2018. **143**(3): p. 570-579.
94. Boccalini, S., et al., *Sero-epidemiology of hepatitis B markers in the population of Tuscany, Central Italy, 20 years after the implementation of universal vaccination.* Hum Vaccin Immunother, 2013. **9**(3): p. 636-41.
95. Brown, A.E., et al., *Prevalence of markers for HIV, hepatitis B and hepatitis C infection in UK military recruits.* Epidemiol Infect, 2011. **139**(8): p. 1166-71.
96. Chen, C.L., et al., *Slow decline of hepatitis B burden in general population: Results from a population-based survey and longitudinal follow-up study in Taiwan.* J Hepatol, 2015. **63**(2): p. 354-63.

97. Cowie, B., et al., *Markers of hepatitis B virus infection and immunity in Victoria, Australia, 1995 to 2005.* Aust N Z J Public Health, 2010. **34**(1): p. 72-8.
98. Dassah, S., et al., *Seroconversion of Hepatitis B Vaccine in Young Children in the Kassena Nankana District of Ghana: A Cross-Sectional Study.* PLoS One, 2015. **10**(12): p. e0145209.
99. Deveci, U. and U. Acar, *Seroprevalence of Hepatitis B Virus, Hepatitis C Virus and Human Immunodeficiency Virus in Children Undergoing Endoscopy in Our Pediatric Gastroenterology Clinic.* Journal of Pediatric Infection, 2020. **14**(1): p. 5-8.
100. Diop-Ndiaye, H., et al., *Hepatitis B, C seroprevalence and delta viruses in HIV-1 Senegalese patients at HAART initiation (retrospective study).* J Med Virol, 2008. **80**(8): p. 1332-6.
101. Fiscus, S.A., et al., *Hepatitis C virus seroprevalence in clients of sexually transmitted disease clinics in North Carolina.* Sex Transm Dis, 1994. **21**(3): p. 155-60.
102. Gilson, R.J., et al., *Hepatitis B virus infection in patients attending a genitourinary medicine clinic: risk factors and vaccine coverage.* Sex Transm Infect, 1998. **74**(2): p. 110-5.
103. Hennessey, K.A., et al., *Prevalence of infection with hepatitis B and C viruses and co-infection with HIV in three jails: a case for viral hepatitis prevention in jails in the United States.* J Urban Health, 2009. **86**(1): p. 93-105.
104. Hudu, S.A., et al., *Molecular and serological detection of occult hepatitis B virus among healthy hepatitis B surface antigen-negative blood donors in Malaysia.* Afr Health Sci, 2016. **16**(3): p. 677-683.
105. Jilg, W., et al., *Prevalence of markers of hepatitis B in the adult German population.* J Med Virol, 2001. **63**(2): p. 96-102.
106. Kang, M., et al., *Virologic and serologic outcomes of mono versus dual HBV therapy and characterization of HIV/HBV coinfection in a US cohort.* J Acquir Immune Defic Syndr, 2014. **66**(2): p. 172-80.
107. Katoonizadeh, A., et al., *Intra-familial Transmission of Chronic Hepatitis B Infection: A Large Population-Based Cohort Study in Northern Iran.* Arch Iran Med, 2018. **21**(10): p. 436-442.
108. Kelly, H., et al., *A random cluster survey and a convenience sample give comparable estimates of immunity to vaccine preventable diseases in children of school age in Victoria, Australia.* Vaccine, 2002. **20**(25-26): p. 3130-6.
109. Khamduang, W., et al., *Serologic characteristics of hepatitis B virus among hill-tribe children in Omkoi district, Chiangmai province, Thailand.* J Infect Dev Ctries, 2019. **13**(2): p. 169-173.
110. Khetsuriani, N., et al., *Substantial decline in hepatitis B virus infections following vaccine introduction in Tajikistan.* Vaccine, 2015. **33**(32): p. 4019-24.
111. Kurien, T., et al., *Community prevalence of hepatitis B infection and modes of transmission in Tamil Nadu, India.* Indian J Med Res, 2005. **121**(5): p. 670-5.
112. Li, H., et al., *Incident hepatitis B virus infection and immunisation uptake in Australian prison inmates.* Vaccine, 2020. **38**(16): p. 3255-3260.
113. Lukhwareni, A., et al., *Increased detection of HBV DNA in HBsAg-positive and HBsAg-negative South African HIV/AIDS patients enrolling for highly active antiretroviral therapy at a Tertiary Hospital.* J Med Virol, 2009. **81**(3): p. 406-12.
114. Mitsui, T., et al., *Prevalence of hepatitis E virus infection among hemodialysis patients in Japan: evidence for infection with a genotype 3 HEV by blood transfusion.* J Med Virol, 2004. **74**(4): p. 563-72.
115. Murrill, C.S., et al., *Age-specific seroprevalence of HIV, hepatitis B virus, and hepatitis C virus infection among injection drug users admitted to drug treatment in 6 US cities.* Am J Public Health, 2002. **92**(3): p. 385-7.
116. Nardone, A., et al., *A comparison of hepatitis B seroepidemiology in ten European countries.* Epidemiol Infect, 2009. **137**(7): p. 961-9.
117. Pataccini, G., et al., *First molecular epidemiological study of hepatitis B and D in individuals infected with human T-lymphotropic virus 1/2 from Argentina.* J Med Virol, 2021. **93**(6): p. 3995-3998.

118. Phinius, B.B., et al., *Incidence of hepatitis B virus infection among human immunodeficiency virus-infected treatment naive adults in Botswana.* Medicine (Baltimore), 2020. **99**(9): p. e19341.
119. Prabdial-Sing, N., et al., *Hepatitis B sero-prevalence in children under 15 years of age in South Africa using residual samples from community-based febrile rash surveillance.* PLoS One, 2019. **14**(5): p. e0217415.
120. Price, H., et al., *Hepatitis B serological markers and plasma DNA concentrations.* AIDS, 2017. **31**(8): p. 1109-1117.
121. Roche, R., et al., *Prevalence of hepatitis B immunity and infection in home self-sampling HIV service users.* Sex Transm Infect, 2022. **98**(4): p. 286-292.
122. Rollag, H., et al., *Serological markers of hepatitis B virus and cytomegalovirus infections in Norwegians with coagulation factor defects.* Blut, 1990. **60**(2): p. 93-6.
123. Roy, K.M., et al., *Vaccination induced immunity to the hepatitis B virus among high-risk groups in Glasgow 1993-2001: evaluating the effectiveness of the United Kingdom's selective immunisation policy.* Scott Med J, 2008. **53**(4): p. 13-7.
124. Scott, P.T., et al., *Hepatitis B immunity in United States military recruits.* J Infect Dis, 2005. **191**(11): p. 1835-41.
125. Theeten, H., et al., *Universal hepatitis B vaccination in Belgium: impact on serological markers 3 and 7 years after implementation.* Epidemiol Infect, 2014. **142**(2): p. 251-61.
126. Thompson, S.C., et al., *Exposure to hepatitis B and C of tattooists in Victoria in 1984.* J Viral Hepat, 1997. **4**(2): p. 135-8.
127. Thomson, J.A., et al., *The prevalence of hepatitis C in patients admitted with acute hepatitis to Fairfield Infectious Diseases Hospital, 1971-1975.* Med J Aust, 1998. **169**(7): p. 360-3.
128. Tohme, R.A., et al., *Hepatitis B virus infection among pregnant women in Haiti: A cross-sectional serosurvey.* J Clin Virol, 2016. **76**: p. 66-71.
129. Triki, H., et al., *Seroepidemiology of hepatitis B, C and delta viruses in Tunisia.* Trans R Soc Trop Med Hyg, 1997. **91**(1): p. 11-4.
130. van Houdt, R., et al., *Unexpectedly high proportion of drug users and men having sex with men who develop chronic hepatitis B infection.* J Hepatol, 2012. **57**(3): p. 529-33.
131. Vasconcelos, H.C., et al., *Hepatitis B and C prevalences among blood donors in the south region of Brazil.* Mem Inst Oswaldo Cruz, 1994. **89**(4): p. 503-7.
132. Wahome, E., et al., *Hepatitis B Virus Incidence and Risk Factors Among Human Immunodeficiency Virus-1 Negative Men Who Have Sex With Men in Kenya.* Open Forum Infect Dis, 2017. **4**(1): p. ofw253.
133. Zaaijer, H.L., et al., *Concurrence of hepatitis B surface antibodies and surface antigen: implications for postvaccination control of health care workers.* J Viral Hepat, 2002. **9**(2): p. 146-8.
134. Zhang, J., et al., *Prevalence of Hepatitis B Virus Infection in Kaifeng,China: A 5-year Observation.* Microbiology and Biotechnology Letters, 2018. **46**(4): p. 430-433.
135. Zubieta, A.M., et al., *[Seroprevalence of hepatitis B virus in children with cancer under chemotherapy in 6 hospitals of Santiago, Chile].* Rev Med Chil, 2009. **137**(7): p. 906-11.
136. Ahmed, A.M.A., et al., *The seropervelance of infectious hepatitis viruses (HBV, HCV and HEV) among blood donors and their correlation to risk factors in Qena governorate, Upper Egypt.* Virusdisease, 2020. **31**(3): p. 292-298.
137. Al Absi, E.S., et al., *The prevalence of HEV among non-A-C hepatitis in Qatar and efficiency of serological markers for the diagnosis of hepatitis E.* BMC Gastroenterol, 2021. **21**(1): p. 266.
138. Al-Oebady, M.A.H., *Immunological and Molecular Detection of Hepatitis E Virus in AL-Samawah City, Iraq.* Indian Journal of Public Health Research & Development, 2019. **10**(6): p. 812-812.
139. Bernal, M.C., et al., *Seroepidemiological study of hepatitis E virus in different population groups.* Eur J Clin Microbiol Infect Dis, 1995. **14**(11): p. 954-8.

140. Boutrouille, A., et al., *Prevalence of anti-hepatitis E virus antibodies in French blood donors.* J Clin Microbiol, 2007. **45**(6): p. 2009-10.
141. Bouwknegt, M., et al., *Bayesian estimation of hepatitis E virus seroprevalence for populations with different exposure levels to swine in The Netherlands.* Epidemiol Infect, 2008. **136**(4): p. 567-76.
142. Capai, L., et al., *Seroprevalence of hepatitis E virus among blood donors on Corsica, France, 2017.* Euro Surveill, 2020. **25**(5).
143. Chen, X., et al., *Identification of hepatitis E virus subtype 4f in blood donors in Shanghai, China.* Virus Res, 2019. **265**: p. 30-33.
144. Christensen, P.B., et al., *Time trend of the prevalence of hepatitis E antibodies among farmers and blood donors: a potential zoonosis in Denmark.* Clin Infect Dis, 2008. **47**(8): p. 1026-31.
145. Cleland, A., et al., *Hepatitis E virus in Scottish blood donors.* Vox Sang, 2013. **105**(4): p. 283-9.
146. Covarrubias, N., et al., *[Hepatitis E virus seroprevalence in blood donors in a university hospital in Chile].* Rev Chilena Infectol, 2018. **35**(4): p. 455-457.
147. Dalton, H.R., et al., *Hepatitis E in new zealand.* J Gastroenterol Hepatol, 2007. **22**(8): p. 1236-40.
148. Dalton, H.R., et al., *Autochthonous hepatitis E in Southwest England: natural history, complications and seasonal variation, and hepatitis E virus IgG seroprevalence in blood donors, the elderly and patients with chronic liver disease.* Eur J Gastroenterol Hepatol, 2008. **20**(8): p. 784-90.
149. Ehteram, H., et al., *Seroprevalence of Hepatitis E Virus infection among volunteer blood donors in central province of Iran in 2012.* Iranian journal of microbiology, 2013. **5**(2): p. 172-6.
150. Fix, A.D., et al., *Prevalence of antibodies to hepatitis E in two rural Egyptian communities.* Am J Trop Med Hyg, 2000. **62**(4): p. 519-23.
151. Fontana, R.J., et al., *Role of Hepatitis E Virus Infection in North American Patients With Severe Acute Liver Injury.* Clin Transl Gastroenterol, 2020. **11**(11): p. e00273.
152. Fritz-Weltin, M., et al., *Hepatitis E virus as a trigger for Guillain-Barre syndrome.* BMC Neurol, 2021. **21**(1): p. 304.
153. Fu, P., et al., *Hepatitis E virus prevalence among blood donors in Dali, China.* Virol J, 2021. **18**(1): p. 141.
154. Gerard, L., et al., *Hepatitis E infection in adults with primary immunodeficiency with or without immunoglobulin replacement therapy.* Blood Transfus, 2022. **20**(6): p. 516-524.
155. Gerardin, P., et al., *Low seroprevalence of hepatitis E on Reunion island.* One Health, 2019. **8**: p. 100110.
156. Goncales, N.S., et al., *Hepatitis E virus immunoglobulin G antibodies in different populations in Campinas, Brazil.* Clin Diagn Lab Immunol, 2000. **7**(5): p. 813-6.
157. Grabarczyk, P., et al., *Molecular and serological infection marker screening in blood donors indicates high endemicity of hepatitis E virus in Poland.* Transfusion, 2018. **58**(5): p. 1245-1253.
158. Guo, Q.S., et al., *Prevalence of hepatitis E virus in Chinese blood donors.* J Clin Microbiol, 2010. **48**(1): p. 317-8.
159. Haagsma, E.B., et al., *Prevalence of hepatitis E virus infection in liver transplant recipients.* Liver Transpl, 2009. **15**(10): p. 1225-8.
160. Hardtke, S., et al., *Risk factors and seroprevalence of hepatitis E evaluated in frozen-serum samples (2002-2003) of pregnant women compared with female blood donors in a Southern region of Brazil.* J Med Virol, 2018. **90**(12): p. 1856-1862.
161. Harritshoj, L.H., et al., *Epidemiology of hepatitis E virus infection in a cohort of 4023 immunocompromised patients.* Int J Infect Dis, 2020. **91**: p. 188-195.
162. Heil, J., et al., *Hepatitis E prevalence in a sexual high-risk population compared to the general population.* PLoS One, 2018. **13**(1): p. e0191798.
163. Herremans, M., et al., *Swine-like hepatitis E viruses are a cause of unexplained hepatitis in the Netherlands.* J Viral Hepat, 2007. **14**(2): p. 140-6.

164. Hewitt, J., et al., *Prevalence of hepatitis E virus antibodies and infection in New Zealand blood donors.* N Z Med J, 2018. **131**(1469): p. 38-43.
165. Hewitt, P.E., et al., *Hepatitis E virus in blood components: a prevalence and transmission study in southeast England.* Lancet, 2014. **384**(9956): p. 1766-73.
166. Ho, E., et al., *Stable HEV IgG seroprevalence in Belgium between 2006 and 2014.* J Viral Hepat, 2020. **27**(11): p. 1253-1260.
167. Hoffmann, P., et al., *Hepatitis E seroprevalence in a German cohort of patients with inflammatory bowel diseases.* PLoS One, 2020. **15**(10): p. e0239825.
168. Hogema, B.M., et al., *Past and present of hepatitis E in the Netherlands.* Transfusion, 2014. **54**(12): p. 3092-6.
169. Ijaz, S., et al., *Indigenous hepatitis E virus infection in England: more common than it seems.* J Clin Virol, 2009. **44**(4): p. 272-6.
170. Izopet, J., et al., *Hepatitis E IgG seroprevalence in three hyperendemic areas: Nepal, Bangladesh and southwest France.* J. Viral Hepatitis, 2015. **22**((Izopet J.; Sauné K.; Kamar N.) Université Toulouse III Paul Sabatier, Toulouse, France): p. 113-114.
171. Juhl, D., et al., *Seroprevalence and incidence of hepatitis E virus infection in German blood donors.* Transfusion, 2014. **54**(1): p. 49-56.
172. Jupattanasin, S., et al., *A Nationwide Survey of the Seroprevalence of Hepatitis E Virus Infections Among Blood Donors in Thailand.* Viral Immunol, 2019. **32**(7): p. 302-307.
173. Karetnyi, Y.V., M.J. Gilchrist, and S.J. Naides, *Hepatitis E virus infection prevalence among selected populations in Iowa.* J Clin Virol, 1999. **14**(1): p. 51-5.
174. Katiyar, H., et al., *Prevalence of hepatitis E virus viremia and antibodies among healthy blood donors in India.* Indian J Gastroenterol, 2018. **37**(4): p. 342-346.
175. Kaufmann, A., et al., *Hepatitis E virus seroprevalence among blood donors in southwest Switzerland.* PLoS One, 2011. **6**(6): p. e21150.
176. Khuroo, M.S., S. Kamili, and G.N. Yattoo, *Hepatitis E virus infection may be transmitted through blood transfusions in an endemic area.* J Gastroenterol Hepatol, 2004. **19**(7): p. 778-84.
177. Kiesslich, D., J.E. Rocha junior, and M.A. Crispim, *Prevalence of hepatitis E virus antibodies among different groups in the Amazonian basin.* Trans R Soc Trop Med Hyg, 2002. **96**(2): p. 215.
178. Korsman, S., D. Hardie, and M. Kaba, *Hepatitis E virus in patients with acute hepatitis in Cape Town, South Africa, 2011.* S Afr Med J, 2019. **109**(8): p. 582-583.
179. Lemos, G., et al., *Hepatitis E virus in Cuba.* J Clin Virol, 2000. **16**(1): p. 71-5.
180. Mateos, M.L., et al., *Hepatitis E virus: relevance in blood donors and other risk groups.* Vox Sang, 1998. **75**(4): p. 267-9.
181. Mesquita, J.R., et al., *Evidence of autochthonous hepatitis E in a Portuguese pediatric cohort, 1992-1995.* J Med Virol, 2016. **88**(5): p. 919-21.
182. Minagi, T., et al., *Hepatitis E virus in donor plasma collected in Japan.* Vox Sang, 2016. **111**(3): p. 242-246.
183. Minuk, G.Y., et al., *Serological evidence of hepatitis E virus infection in an indigenous North American population.* Can J Gastroenterol, 2007. **21**(7): p. 439-42.
184. Moaven, L., et al., *Seroepidemiology of hepatitis E in selected Australian populations.* J Med Virol, 1995. **45**(3): p. 326-30.
185. Mooij, S.H., et al., *Risk factors for hepatitis E virus seropositivity in Dutch blood donors.* BMC Infect Dis, 2018. **18**(1): p. 173.
186. Moss da Silva, C., et al., *Detection and characterization of hepatitis E virus genotype 3 in HIV-infected patients and blood donors from southern Brazil.* Int J Infect Dis, 2019. **86**: p. 114-121.
187. Nasrallah, G.K., et al., *Seroprevalence of hepatitis E virus among blood donors in Qatar (2013-2016).* Transfusion, 2017. **57**(7): p. 1801-1807.
188. Niederhauser, C., et al., *Current hepatitis E virus seroprevalence in Swiss blood donors and apparent decline from 1997 to 2016.* Euro Surveill, 2018. **23**(35).

189. O'Riordan, J., et al., *Hepatitis E virus infection in the Irish blood donor population.* Transfusion, 2016. **56**(11): p. 2868-2876.
190. Ogut, S., et al., *Hepatitis E Infection in Solid Organ Transplant Recipients in Turkey.* Turk J Gastroenterol, 2022. **33**(1): p. 68-73.
191. Olsoy, I.B., et al., *Seroprevalence of hepatitis E virus (HEV) in a general adult population in Northern Norway: the Tromso study.* Med Microbiol Immunol, 2019. **208**(6): p. 715-725.
192. Parana, R., et al., *Prevalence of hepatitis E virus IgG antibodies in patients from a referral unit of liver diseases in Salvador, Bahia, Brazil.* Am J Trop Med Hyg, 1997. **57**(1): p. 60-1.
193. Parsa, R., et al., *Detection of Hepatitis E Virus Genotype 1 Among Blood Donors From Southwest of Iran.* Hepat Mon, 2016. **16**(6): p. e34202.
194. Passos-Castilho, A.M., et al., *High prevalence of hepatitis E virus antibodies in Sao Paulo, Southeastern Brazil: analysis of a group of blood donors representative of the general population.* Braz J Infect Dis, 2017. **21**(5): p. 535-539.
195. Petrovic, T., et al., *Prevalence of hepatitis E virus (HEV) antibodies in Serbian blood donors.* J Infect Dev Ctries, 2014. **8**(10): p. 1322-7.
196. Pisano, M.B., et al., *Hepatitis E virus in blood donors from Argentina: A possible source of viral infection?* Travel Med Infect Dis, 2022. **48**: p. 102355.
197. Puttini, C., et al., *Seroprevalence of hepatitis E virus (HEV) infection in blood donors and renal transplant recipients: a retrospective study from central Italy.* Infez Med, 2015. **23**(3): p. 253-6.
198. Reinheimer, C., R. Allwinn, and A. Berger, *Hepatitis E: are psychiatric patients on special risk?* Med Microbiol Immunol, 2012. **201**(2): p. 171-5.
199. Ren, F., et al., *Hepatitis E virus seroprevalence and molecular study among blood donors in China.* Transfusion, 2014. **54**(3 Pt 2): p. 910-7.
200. Schemmerer, M., et al., *Time course of hepatitis E-specific antibodies in adults.* J Viral Hepat, 2017. **24**(1): p. 75-79.
201. Seow, H.F., et al., *Seroprevalence of antibodies to hepatitis E virus in the normal blood donor population and two aboriginal communities in Malaysia.* J Med Virol, 1999. **59**(2): p. 164-8.
202. Sepanlou, S., et al., *A Population-based Seroepidemiological Study on Hepatitis E Virus in Iran.* Middle East J Dig Dis, 2010. **2**(2): p. 97-103.
203. Servant-Delmas, A., et al., *New insights into the natural history of hepatitis E virus infection through a longitudinal study of multitransfused immunocompetent patients in France.* J Viral Hepat, 2016. **23**(7): p. 569-75.
204. Slot, E., et al., *Silent hepatitis E virus infection in Dutch blood donors, 2011 to 2012.* Euro Surveill, 2013. **18**(31).
205. Souza, A.J.S., et al., *Serological and molecular retrospective analysis of hepatitis E suspected cases from the Eastern Brazilian Amazon 1993-2014.* Rev Soc Bras Med Trop, 2019. **52**: p. e20180465.
206. Spada, E., et al., *A nationwide retrospective study on prevalence of hepatitis E virus infection in Italian blood donors.* Blood Transfus, 2018. **16**(5): p. 413-421.
207. Spreafico, M., et al., *Prevalence and 9-year incidence of hepatitis E virus infection among North Italian blood donors: Estimated transfusion risk.* J Viral Hepat, 2020. **27**(8): p. 858-861.
208. Stramer, S.L., et al., *Hepatitis E virus: seroprevalence and frequency of viral RNA detection among US blood donors.* Transfusion, 2016. **56**(2): p. 481-8.
209. Takeda, H., et al., *A nationwide survey for prevalence of hepatitis E virus antibody in qualified blood donors in Japan.* Vox Sang, 2010. **99**(4): p. 307-13.
210. Taremi, M., et al., *Prevalence of antibodies to hepatitis E virus among male blood donors in Tabriz, Islamic Republic of Iran.* Eastern Mediterranean health journal = La revue de sante de la Mediterranee orientale = al-Majallah al-sihhiyah li-sharq al-mutawassit, 2007. **13**(1): p. 98-102.
211. Thom, K., et al., *Hepatitis E virus (HEV) in Scotland: evidence of recent increase in viral circulation in humans.* Euro Surveill, 2018. **23**(12).

212. Ticehurst, J.R., et al., *Probable transmission of hepatitis E virus (HEV) via transfusion in the United States.* Transfusion, 2019. **59**(3): p. 1024-1034.
213. Toyoda, H., et al., *Prevalence of hepatitis E virus IgG antibody in Japanese patients with hemophilia.* Intervirology, 2008. **51**(1): p. 21-5.
214. Tsoi, W.C., et al., *Hepatitis E virus infection in Hong Kong blood donors.* Vox Sang, 2020. **115**(1): p. 11-17.
215. Unzueta, A., et al., *Hepatitis E virus serum antibodies and RNA prevalence in patients evaluated for heart and kidney transplantation.* Ann Hepatol, 2016. **15**(1): p. 33-40.
216. Vercouter, A.S., et al., *Hepatitis E virus prevalence in Flemish blood donors.* J Viral Hepat, 2019. **26**(10): p. 1218-1223.
217. Wang, M., et al., *The association of elevated alanine aminotransferase levels with hepatitis E virus infections among blood donors in China.* Transfusion, 2017. **57**(2): p. 273-279.
218. Wenzel, J.J., et al., *Decline in hepatitis E virus antibody prevalence in southeastern Germany, 1996-2011.* Hepatology, 2014. **60**(4): p. 1180-6.
219. Wong, L.P., et al., *The Risk of Transfusion-Transmitted Hepatitis E Virus: Evidence from Seroprevalence Screening of Blood Donations.* Indian J Hematol Blood Transfus, 2022. **38**(1): p. 145-152.
220. Xu, C., et al., *An assessment of hepatitis E virus (HEV) in US blood donors and recipients: no detectable HEV RNA in 1939 donors tested and no evidence for HEV transmission to 362 prospectively followed recipients.* Transfusion, 2013. **53**(10 Pt 2): p. 2505-11.
221. Yasar, O., et al., *HEV seroprevalence in blood donors in Turkey by two commercial total anti-HEV Ab ELISA kits.* J Med Virol, 2019. **91**(12): p. 2174-2181.
222. Zorzetto, R., et al., *Unusual high prevalence of antibodies to hepatitis E virus in South Brazil.* FEMS Microbiol Lett, 2021. **368**(13): p. fnab076-fnab076.
223. Collins, S., et al., *Haemophilus influenzae type b (Hib) seroprevalence and current epidemiology in England and Wales.* J Infect, 2018. **76**(4): p. 335-341.
224. Hong, E., et al., *Haemophilus influenzae type b (Hib) seroprevalence in France: impact of vaccination schedules.* BMC Infect Dis, 2021. **21**(1): p. 715.
225. Ladhani, S., et al., *Haemophilus influenzae serotype B (Hib) seroprevalence in England and Wales in 2009.* Euro Surveill, 2012. **17**(46).
226. Anderson, K.S., et al., *Pre-diagnostic dynamic HPV16 IgG seropositivity and risk of oropharyngeal cancer.* Oral Oncol, 2017. **73**: p. 132-137.
227. Andersson, K., et al., *Prospective study of genital human papillomaviruses and nonmelanoma skin cancer.* Int J Cancer, 2013. **133**(8): p. 1840-5.
228. Andersson, K., et al., *Prospective study of human papillomavirus seropositivity and risk of nonmelanoma skin cancer.* Am J Epidemiol, 2012. **175**(7): p. 685-95.
229. Arnheim Dahlstrom, L., et al., *Prospective seroepidemiologic study of human papillomavirus and other risk factors in cervical cancer.* Cancer Epidemiol Biomarkers Prev, 2011. **20**(12): p. 2541-50.
230. Bjorge, T., et al., *Prospective seroepidemiological study of role of human papillomavirus in non-cervical anogenital cancers.* BMJ, 1997. **315**(7109): p. 646-9.
231. Bjorge, T., et al., *Human papillomavirus infection as a risk factor for anal and perianal skin cancer in a prospective study.* Br J Cancer, 2002. **87**(1): p. 61-4.
232. Chen, C.J., et al., *Seroprevalence of human papillomavirus types 16 and 18 in the general population in Taiwan: implication for optimal age of human papillomavirus vaccination.* J Clin Virol, 2007. **38**(2): p. 126-30.
233. Cubie, H.A., et al., *Presence of antibodies to human papillomavirus virus-like particles (VLPs) in 11-13-year-old schoolgirls.* J Med Virol, 1998. **56**(3): p. 210-6.
234. Desai, S., et al., *Prevalence of human papillomavirus antibodies in males and females in England.* Sex Transm Dis, 2011. **38**(7): p. 622-9.

235. Dillner, J., et al., *Sero-epidemiologal association between human-papillomavirus infection and risk of prostate cancer.* International Journal of Cancer, 1998. **75**(4): p. 564-567.
236. Faust, H., et al., *Cutaneous Human Papillomaviruses and Squamous Cell Carcinoma of the Skin: Nested Case-Control Study.* Cancer Epidemiol Biomarkers Prev, 2016. **25**(4): p. 721-4.
237. Gorander, S., et al., *Seroprevalences of herpes simplex virus type 2, five oncogenic human papillomaviruses, and Chlamydia trachomatis in Katowice, Poland.* Clin Vaccine Immunol, 2008. **15**(4): p. 675-80.
238. Hagensee, M.E., et al., *Seroprevalence of human papillomavirus type 16 in pregnant women.* Obstet Gynecol, 1999. **94**(5 Pt 1): p. 653-8.
239. Hamsikova, E., et al., *Cross-sectional study on the prevalence of HPV antibodies in the general population of the Czech Republic.* Sex Transm Infect, 2013. **89**(2): p. 133-7.
240. Heino, P., et al., *Association of serum immunoglobulin G antibodies against human papillomavirus type 16 capsids with anal epidermoid carcinoma.* J Natl Cancer Inst, 1995. **87**(6): p. 437-40.
241. Kann, H., et al., *Sustained Cross-reactive Antibody Responses After Human Papillomavirus Vaccinations: Up to 12 Years Follow-up in the Finnish Maternity Cohort.* J Infect Dis, 2021. **223**(11): p. 1992-2000.
242. Khoo, N.K., et al., *Comparative seroepidemiology of genital human papillomavirus infections in the general population in Singapore.* Public Health, 2017. **142**: p. 1-3.
243. Korodi, Z., et al., *Human papillomavirus 16, 18, and 33 infections and risk of prostate cancer: a Nordic nested case-control study.* Cancer Epidemiol Biomarkers Prev, 2005. **14**(12): p. 2952-5.
244. Kreimer, A.R., et al., *Timing of HPV16-E6 antibody seroconversion before OPSCC: findings from the HPVC3 consortium.* Ann Oncol, 2019. **30**(8): p. 1335-1343.
245. Laukkanen, P., et al., *Time trends in incidence and prevalence of human papillomavirus type 6, 11 and 16 infections in Finland.* J Gen Virol, 2003. **84**(Pt 8): p. 2105-2109.
246. Lehtinen, M., et al., *Seroprevalence atlas of infections with oncogenic and non-oncogenic human papillomaviruses in Finland in the 1980s and 1990s.* Int J Cancer, 2006. **119**(11): p. 2612-9.
247. Loenenbach, A., et al., *Seroprevalence of mucosal and cutaneous human papillomavirus (HPV) types among children and adolescents in the general population in Germany.* BMC Infect Dis, 2022. **22**(1): p. 44.
248. Loenenbach, A.D., et al., *Mucosal and cutaneous Human Papillomavirus seroprevalence among adults in the prevaccine era in Germany - Results from a nationwide population-based survey.* Int J Infect Dis, 2019. **83**: p. 3-11.
249. Lumme, S., et al., *Longitudinal biobanks-based study on the joint effects of infections, nutrition and hormones on risk of prostate cancer.* Acta Oncol, 2016. **55**(7): p. 839-45.
250. Luostarinen, T., et al., *No excess risk of cervical carcinoma among women seropositive for both HPV16 and HPV6/11.* International Journal of Cancer, 1999. **80**(6): p. 818-822.
251. Marais, D., R.C. Rose, and A.L. Williamson, *Age distribution of antibodies to human papillomavirus in children, women with cervical intraepithelial neoplasia and blood donors from South Africa.* J Med Virol, 1997. **51**(2): p. 126-31.
252. Marais, D.J., et al., *Seroresponses to human papillomavirus types 16, 18, 31, 33, and 45 virus-like particles in South African women with cervical cancer and cervical intraepithelial neoplasia.* J Med Virol, 2000. **60**(4): p. 403-10.
253. Mesher, D., et al., *HPV Serology Testing Confirms High HPV Immunisation Coverage in England.* PLoS One, 2016. **11**(3): p. e0150107.
254. Mesher, D., et al., *Post-vaccination HPV seroprevalence among female sexual health clinic attenders in England.* Vaccine, 2021. **39**(30): p. 4210-4218.
255. Michael, K.M., et al., *Seroprevalence of 34 human papillomavirus types in the German general population.* PLoS Pathog, 2008. **4**(6): p. e1000091.
256. Mousa, M., et al., *Prevalence of human papillomavirus in Jeddah, Saudi Arabia.* Ann Saudi Med, 2019. **39**(6): p. 403-409.

257. Newall, A.T., et al., *Population seroprevalence of human papillomavirus types 6, 11, 16, and 18 in men, women, and children in Australia.* Clin Infect Dis, 2008. **46**(11): p. 1647-55.
258. Pillsbury, A.J., et al., *Population-Level Herd Protection of Males From a Female Human Papillomavirus Vaccination Program: Evidence from Australian Serosurveillance.* Clin Infect Dis, 2017. **65**(5): p. 827-832.
259. Rahman, S., et al., *Seroprevalence of Chlamydia trachomatis, herpes simplex 2, Epstein-Barr virus, hepatitis C and associated factors among a cohort of men ages 18-70 years from three countries.* PLoS One, 2021. **16**(6): p. e0253005.
260. Scherpenisse, M., et al., *Seroprevalence of seven high-risk HPV types in The Netherlands.* Vaccine, 2012. **30**(47): p. 6686-93.
261. Scherpenisse, M., et al., *Changes in antibody seroprevalence of seven high-risk HPV types between nationwide surveillance studies from 1995-96 and 2006-07 in The Netherlands.* PLoS One, 2012. **7**(11): p. e48807.
262. Sitas, F., et al., *InterSCOPE study: Associations between esophageal squamous cell carcinoma and human papillomavirus serological markers.* J Natl Cancer Inst, 2012. **104**(2): p. 147-58.
263. Stone, K.M., et al., *Seroprevalence of human papillomavirus type 16 infection in the United States.* J Infect Dis, 2002. **186**(10): p. 1396-402.
264. Strickler, H.D., et al., *HPV 16 antibody prevalence in Jamaica and the United States reflects differences in cervical cancer rates.* Int J Cancer, 1999. **80**(3): p. 339-44.
265. Tanser, F., et al., *Human papillomavirus seropositivity and subsequent risk of HIV acquisition in rural South African women.* Sex Transm Dis, 2013. **40**(7): p. 601-6.
266. Van Doornum, G.J., et al., *Reactivity to human papillomavirus type 16 L1 virus-like particles in sera from patients with genital cancer and patients with carcinomas at five different extragenital sites.* Br J Cancer, 2003. **88**(7): p. 1095-100.
267. von Witzleben, A., et al., *Correlation of HPV16 Gene Status and Gene Expression With Antibody Seropositivity and TIL Status in OPSCC.* Front Oncol, 2020. **10**: p. 591063.
268. Wang, J.W., et al., *Seroepidemiology of Human Papillomavirus 16 (HPV16) L2 and Generation of L2-Specific Human Chimeric Monoclonal Antibodies.* Clin Vaccine Immunol, 2015. **22**(7): p. 806-16.
269. Kramer, M., et al., *Age-specific HPV seroprevalence among young females in The Netherlands.* Sexually Transmitted Infections, 2010. **86**(7): p. 494-499.
270. Achonu, C., et al., *Seroprevalence of pandemic influenza H1N1 in Ontario from January 2009-May 2010.* PLoS One, 2011. **6**(11): p. e26427.
271. Alladi, C.S.H., et al., *Hemagglutination Inhibition Antibody Response Following Influenza A(H1N1)pdm09 Virus Natural Infection: A Cross-Sectional Study from Thirthahalli, Karnataka, India.* Viral Immunol, 2019. **32**(5): p. 230-233.
272. Babu, T.M., et al., *Population Serologic Immunity to Human and Avian H2N2 Viruses in the United States and Hong Kong for Pandemic Risk Assessment.* J Infect Dis, 2018. **218**(7): p. 1054-1060.
273. Bone, A., et al., *Incidence of H1N1 2009 virus infection through the analysis of paired plasma specimens among blood donors, France.* PLoS One, 2012. **7**(3): p. e33056.
274. Boni, M.F., et al., *Population-level antibody estimates to novel influenza A/H7N9.* J Infect Dis, 2013. **208**(4): p. 554-8.
275. Booy, R., et al., *Cross-reacting antibodies against the pandemic (H1N1) 2009 influenza virus in older Australians.* Med J Aust, 2011. **194**(1): p. 19-23.
276. Chen, C.J., et al., *Seroprevalence and severity of 2009 pandemic influenza A H1N1 in Taiwan.* PLoS One, 2011. **6**(9): p. e24440.
277. Cheung, J.T.L., et al., *Determining Existing Human Population Immunity as Part of Assessing Influenza Pandemic Risk.* Emerg Infect Dis, 2022. **28**(5): p. 977-985.
278. Chi, C.Y., et al., *Preexisting antibody response against 2009 pandemic influenza H1N1 viruses in the Taiwanese population.* Clin Vaccine Immunol, 2010. **17**(12): p. 1958-62.

279. Cox, C.M., et al., *Prevalence of 2009 pandemic influenza A (H1N1) virus antibodies, Tampa Bay Florida--November-December, 2009.* PLoS One, 2011. **6**(12): p. e29301.
280. Desheva, Y.A., et al., *Serum strain-specific or cross-reactive neuraminidase inhibiting antibodies against pandemic capital A, Cyrillic/California/07/2009(H1N1) influenza in healthy volunteers.* BMC Res Notes, 2015. **8**(1): p. 136.
281. Dowse, G.K., et al., *Incidence of pandemic (H1N1) 2009 influenza infection in children and pregnant women during the 2009 influenza season in Western Australia - a seroprevalence study.* Med J Aust, 2011. **194**(2): p. 68-72.
282. Dudareva, S., et al., *Prevalence of antibodies to 2009 pandemic influenza A (H1N1) virus in German adult population in pre- and post-pandemic period.* PLoS One, 2011. **6**(6): p. e21340.
283. El Rhaffouli, H., et al., *[Seroprevalence of pandemic influenza A (H1N1)pdm09 in two regions in Morocco following the 2010-2011 influenza season].* Pathol Biol (Paris), 2013. **61**(2): p. 83-6.
284. Guiomar, R., et al., *Cross-protection to new drifted influenza A(H3) viruses and prevalence of protective antibodies to seasonal influenza, during 2014 in Portugal.* Vaccine, 2017. **35**(16): p. 2092-2099.
285. Hackenberg, A., et al., *Antibody prevalence to the 2009 pandemic influenza A (H1N1) virus in Germany: geographically variable immunity in winter 2010/2011.* Med Microbiol Immunol, 2013. **202**(1): p. 87-94.
286. Hancock, K., et al., *Cross-reactive antibody responses to the 2009 pandemic H1N1 influenza virus.* N Engl J Med, 2009. **361**(20): p. 1945-52.
287. Hardelid, P., et al., *Assessment of baseline age-specific antibody prevalence and incidence of infection to novel influenza A/H1N1 2009.* Health Technol Assess, 2010. **14**(55): p. 115-92.
288. Hoschler, K., et al., *Seroprevalence of influenza A(H1N1)pdm09 virus antibody, England, 2010 and 2011.* Emerg Infect Dis, 2012. **18**(11): p. 1894-7.
289. Huo, X., et al., *Seroprevalence of pandemic (H1N1) 2009 in pregnant women in China: an observational study.* PLoS One, 2011. **6**(3): p. e17995.
290. Khuntirat, B., et al., *Absence of neutralizing antibodies against influenza A/H5N1 virus among children in Kamphaeng Phet, Thailand.* J Clin Virol, 2015. **69**: p. 78-80.
291. Kok, J., et al., *Pandemic (H1N1) 2009 influenza virus seroconversion rates in HIV-infected individuals.* J Acquir Immune Defic Syndr, 2011. **56**(2): p. 91-4.
292. Lerdsamran, H., et al., *Serological response to the 2009 pandemic influenza A (H1N1) virus for disease diagnosis and estimating the infection rate in Thai population.* PLoS One, 2011. **6**(1): p. e16164.
293. Lin, Y.P., et al., *Population seroprevalence of antibody to influenza A(H7N9) virus, Guangzhou, China.* BMC Infect Dis, 2016. **16**(1): p. 632.
294. Liu, X., et al., *Pre-existing immunity with high neutralizing activity to 2009 pandemic H1N1 influenza virus in Shanghai population.* PLoS One, 2013. **8**(3): p. e58810.
295. Lorbach, J.N., et al., *Gaps in Serologic Immunity against Contemporary Swine-Origin Influenza A Viruses among Healthy Individuals in the United States.* Viruses, 2021. **13**(1): p. 127-127.
296. Mahmud, S.M., et al., *Estimated cumulative incidence of pandemic (H1N1) influenza among pregnant women during the first wave of the 2009 pandemic.* CMAJ, 2010. **182**(14): p. 1522-4.
297. Mandelboim, M., et al., *Significant cross reactive antibodies to influenza virus in adults and children during a period of marked antigenic drift.* BMC Infect Dis, 2014. **14**(1): p. 346.
298. McVernon, J., et al., *Seroprevalence of antibody to influenza A(H1N1)pdm09 attributed to vaccination or infection, before and after the second (2010) pandemic wave in Australia.* Influenza Other Respir Viruses, 2014. **8**(2): p. 194-200.
299. McVernon, J., et al., *Seroprevalence of 2009 pandemic influenza A(H1N1) virus in Australian blood donors, October - December 2009.* Euro Surveill, 2010. **15**(40).
300. Mesman, A.W., et al., *Influenza virus A(H1N1)2009 antibody-dependent cellular cytotoxicity in young children prior to the H1N1 pandemic.* J Gen Virol, 2016. **97**(9): p. 2157-2165.

301. Miller, E., et al., *Incidence of 2009 pandemic influenza A H1N1 infection in England: a cross-sectional serological study.* Lancet, 2010. **375**(9720): p. 1100-8.
302. Nhat, N.T.D., et al., *Structure of general-population antibody titer distributions to influenza A virus.* Sci Rep, 2017. **7**(1): p. 6060.
303. Pichyangkul, S., et al., *Pre-existing cross-reactive antibodies to avian influenza H5N1 and 2009 pandemic H1N1 in US military personnel.* Am J Trop Med Hyg, 2014. **90**(1): p. 149-52.
304. Radin, J.M., et al., *Seroprotective antibodies to 2011 variant influenza A(H3N2v) and seasonal influenza A(H3N2) among three age groups of US Department of Defense service members.* PLoS One, 2015. **10**(3): p. e0121037.
305. Rizzo, C., et al., *Cross-reactive antibody responses to the 2009 A/H1N1v influenza virus in the Italian population in the pre-pandemic period.* Vaccine, 2010. **28**(20): p. 3558-62.
306. Sam, I.C., et al., *Seroprevalence of seasonal and pandemic influenza a in Kuala Lumpur, Malaysia in 2008-2010.* J Med Virol, 2013. **85**(8): p. 1420-5.
307. Sanyal, M., et al., *Prevalence of antibodies against seasonal influenza A and B viruses among older adults in rural Thailand: A cross-sectional study.* Plos One, 2021. **16**(8): p. e0256475-e0256475.
308. Sauerbrei, A., et al., *Prevalence of antibodies against influenza A and B viruses in children in Germany, 2008 to 2010.* Euro Surveill, 2014. **19**(5).
309. Sauerbrei, A., et al., *Seroprevalence of influenza A and B in German infants and adolescents.* Med Microbiol Immunol, 2009. **198**(2): p. 93-101.
310. Skowronski, D.M., et al., *Prevalence of seroprotection against the pandemic (H1N1) virus after the 2009 pandemic.* CMAJ, 2010. **182**(17): p. 1851-6.
311. Skowronski, D.M., et al., *Immuno-epidemiologic correlates of pandemic H1N1 surveillance observations: higher antibody and lower cell-mediated immune responses with advanced age.* J Infect Dis, 2011. **203**(2): p. 158-67.
312. Skowronski, D.M., et al., *Cross-reactive and vaccine-induced antibody to an emerging swine-origin variant of influenza A virus subtype H3N2 (H3N2v).* J Infect Dis, 2012. **206**(12): p. 1852-61.
313. Tandale, B.V., et al., *Seroepidemiology of pandemic influenza A (H1N1) 2009 virus infections in Pune, India.* BMC Infect Dis, 2010. **10**(1): p. 255.
314. Trombetta, C.M., et al., *Influenza D Virus: Serological Evidence in the Italian Population from 2005 to 2017.* Viruses, 2019. **12**(1): p. 30-30.
315. Trombetta, C.M., et al., *Detection of antibodies against influenza D virus in swine veterinarians in Italy in 2004.* J Med Virol, 2022. **94**(6): p. 2855-2859.
316. Veguilla, V., et al., *A Large Proportion of the Mexican Population Remained Susceptible to A(H1N1)pdm09 Infection One Year after the Emergence of 2009 Influenza Pandemic.* PLoS One, 2016. **11**(3): p. e0150428.
317. Vinh, D.N., et al., *Age-seroprevalence curves for the multi-strain structure of influenza A virus.* Nat Commun, 2021. **12**(1): p. 6680.
318. Wang, W., et al., *Serologic assay for avian-origin influenza A (H7N9) virus in adults of Shanghai, Guangzhou and Yunnan, China.* J Clin Virol, 2014. **60**(3): p. 305-8.
319. Wu, J.T., et al., *Estimating infection attack rates and severity in real time during an influenza pandemic: analysis of serial cross-sectional serologic surveillance data.* PLoS Med, 2011. **8**(10): p. e1001103.
320. Zhang, R., et al., *Determination of serum neutralization antibodies against seasonal influenza A strain H3N2 and the emerging strains 2009 H1N1 and avian H5N1.* Scand J Infect Dis, 2011. **43**(3): p. 216-20.
321. Zhu, H., et al., *Low population serum microneutralization antibody titer against the predominating influenza A(H3N2) N121K virus during the severe influenza summer peak of Hong Kong in 2017.* Emerg Microbes Infect, 2018. **7**(1): p. 23.

322. Zimmer, S.M., et al., *Seroprevalence following the second wave of Pandemic 2009 H1N1 influenza in Pittsburgh, PA, USA.* PLoS One, 2010. **5**(7): p. e11601.
323. Hsu, L.C., et al., *The incidence of Japanese encephalitis in Taiwan--a population-based study.* PLoS Negl Trop Dis, 2014. **8**(7): p. e3030.
324. Krow-Lucal, E.R., et al., *Immune response at 12-23 months following a single dose of Vero cell culture-derived Japanese encephalitis (JE) vaccine in adults previously vaccinated with mouse brain-derived JE vaccine.* Vaccine, 2020. **38**(44): p. 6899-6903.
325. Andrews, N., et al., *Towards elimination: measles susceptibility in Australia and 17 European countries.* Bull World Health Organ, 2008. **86**(3): p. 197-204.
326. Ang, L.W., et al., *Prevalence of measles antibodies among migrant workers in Singapore: a serological study to identify susceptible population subgroups.* BMC Infect Dis, 2022. **22**(1): p. 88.
327. Ang, L.W., et al., *Prevalence of antibodies against measles, mumps and rubella in the childhood population in Singapore, 2008-2010.* Epidemiol Infect, 2013. **141**(8): p. 1721-30.
328. Bassal, R., et al., *Seropositivity of measles antibodies in the Israeli population prior to the nationwide 2018 - 2019 outbreak.* Hum Vaccin Immunother, 2021. **17**(5): p. 1353-1357.
329. Berglov, A., et al., *Prevalence of herpes -, measles morbillivirus-, parvovirus B19 - and rubella viruses immunoglobulin G among women with chronic hepatitis B of reproductive age in Denmark: A cross-sectional study.* Int J Infect Dis, 2020. **101**: p. 269-275.
330. Bolotin, S., et al., *Assessment of population immunity to measles in Ontario, Canada: a Canadian Immunization Research Network (CIRN) study.* Hum Vaccin Immunother, 2019. **15**(12): p. 2856-2864.
331. Borocz, K., et al., *Application of a fast and cost-effective 'three-in-one' MMR ELISA as a tool for surveying anti-MMR humoral immunity: the Hungarian experience.* Epidemiol Infect, 2020. **148**: p. e17.
332. Carcelen, A.C., et al., *Leveraging a national biorepository in Zambia to assess measles and rubella immunity gaps across age and space.* Sci Rep, 2022. **12**(1): p. 10217.
333. Castro-Silva, R., et al., *Serological surveillance of measles in blood donors in Rio de Janeiro, Brazil.* Rev Panam Salud Publica, 2003. **14**(5): p. 334-40.
334. Chen, R.T., et al., *Measles antibody: reevaluation of protective titers.* J Infect Dis, 1990. **162**(5): p. 1036-42.
335. Choisy, M., et al., *Sero-Prevalence Surveillance to Predict Vaccine-Preventable Disease Outbreaks; A Lesson from the 2014 Measles Epidemic in Northern Vietnam.* Open Forum Infect Dis, 2019. **6**(3): p. ofz030.
336. Ehresmann, K.R., et al., *An outbreak of measles among unvaccinated young adults and measles seroprevalence study: implications for measles outbreak control in adult populations.* J Infect Dis, 2004. **189 Suppl 1**(Supplement_1): p. S104-7.
337. Ferson, M.J., P.W. Robertson, and J.W. Donovan, *Measles immunity and immunization status in under-5-year-old children in New South Wales: a population-based study.* J Paediatr Child Health, 1998. **34**(4): p. 339-41.
338. Gidding, H.F., et al., *Declining measles antibodies in the era of elimination: Australia's experience.* Vaccine, 2018. **36**(4): p. 507-513.
339. Hagstam, P., B. Bottiger, and N. Winqvist, *Measles and rubella seroimmunity in newly arrived adult immigrants in Sweden.* Infect Dis (Lond), 2019. **51**(2): p. 122-130.
340. Janaszek, W., W. Gut, and N.J. Gay, *The epidemiology of measles in Poland: prevalence of measles virus antibodies in the population.* Epidemiol Infect, 2000. **125**(2): p. 385-92.
341. Kelly, H.A., et al., *Measles immunity among young adults in Victoria.* Communicable diseases intelligence quarterly report, 2001. **25**(3): p. 129-32.
342. Kennedy, C.M., B.A. Burns, and K.A. Ault, *Does rubella immunity predict measles immunity? A serosurvey of pregnant women.* Infect Dis Obstet Gynecol, 2006. **2006**: p. 13890.

343. Khetsuriani, N., et al., *Measles and rubella seroprevalence among adults in Georgia in 2015: helping guide the elimination efforts.* Epidemiol Infect, 2019. **147**: p. e319.
344. Kim, E.S., et al., *Seroprevalence of measles among children affected by national measles elimination program in Korea, 2010.* Vaccine, 2012. **30**(23): p. 3355-9.
345. Ma, C., et al., *Measles vaccine coverage estimates in an outbreak three years after the nation-wide campaign in China: implications for measles elimination, 2013.* BMC Infect Dis, 2015. **15**(1): p. 23.
346. Makowka, A., et al., *Seroprevalence of measles-specific antibodies in the group predominantly affected by measles in 2006-2009 in Poland.* Przegl Epidemiol, 2014. **68**(3): p. 411-6, 521-5.
347. Marquis, S.R., et al., *Seroprevalence of Measles and Mumps Antibodies Among Individuals With Cancer.* JAMA Netw Open, 2021. **4**(7): p. e2118508.
348. Mburu, C.N., et al., *The importance of supplementary immunisation activities to prevent measles outbreaks during the COVID-19 pandemic in Kenya.* BMC Med, 2021. **19**(1): p. 35.
349. Osman, S., et al., *Population immunity to measles in Canada using Canadian Health Measures survey data - A Canadian Immunization Research Network (CIRN) study.* Vaccine, 2022. **40**(23): p. 3228-3235.
350. Persson Berg, L., et al., *Serum IgG levels to Epstein-Barr and measles viruses in patients with multiple sclerosis during natalizumab and interferon beta treatment.* BMJ Neurol Open, 2022. **4**(2): p. e000271.
351. Ristic, M., et al., *Sero-epidemiological study in prediction of the risk groups for measles outbreaks in Vojvodina, Serbia.* PLoS One, 2019. **14**(5): p. e0216219.
352. Rosario-Rosario, G., et al., *Using Locally Derived Seroprevalence Data on Measles, Mumps, Rubella, and Varicella by Birth Cohort to Determine Risks for Vaccine-Preventable Diseases During International Travel.* J Travel Med, 2015. **22**(6): p. 396-402.
353. Seruyange, E., et al., *Measles seroprevalence, outbreaks, and vaccine coverage in Rwanda.* Infect Dis (Lond), 2016. **48**(11-12): p. 800-7.
354. Tang, L., et al., *Measles epidemics and seroepidemiology of population in Wujin, Changzhou city, Jiangsu province, China 2015.* Vaccine, 2017. **35**(22): p. 2925-2929.
355. Tharmaphornpilas, P., et al., *Seroprevalence of antibodies to measles, mumps, and rubella among Thai population: evaluation of measles/MMR immunization programme.* J Health Popul Nutr, 2009. **27**(1): p. 80-6.
356. Wanlapakorn, N., et al., *Antibodies against measles and rubella virus among different age groups in Thailand: A population-based serological survey.* PLoS One, 2019. **14**(11): p. e0225606.
357. Zabeida, A., et al., *Reevaluating immunization delays after red blood cell transfusion.* Transfusion, 2019. **59**(9): p. 2806-2811.
358. Zhou, R., et al., *Measles immunity in the China-Myanmar border region, Lincang city, Yunnan province, 2017.* Hum Vaccin Immunother, 2020. **16**(4): p. 881-885.
359. Eriksen, J., et al., *Seroepidemiology of mumps in Europe (1996-2008): why do outbreaks occur in highly vaccinated populations?* Epidemiol Infect, 2013. **141**(3): p. 651-66.
360. Huerta, M., et al., *Declining population immunity to mumps among Israeli military recruits.* Vaccine, 2006. **24**(37-39): p. 6300-3.
361. Levine, H., et al., *Sub-optimal prevalence of mumps antibodies in a population based study of young adults in Israel after 20 years of two dose universal vaccination policy.* Vaccine, 2011. **29**(15): p. 2785-90.
362. Muhsen, K., et al., *Prevalence of mumps antibodies in the Israeli population in relation to mumps vaccination policy and incidence of disease.* Epidemiol Infect, 2008. **136**(5): p. 688-93.
363. Muhsen, K., et al., *Sero-prevalence of mumps antibodies in subpopulations subsequently affected by a large scale mumps epidemic in Israel.* Vaccine, 2011. **29**(22): p. 3878-82.
364. Okabayashi, H., et al., *Seroprevalence of mumps before the introduction of mumps-containing vaccine in Lao PDR: results from a nationwide cross-sectional population-based survey.* BMC Res Notes, 2019. **12**(1): p. 155.

365. Pang, H., et al., *Seroprevalence and Determinants Associated with Mumps Antibodies after 20 Years of MMR Vaccination in Urban Area of Shanghai, China.* Int J Environ Res Public Health, 2018. **15**(10): p. 2089-2089.
366. Patel, C., et al., *Australian mumps serosurvey 2012-2013: any cause for concern?* Commun Dis Intell (2018), 2020. **44**.
367. Backhouse, J.L., et al., *Population-based seroprevalence of Neisseria meningitidis serogroup C capsular antibody before the introduction of conjugate vaccine, in Australia.* Vaccine, 2007. **25**(7): p. 1310-5.
368. de Voer, R.M., et al., *Immunity against Neisseria meningitidis serogroup C in the Dutch population before and after introduction of the meningococcal c conjugate vaccine.* PLoS One, 2010. **5**(8): p. e12144.
369. Brooks, J.I., et al., *Low levels of detectable pertussis antibody among a large cohort of pregnant women in Canada.* Vaccine, 2018. **36**(41): p. 6138-6143.
370. Cagney, M., et al., *The seroepidemiology of pertussis in Australia during an epidemic period.* Epidemiol Infect, 2006. **134**(6): p. 1208-16.
371. Campbell, P., et al., *Increased population prevalence of low pertussis toxin antibody levels in young children preceding a record pertussis epidemic in Australia.* PLoS One, 2012. **7**(4): p. e35874.
372. Fallo, A., et al., *Pertussis seroprevalence in adults, post-partum women and umbilical cord blood.* Arch Argent Pediatr, 2014. **112**(4): p. 315-22.
373. Farahat, F., et al., *Cross-sectional seroprevalence study of antibody to Bordetella pertussis toxin in western Saudi Arabia: is there a need for a vaccine booster dose for adolescents and young adults?* BMJ Open, 2021. **11**(4).
374. Huygen, K., et al., *Bordetella pertussis seroprevalence in Belgian adults aged 20-39 years, 2012.* Epidemiol Infect, 2014. **142**(4): p. 724-8.
375. Jogi, P., et al., *Seroprevalence of IgG antibodies to pertussis toxin in children and adolescents in Estonia.* Vaccine, 2014. **32**(41): p. 5311-5.
376. Jogi, P., et al., *Estimated and reported incidence of pertussis in Estonian adults: A seroepidemiological study.* Vaccine, 2015. **33**(38): p. 4756-61.
377. Kennerknecht, N., et al., *Pertussis surveillance by small serosurveys of blood donors.* Epidemiol Infect, 2018. **146**(14): p. 1807-1810.
378. Kleine, D., et al., *Pertussis in Lao PDR: Seroprevalence and disease.* Int J Infect Dis, 2020. **95**: p. 282-287.
379. Lee, S.Y., et al., *Pertussis seroprevalence in korean adolescents and adults using anti-pertussis toxin immunoglobulin G.* J Korean Med Sci, 2014. **29**(5): p. 652-6.
380. Quinn, H.E., et al., *The utility of seroepidemiology for tracking trends in pertussis infection.* Epidemiol Infect, 2010. **138**(3): p. 426-33.
381. Raeven, R.H.M., et al., *Antibody Specificity Following a Recent Bordetella pertussis Infection in Adolescence Is Correlated With the Pertussis Vaccine Received in Childhood.* Front Immunol, 2019. **10**(JUN): p. 1364.
382. Rendi-Wagner, P., et al., *The seroepidemiology of Bordetella pertussis in Israel--Estimate of incidence of infection.* Vaccine, 2010. **28**(19): p. 3285-90.
383. Wehlin, L., et al., *Pertussis seroprevalence among adults of reproductive age (20-39 years) in fourteen European countries.* APMIS, 2021. **129**(9): p. 556-565.
384. Wright, S.W., M.D. Decker, and K.M. Edwards, *Incidence of pertussis infection in healthcare workers.* Infect Control Hosp Epidemiol, 1999. **20**(2): p. 120-3.
385. Diedrich, S., H. Claus, and E. Schreier, *Immunity status against poliomyelitis in Germany: determination of cut-off values in International Units.* BMC Infect Dis, 2002. **2**(1): p. 2.
386. Giammanco, G.M., et al., *Is Italian population protected from Poliovirus? Results of a seroprevalence survey in Florence, Italy.* Hum Vaccin Immunother, 2018. **14**(9): p. 2248-2253.

387. Hendry, A.J., et al., *Lower immunity to poliomyelitis viruses in Australian young adults not eligible for inactivated polio vaccine.* Vaccine, 2020. **38**(11): p. 2572-2577.
388. Kulshammer, M., et al., *Poor immunity status against poliomyelitis in medical students: a semi-anonymous study.* Med Microbiol Immunol, 2013. **202**(1): p. 63-5.
389. Pauly, M., et al., *Immunity levels to poliovirus in Lao children and adults before the vaccine-derived poliovirus outbreak: A retrospective study.* PLoS One, 2018. **13**(5): p. e0197370.
390. Schoub, B.D., et al., *A population-based seroprevalence study in South Africa as a tool in the polio eradication initiative.* Am J Trop Med Hyg, 1998. **58**(5): p. 650-4.
391. Tafuri, S., et al., *Serological survey on immunity status against polioviruses in children and adolescents living in a border region, Apulia (Southern Italy).* BMC Infect Dis, 2008. **8**(1): p. 150.
392. Hull, J.J., et al., *Rotavirus antigen, cytokine, and neutralising antibody profiles in sera of children with and without HIV infection in Blantyre, Malawi.* Malawi Med J, 2017. **29**(1): p. 24-28.
393. Angsuwatcharakon, P., et al., *High seroprevalence of rubella in Thai children with a 2-dose MMR national immunization policy.* Vaccine, 2021. **39**(42): p. 6206-6209.
394. Caidi, H., et al., *Rubella seroprevalence among women aged 15-39 years in Morocco.* Eastern Mediterranean Health Journal, 2009. **15**(3): p. 526-531.
395. Cohen, D., et al., *Use of rubella seroepidemiological data for assessment of previous vaccination policy and for decision making in response to epidemics in Israel.* Vaccine, 2006. **24**(27-28): p. 5604-8.
396. Crooke, S.N., et al., *Seroprevalence and durability of rubella virus antibodies in a highly immunized population.* Vaccine, 2019. **37**(29): p. 3876-3882.
397. Edirisuriya, C., et al., *Australian rubella serosurvey 2012-2013: On track for elimination?* Vaccine, 2018. **36**(20): p. 2794-2798.
398. Gilbert, N.L., et al., *Seroprevalence of rubella antibodies and determinants of susceptibility to rubella in a cohort of pregnant women in Canada, 2008-2011.* Vaccine, 2017. **35**(23): p. 3050-3055.
399. Huerta, M., et al., *Declining seroprevalence of rubella antibodies among young Israeli adults: a 12-year comparison.* Prev Med, 2004. **39**(6): p. 1223-6.
400. Jonas, A., et al., *Rubella immunity among pregnant women aged 15-44 years, Namibia, 2010.* Int J Infect Dis, 2016. **49**: p. 196-201.
401. Kelly, H., et al., *Interruption of rubella virus transmission in Australia may require vaccination of adult males: evidence from a Victorian sero-survey.* Commun Dis Intell Q Rep, 2004. **28**(1): p. 69-73.
402. Kusumoto, K., et al., *Rubella outbreak on Tokunoshima Island in 2004: a population-based study of pregnant women.* J Obstet Gynaecol Res, 2010. **36**(5): p. 938-43.
403. Motaze, N.V., et al., *Rubella seroprevalence using residual samples from the South African measles surveillance program: a cross-sectional analytic study.* Hum Vaccin Immunother, 2020. **16**(11): p. 2656-2662.
404. Nardone, A., et al., *Comparison of rubella seroepidemiology in 17 countries: progress towards international disease control targets.* Bull World Health Organ, 2008. **86**(2): p. 118-25.
405. Patic, A., et al., *Seroepidemiological study of rubella in Vojvodina, Serbia: 24 years after the introduction of the MMR vaccine in the national immunization programme.* PLoS One, 2020. **15**(1): p. e0227413.
406. Pebody, R.G., et al., *The seroepidemiology of rubella in western Europe.* Epidemiol Infect, 2000. **125**(2): p. 347-57.
407. Rota, M.C., et al., *Rubella seroprofile of the Italian population: an 8-year comparison.* Epidemiol Infect, 2007. **135**(4): p. 555-62.
408. Schoub, B.D., et al., *Rubella in South Africa: An impending greek tragedy?* S. Afr. Med. J., 2009. **99**(7): p. 515-519.

409. Su, Q., et al., *Assessing the burden of congenital rubella syndrome in China and evaluating mitigation strategies: a metapopulation modelling study.* Lancet Infect Dis, 2021. **21**(7): p. 1004-1013.
410. Adetifa, I.M.O., et al., *Temporal trends of SARS-CoV-2 seroprevalence during the first wave of the COVID-19 epidemic in Kenya.* Nat Commun, 2021. **12**(1): p. 3966.
411. Alandijany, T.A., et al., *Lack of Antibodies to SARS-CoV-2 among Blood Donors during COVID-19 Lockdown: A Study from Saudi Arabia.* Healthcare (Basel), 2021. **9**(1).
412. Alenazi, M.W., et al., *Seroprevalence of COVID-19 in Riyadh city during the early increase of COVID-19 infections in Saudi Arabia, June 2020.* Saudi J Biol Sci, 2022. **29**(6): p. 103282.
413. Alharbi, N.K., et al., *Nationwide Seroprevalence of SARS-CoV-2 in Saudi Arabia.* J Infect Public Health, 2021. **14**(7): p. 832-838.
414. Alzabeedi, K.H., et al., *High Seroprevalence of SARS-CoV-2 IgG and RNA among Asymptomatic Blood Donors in Makkah Region, Saudi Arabia.* Vaccines (Basel), 2022. **10**(8).
415. Amirthalingam, G., et al., *Seroprevalence of SARS-CoV-2 among Blood Donors and Changes after Introduction of Public Health and Social Measures, London, UK.* Emerg Infect Dis, 2021. **27**(7): p. 1795-1801.
416. Amorim Filho, L., et al., *Seroprevalence of anti-SARS-CoV-2 among blood donors in Rio de Janeiro, Brazil.* Rev Saude Publica, 2020. **54**: p. 69.
417. Anderson, E.M., et al., *Seasonal human coronavirus antibodies are boosted upon SARS-CoV-2 infection but not associated with protection.* Cell, 2021. **184**(7): p. 1858-1864 e10.
418. Arabkhazaeli, A., et al., *Positive anti-SARS-CoV-2 rapid serological test results among asymptomatic blood donors.* Transfus Clin Biol, 2022. **29**(1): p. 24-30.
419. Assefa, N., et al., *Seroprevalence of anti-SARS-CoV-2 antibodies in women attending antenatal care in eastern Ethiopia: a facility-based surveillance.* BMJ Open, 2021. **11**(11): p. e055834.
420. Bajema, K.L., et al., *Comparison of Estimated Severe Acute Respiratory Syndrome Coronavirus 2 Seroprevalence Through Commercial Laboratory Residual Sera Testing and a Community Survey.* Clin Infect Dis, 2021. **73**(9): p. e3120-e3123.
421. Bajema, K.L., et al., *Estimated SARS-CoV-2 Seroprevalence in the US as of September 2020.* JAMA Intern Med, 2021. **181**(4): p. 450-460.
422. Bakkour, S., et al., *Minipool testing for SARS-CoV-2 RNA in United States blood donors.* Transfusion, 2021. **61**(8): p. 2384-2391.
423. Basavaraju, S.V., et al., *Serologic Testing of US Blood Donations to Identify Severe Acute Respiratory Syndrome Coronavirus 2 (SARS-CoV-2)-Reactive Antibodies: December 2019-January 2020.* Clin Infect Dis, 2021. **72**(12): p. e1004-e1009.
424. Bingham, J., et al., *Estimates of prevalence of anti-SARS-CoV-2 antibodies among blood donors in South Africa in March 2022.* Res Sq, 2022: p. rs.3.rs–1687679-rs.3.rs–1687679.
425. Boehme, K.W., et al., *Pediatric SARS-CoV-2 Seroprevalence in Arkansas Over the First Year of the COVID-19 Pandemic.* J Pediatric Infect Dis Soc, 2022. **11**(6): p. 248-256.
426. Bogogiannidou, Z., et al., *Repeated Leftover Serosurvey of SARS-CoV-2 IgG Antibodies in Greece, May to August 2020.* Vaccines (Basel), 2021. **9**(5): p. 504-504.
427. Bogogiannidou, Z., et al., *Repeated leftover serosurvey of SARS-CoV-2 IgG antibodies, Greece, March and April 2020.* Euro Surveill, 2020. **25**(31).
428. Bolotin, S., et al., *Assessment of population infection with SARS-CoV-2 in Ontario, Canada, March to June 2020.* Euro Surveill, 2021. **26**(50).
429. Bolotin, S., et al., *SARS-CoV-2 Seroprevalence Survey Estimates Are Affected by Anti-Nucleocapsid Antibody Decline.* J Infect Dis, 2021. **223**(8): p. 1334-1338.
430. Borrega, R., et al., *Cross-Reactive Antibodies to SARS-CoV-2 and MERS-CoV in Pre-COVID-19 Blood Samples from Sierra Leoneans.* Viruses, 2021. **13**(11).
431. Busch, M.P., et al., *Population-Weighted Seroprevalence From Severe Acute Respiratory Syndrome Coronavirus 2 (SARS-CoV-2) Infection, Vaccination, and Hybrid Immunity Among US*

Blood Donations From January to December 2021. Clin Infect Dis, 2022. **75**(Suppl 2): p. S254-S263.
432. Buss, L., et al., *Predicting SARS-CoV-2 Variant Spread in a Completely Seropositive Population Using Semi-Quantitative Antibody Measurements in Blood Donors.* Vaccines (Basel), 2022. **10**(9).
433. Buss, L.F., et al., *Three-quarters attack rate of SARS-CoV-2 in the Brazilian Amazon during a largely unmitigated epidemic.* Science, 2021. **371**(6526): p. 288-292.
434. Butler, D., et al., *Confirmed circulation of SARS-CoV-2 in Irish blood donors prior to first national notification of infection.* J Clin Virol, 2022. **146**: p. 105045.
435. Capai, L., et al., *Seroprevalence of SARS-CoV-2 IgG Antibodies in Corsica (France), April and June 2020.* J Clin Med, 2020. **9**(11): p. 3569-3569.
436. Capai, L., et al., *Impact of the Second Epidemic Wave of SARS-CoV-2: Increased Exposure of Young People.* Front Public Health, 2021. **9**: p. 715192.
437. Carlton, L.H., et al., *Charting elimination in the pandemic: a SARS-CoV-2 serosurvey of blood donors in New Zealand.* Epidemiol Infect, 2021. **149**: p. e173.
438. Cassaniti, I., et al., *Seroprevalence of SARS-CoV-2 in blood donors from the Lodi Red Zone and adjacent Lodi metropolitan and suburban area.* Clin Microbiol Infect, 2021. **27**(6): p. 914 e1-914 e4.
439. Castro Dopico, X., et al., *Seropositivity in blood donors and pregnant women during the first year of SARS-CoV-2 transmission in Stockholm, Sweden.* J Intern Med, 2021. **290**(3): p. 666-676.
440. Chanchlani, N., et al., *Adalimumab and Infliximab Impair SARS-CoV-2 Antibody Responses: Results from a Therapeutic Drug Monitoring Study in 11 422 Biologic-Treated Patients.* J Crohns Colitis, 2022. **16**(3): p. 389-397.
441. Chang, L., et al., *The prevalence of antibodies to SARS-CoV-2 among blood donors in China.* Nat Commun, 2021. **12**(1): p. 1383.
442. Charlton, C.L., et al., *Pre-Vaccine Positivity of SARS-CoV-2 Antibodies in Alberta, Canada during the First Two Waves of the COVID-19 Pandemic.* Microbiol Spectr, 2021. **9**(1): p. e0029121.
443. Chau, N.V.V., et al., *Absence of SARS-CoV-2 antibodies in pre-pandemic plasma from children and adults in Vietnam.* Int J Infect Dis, 2021. **111**((Chau N.V.V.; Man D.N.H.; Ty D.T.B.) Hospital for Tropical Diseases, Ho Chi Minh City, Viet Nam): p. 127-129.
444. Chaves, D.G., et al., *Pro-inflammatory immune profile mediated by TNF and IFN-gamma and regulated by IL-10 is associated to IgG anti-SARS-CoV-2 in asymptomatic blood donors.* Cytokine, 2022. **154**((Chaves D.G., daniel.chaves@hemominas.mg.gov.br; da Silva Malta M.C.F.; Martins M.L.) Fundação Hemominas, Minas Gerais, Belo Horizonte, Brazil): p. 155874.
445. Chaves, D.G., et al., *SARS-CoV-2 IgG Seroprevalence among Blood Donors as a Monitor of the COVID-19 Epidemic, Brazil.* Emerg Infect Dis, 2022. **28**(4): p. 734-742.
446. Clapham, H.E., et al., *Contrasting SARS-CoV-2 epidemics in Singapore: cohort studies in migrant workers and the general population.* Int J Infect Dis, 2022. **115**: p. 72-78.
447. Couture, A., et al., *Severe Acute Respiratory Syndrome Coronavirus 2 Seroprevalence and Reported Coronavirus Disease 2019 Cases in US Children, August 2020-May 2021.* Open Forum Infect Dis, 2022. **9**(3): p. ofac044.
448. Coyle, P.V., et al., *SARS-CoV-2 seroprevalence in the urban population of Qatar: An analysis of antibody testing on a sample of 112,941 individuals.* iScience, 2021. **24**(6): p. 102646.
449. Damjanovic, A., et al., *Utility of Newborn Dried Blood Spots to Ascertain Seroprevalence of SARS-CoV-2 Antibodies Among Individuals Giving Birth in New York State, November 2019 to November 2021.* JAMA Netw Open, 2022. **5**(8): p. e2227995.
450. Davis, G., et al., *Seroprevalence of SARS-CoV-2 infection in Cincinnati Ohio USA from August to December 2020.* PLoS One, 2021. **16**(7): p. e0254667.
451. Delaugerre, C., et al., *Severe Acute Respiratory Syndrome Coronavirus 2 Seroprevalence Among HIV-Negative Participants Using Tenofovir/Emtricitabine-Based Preexposure Prophylaxis in*

2020: A Substudy of the French National Agency for Research on AIDS and Viral Hepatitis PREVENIR and Inserm SAPRIS-Sero Cohorts. Open Forum Infect Dis, 2022. **9**(7): p. ofac188.
452. Dethioux, L., et al., *SARS-CoV-2 seroprevalence in children and their family members, July-October 2020, Brussels.* Eur J Pediatr, 2022. **181**(3): p. 1009-1016.
453. Di Stefano, M., et al., *Low Prevalence of Antibodies to SARS-CoV-2 and Undetectable Viral Load in Seropositive Blood Donors from South-Eastern Italy.* Acta Haematol, 2021. **144**(5): p. 580-584.
454. Dickson, E., et al., *Enhanced surveillance of COVID-19 in Scotland: population-based seroprevalence surveillance for SARS-CoV-2 during the first wave of the epidemic.* Public Health, 2021. **190**: p. 132-134.
455. Dietrich, M.L., et al., *SARS-CoV-2 seroprevalence rates of children seeking medical care in Louisiana during the state stay at home order.* J Clin Virol Plus, 2021. **1**(4): p. 100047.
456. Dingens, A.S., et al., *Serological identification of SARS-CoV-2 infections among children visiting a hospital during the initial Seattle outbreak.* Nat Commun, 2020. **11**(1): p. 4378.
457. Dodd, R.Y., et al., *Characteristics of US Blood Donors Testing Reactive for Antibodies to SARS-CoV-2 Prior to the Availability of Authorized Vaccines.* Transfus Med Rev, 2021. **35**(3): p. 1-7.
458. Drews, S.J., et al., *Resistance of SARS-CoV-2 beta and gamma variants to plasma collected from Canadian blood donors during the spring of 2020.* Transfusion, 2022. **62**(1): p. 37-43.
459. Drews, S.J., et al., *SARS-CoV-2 Virus-Like Particle Neutralizing Capacity in Blood Donors Depends on Serological Profile and Donor-Declared SARS-CoV-2 Vaccination History.* Microbiol Spectr, 2022. **10**(1): p. e0226221.
460. Eldesoukey, N., et al., *SARS-CoV-2 antibody seroprevalence rates among Egyptian blood donors around the third wave: Cross-sectional study.* Health Sci Rep, 2022. **5**(3): p. e634.
461. Erikstrup, C., et al., *Seroprevalence and infection fatality rate of the SARS-CoV-2 Omicron variant in Denmark: A nationwide serosurveillance study.* Lancet Reg Health Eur, 2022. **21**: p. 100479.
462. Eskild, A., et al., *Prevalence of antibodies against SARS-CoV-2 among pregnant women in Norway during the period December 2019 through December 2020.* Epidemiol Infect, 2022. **150**: p. e28.
463. Fischer, B., C. Knabbe, and T. Vollmer, *SARS-CoV-2 IgG seroprevalence in blood donors located in three different federal states, Germany, March to June 2020.* Euro Surveill, 2020. **25**(28).
464. Fischer, B., T. Vollmer, and C. Knabbe, *SARS-CoV-2 IgG seroprevalence in blood donors located in three different federal states, Germany, July 2020 to June 2021 – a follow-up.* medRxiv, 2022((Fischer B., bfischer@hdz-nrw.de; Vollmer T.; Knabbe C.) Herz- und Diabeteszentrum NRW, Institut für Laboratoriums- und Transfusionsmedizin, Bad Oeynhausen, Germany).
465. Freeman, M.C., et al., *Immunocompromised Seroprevalence and Course of Illness of SARS-CoV-2 in One Pediatric Quaternary Care Center.* J Pediatric Infect Dis Soc, 2021. **10**(4): p. 426-431.
466. Furukawa, K., et al., *Seroepidemiological Survey of the Antibody for Severe Acute Respiratory Syndrome Coronavirus 2 with Neutralizing Activity at Hospitals: A Cross-sectional Study in Hyogo Prefecture, Japan.* JMA J, 2021. **4**(1): p. 41-49.
467. Galipeau, Y., et al., *Relative Ratios of Human Seasonal Coronavirus Antibodies Predict the Efficiency of Cross-Neutralization of SARS-CoV-2 Spike Binding to ACE2.* EBioMedicine, 2021. **74**: p. 103700.
468. Gallian, P., et al., *Lower prevalence of antibodies neutralizing SARS-CoV-2 in group O French blood donors.* Antiviral Res, 2020. **181**: p. 104880.
469. George, J.A., et al., *Sentinel seroprevalence of SARS-CoV-2 in Gauteng Province, South Africa, August - October 2020.* S Afr Med J, 2021. **111**(11): p. 1078-1083.

470. Germain, N., et al., *Retrospective study of COVID-19 seroprevalence among tissue donors at the onset of the outbreak before implementation of strict lockdown measures in France.* Cell Tissue Bank, 2021. **22**(3): p. 511-518.
471. Gidding, H.F., et al., *Seroprevalence of SARS-CoV-2-specific antibodies in Sydney after the first epidemic wave of 2020.* Med J Aust, 2021. **214**(4): p. 179-185.
472. Gragnani, L., et al., *SARS-CoV-2 was already circulating in Italy, in early December 2019.* Eur Rev Med Pharmacol Sci, 2021. **25**(8): p. 3342-3349.
473. Havers, F.P., et al., *Seroprevalence of Antibodies to SARS-CoV-2 in 10 Sites in the United States, March 23-May 12, 2020.* JAMA Intern Med, 2020. **180**(12): p. 1576-1576.
474. Herzog, S.A., et al., *Seroprevalence of IgG antibodies against SARS-CoV-2 - a serial prospective cross-sectional nationwide study of residual samples, Belgium, March to October 2020.* Euro Surveill, 2022. **27**(9): p. 2100419-2100419.
475. Hippich, M., et al., *A Public Health Antibody Screening Indicates a 6-Fold Higher SARS-CoV-2 Exposure Rate than Reported Cases in Children.* Med, 2021. **2**(2): p. 149-163 e4.
476. Ho, H.L., et al., *Seroprevalence of COVID-19 in Taiwan revealed by testing anti-SARS-CoV-2 serological antibodies on 14,765 hospital patients.* Lancet Reg Health West Pac, 2020. **3**: p. 100041.
477. Hobbs, C.V., et al., *Active Surveillance With Seroprevalence-based Infection Rates Indicates Racial Disparities With Pediatric SARS-CoV-2 Requiring Hospitalization in Mississippi, March 2020-February 2021.* Pediatr Infect Dis J, 2022. **41**(9): p. 736-741.
478. Honge, B.L., et al., *Long-term detection of SARS-CoV-2 antibodies after infection and risk of re-infection.* Int J Infect Dis, 2022. **116**: p. 289-292.
479. Hsieh, Y.H., et al., *A Tale of 3 Pandemics: Severe Acute Respiratory Syndrome Coronavirus 2, Hepatitis C Virus, and Human Immunodeficiency Virus in an Urban Emergency Department in Baltimore, Maryland.* Open Forum Infect Dis, 2022. **9**(5): p. ofac130.
480. Hughes, E.C., et al., *Severe Acute Respiratory Syndrome Coronavirus 2 Serosurveillance in a Patient Population Reveals Differences in Virus Exposure and Antibody-Mediated Immunity According to Host Demography and Healthcare Setting.* J Infect Dis, 2021. **223**(6): p. 971-980.
481. Jaiswal, R., et al., *Seroprevalence of SARS-CoV-2 IgG antibody among healthy blood donors: a single centre study.* Transfus Apher Sci, 2022. **61**(3): p. 103338.
482. Jeong, H.W., et al., *Differences in seroprevalence between epicenter and non-epicenter areas of the COVID-19 outbreak in South Korea.* J Microbiol, 2021. **59**(5): p. 530-533.
483. Jiang, C., et al., *Antibody seroconversion in asymptomatic and symptomatic patients infected with severe acute respiratory syndrome coronavirus 2 (SARS-CoV-2).* Clin Transl Immunology, 2020. **9**(9): p. e1182.
484. Jones, J.M., et al., *Estimated US Infection- and Vaccine-Induced SARS-CoV-2 Seroprevalence Based on Blood Donations, July 2020-May 2021.* JAMA, 2021. **326**(14): p. 1400-1409.
485. Kale, P., et al., *SARS-Coronavirus-2 seroprevalence in asymptomatic healthy blood donors: Indicator of community spread.* Transfus Apher Sci, 2022. **61**(1): p. 103293.
486. Kanji, J.N., et al., *Seropositivity to SARS-CoV-2 in Alberta, Canada in a post-vaccination period (March 2021-July 2021).* Infect Dis (Lond), 2022. **54**(9): p. 666-676.
487. Kaspersen, K.A., et al., *Estimation of SARS-CoV-2 Infection Fatality Rate by Age and Comorbidity Status Using Antibody Screening of Blood Donors During the COVID-19 Epidemic in Denmark.* J Infect Dis, 2022. **225**(2): p. 219-228.
488. Kennedy, J.L., et al., *Temporal Variations in Seroprevalence of Severe Acute Respiratory Syndrome Coronavirus 2 Infections by Race and Ethnicity in Arkansas.* Open Forum Infect Dis, 2022. **9**(5): p. ofac154.
489. Koureas, M., et al., *SARS-CoV-2 Sero-Surveillance in Greece: Evolution over Time and Epidemiological Attributes during the Pre-Vaccination Pandemic Era.* Diagnostics (Basel), 2022. **12**(2).

490. Kugeler, K.J., et al., *Assessment of SARS-CoV-2 Seroprevalence by Community Survey and Residual Specimens, Denver, Colorado, July-August 2020.* Public Health Rep, 2022. **137**(1): p. 128-136.
491. Laeyendecker, O., et al., *Demographic and clinical correlates of acute and convalescent SARS-CoV-2 infection among patients of a U.S. emergency department.* Am J Emerg Med, 2021. **48**: p. 261-268.
492. Le Vu, S., et al., *Prevalence of SARS-CoV-2 antibodies in France: results from nationwide serological surveillance.* Nat Commun, 2021. **12**(1): p. 3025.
493. Lerdsamran, H., et al., *Seroprevalence of anti-SARS-CoV-2 antibodies in Thai adults during the first three epidemic waves.* PLoS One, 2022. **17**(4): p. e0263316.
494. Lewin, A., et al., *SARS-CoV-2 seroprevalence among blood donors in Quebec, and analysis of symptoms associated with seropositivity: a nested case-control study.* Can J Public Health, 2021. **112**(4): p. 576-586.
495. Li, Z., et al., *Social Vulnerability and Rurality Associated With Higher Severe Acute Respiratory Syndrome Coronavirus 2 (SARS-CoV-2) Infection-Induced Seroprevalence: A Nationwide Blood Donor Study-United States, July 2020-June 2021.* Clin Infect Dis, 2022. **75**(1): p. e133-e143.
496. Lim, T., et al., *Changes in Severe Acute Respiratory Syndrome Coronavirus 2 Seroprevalence Over Time in 10 Sites in the United States, March-August, 2020.* Clin Infect Dis, 2021. **73**(10): p. 1831-1839.
497. Lombardi, F., et al., *Seroprevalence of SARS-CoV-2 Antibodies in HIV-Infected Patients in Rome, Italy during the COVID-19 Outbreak.* Diagnostics (Basel), 2021. **11**(7).
498. Lopez, C.A., et al., *Ethnoracial Disparities in SARS-CoV-2 Seroprevalence in a Large Cohort of Individuals in Central North Carolina from April to December 2020.* mSphere, 2022. **7**(3): p. e0084121.
499. Lopez, C.A., et al., *Disparities in SARS-CoV-2 seroprevalence among individuals presenting for care in central North Carolina over a six-month period.* medRxiv, 2021.
500. Lucinde, R., et al., *Sero-surveillance for IgG to SARS-CoV-2 at antenatal care clinics in three Kenyan referral hospitals: repeated cross-sectional surveys 2020-21.* medRxiv, 2022((Lucinde R., RLucinde@kemri-wellcome.org; Mugo D.; Karani A.; Gardiner E.; Gitonga J.; Karanja H.; Nyagwange J.; Tuju J.; Wanjiku P.; Nyutu G.; Etyang A.; Adetifa I.M.O.; Kagucia E.; Uyoga S.; Otiende M.; Otieno E.; Ndwiga L.; Agoti C.N.; Nyaguara A.; Bar).
501. Lynch, S.A., et al., *Prevalence of Neutralising Antibodies to HCoV-NL63 in Healthy Adults in Australia.* Viruses, 2021. **13**(8).
502. Machalek, D.A., et al., *Serological testing of blood donors to characterise the impact of COVID-19 in Melbourne, Australia, 2020.* PLoS One, 2022. **17**(7): p. e0265858.
503. Mahallawi, W. and N. Ibrahim, *Unexpected Detection of Anti-SARS-CoV-2 Antibodies Before the Declaration of the COVID-19 Pandemic.* Front Med (Lausanne), 2022. **9**((Mahallawi W., wmahallawi@gmail.com; Ibrahim N.) Medical Laboratory Technology Department, College of Applied Medical Sciences, Taibah University, Medina, Saudi Arabia): p. 923715.
504. Mahallawi, W.H. and A.H. Al-Zalabani, *The seroprevalence of SARS-CoV-2 IgG antibodies among asymptomatic blood donors in Saudi Arabia.* Saudi J Biol Sci, 2021. **28**(3): p. 1697-1701.
505. Mandolo, J., et al., *SARS-CoV-2 exposure in Malawian blood donors: an analysis of seroprevalence and variant dynamics between January 2020 and July 2021.* BMC Med, 2021. **19**(1): p. 303.
506. Maneikis, K., et al., *Immunogenicity of the BNT162b2 COVID-19 mRNA vaccine and early clinical outcomes in patients with haematological malignancies in Lithuania: a national prospective cohort study.* Lancet Haematol, 2021. **8**(8): p. e583-e592.
507. Manning, J., et al., *SARS-CoV-2 Cross-Reactivity in Prepandemic Serum from Rural Malaria-Infected Persons, Cambodia.* Emerg Infect Dis, 2022. **28**(2): p. 440-444.
508. Marchi, S., et al., *SARS-CoV-2 Circulation during the First Year of the Pandemic: A Seroprevalence Study from January to December 2020 in Tuscany, Italy.* Viruses, 2022. **14**(7).

509. Marsteller, N.L., et al., *Immune Response to SARS-CoV-2 in an Asymptomatic Pediatric Allergic Cohort.* Antibodies (Basel), 2021. **10**(2).
510. Martin, M.C., et al., *Persistence of SARS-CoV-2 total immunoglobulins in a series of convalescent plasma and blood donors.* PLoS One, 2022. **17**(2): p. e0264124.
511. Martinez-Acuna, N., et al., *Seroprevalence of Anti-SARS-CoV-2 Antibodies in Blood Donors from Nuevo Leon State, Mexico, during 2020: A Retrospective Cross-Sectional Evaluation.* Viruses, 2021. **13**(7).
512. McAuley, A., et al., *National population prevalence of antibodies to SARS-CoV-2 among pregnant women in Scotland during the second wave of the COVID-19 pandemic: a prospective national serosurvey.* Public Health, 2021. **199**: p. 17-19.
513. McCulloch, D.J., et al., *Seroprevalence of SARS-CoV-2 antibodies in Seattle, Washington: October 2019-April 2020.* PLoS One, 2021. **16**(5): p. e0252235.
514. Miyara, M., et al., *Pre-COVID-19 Immunity to Common Cold Human Coronaviruses Induces a Recall-Type IgG Response to SARS-CoV-2 Antigens Without Cross-Neutralisation.* Front Immunol, 2022. **13**((Miyara M.; Saichi M.; Sterlin D.; Mathian A.; Atif M.; Quentric P.; Mohr A.; Claër L.; Parizot C.; Dorgham K.; Yssel H.; Fadlallah J.; Chazal T.; Haroche J.; Amoura Z.; Gorochov G., guy.gorochov@sorbonne-universite.fr) Sorbonne Université, Inserm, Centre): p. 790334.
515. Moat, S.J., et al., *Maternal SARS-CoV-2 sero-surveillance using newborn dried blood spot (DBS) screening specimens highlights extent of low vaccine uptake in pregnant women.* Hum Vaccin Immunother, 2022. **18**(5): p. 2089498.
516. Moya Rios do Vale, N., et al., *Increasing rate of anti-SARS-CoV-2 antibodies between the first and second waves of COVID-19 in Sao Paulo, Brazil: A cross-sectional blood donors-based study.* Clinics (Sao Paulo), 2022. **77**: p. 100016.
517. Mun, K.H., et al., *Comparison of the Prevalence of Antibodies to SARS-CoV-2 in 9954 Recruits in the Korean Army Training Center with the General Korean Population of Equivalent Age Between September and November, 2020.* Med Sci Monit, 2022. **28**: p. e934926.
518. Munoz-Medina, J.E., et al., *SARS-CoV-2 IgG Antibodies Seroprevalence and Sera Neutralizing Activity in MEXICO: A National Cross-Sectional Study during 2020.* Microorganisms, 2021. **9**(4): p. 850-850.
519. Nah, E.H., et al., *Nationwide seroprevalence of antibodies to SARS-CoV-2 in asymptomatic population in South Korea: a cross-sectional study.* BMJ Open, 2021. **11**(4): p. e049837.
520. Ng, D.L., et al., *SARS-CoV-2 seroprevalence and neutralizing activity in donor and patient blood.* Nat Commun, 2020. **11**(1): p. 4698.
521. Nunhofer, V., et al., *Persistence of Naturally Acquired and Functional SARS-CoV-2 Antibodies in Blood Donors One Year after Infection.* Viruses, 2022. **14**(3).
522. Orner, E.P., et al., *Comparison of SARS-CoV-2 IgM and IgG seroconversion profiles among hospitalized patients in two US cities.* Diagn Microbiol Infect Dis, 2021. **99**(4): p. 115300.
523. Palmateer, N.E., et al., *National population prevalence of antibodies to SARS-CoV-2 in Scotland during the first and second waves of the COVID-19 pandemic.* Public Health, 2021. **198**: p. 102-105.
524. Park, S., et al., *Serological evaluation of patients with coronavirus disease-2019 in Daegu, South Korea.* PLoS One, 2022. **17**(1): p. e0262820.
525. Pedersen, J., et al., *Cross-reactive immunity against SARS-CoV-2 N protein in Central and West Africa precedes the COVID-19 pandemic.* Sci Rep, 2022. **12**(1): p. 12962.
526. Piron, M., et al., *SARS-CoV-2 seroprevalence in blood donors before and after the first wave in Catalonia (Spain).* Blood Transfus, 2022. **20**(5): p. 353-361.
527. Prete, C.A., Jr., et al., *Reinfection by the SARS-CoV-2 Gamma variant in blood donors in Manaus, Brazil.* BMC Infect Dis, 2022. **22**(1): p. 127.
528. Raouf, M., et al., *Seroprevalence of IgG Anti-SARS-CoV-2 among Voluntary Blood Donors in Dubai: Demographic and Risk Factors.* Dubai Medical Journal, 2021. **4**(3): p. 204-211.

529. Rapsinski, G.J., et al., *Pediatric SARS-CoV-2 seroprevalence during mitigation procedures in Southwestern Pennsylvania.* J Clin Virol Plus, 2021. **1**(3): p. 100026.
530. Reedman, C.N., et al., *Changing Patterns of SARS-CoV-2 Seroprevalence among Canadian Blood Donors during the Vaccine Era.* Microbiol Spectr, 2022. **10**(2): p. e0033922.
531. Robertson, L.J., et al., *Evaluation of the IgG antibody response to SARS CoV-2 infection and performance of a lateral flow immunoassay: cross-sectional and longitudinal analysis over 11 months.* BMJ Open, 2021. **11**(6): p. e048142.
532. Rostad, C.A., et al., *Quantitative SARS-CoV-2 Serology in Children With Multisystem Inflammatory Syndrome (MIS-C).* Pediatrics, 2020. **146**(6).
533. Rotee, I.L.M., et al., *Trends in SARS-CoV-2 seroprevalence amongst urban paediatric patients compared with a nationwide cohort in the Netherlands.* J Clin Virol Plus, 2021. **1**(4): p. 100045.
534. Routledge, I., et al., *Using sero-epidemiology to monitor disparities in vaccination and infection with SARS-CoV-2.* Nat Commun, 2022. **13**(1): p. 2451.
535. Saeed, S., et al., *SARS-CoV-2 seroprevalence among blood donors after the first COVID-19 wave in Canada.* Transfusion, 2021. **61**(3): p. 862-872.
536. Saeed, S., et al., *Severe acute respiratory syndrome coronavirus 2 (SARS-CoV-2) seroprevalence: Navigating the absence of a gold standard.* PLoS One, 2021. **16**(9): p. e0257743.
537. Sam, I.C., et al., *Changing predominant SARS-CoV-2 lineages drives successive COVID-19 waves in Malaysia, February 2020 to March 2021.* J Med Virol, 2022. **94**(3): p. 1146-1153.
538. Sanada, T., et al., *Serologic Survey of IgG Against SARS-CoV-2 Among Hospital Visitors Without a History of SARS-CoV-2 Infection in Tokyo, 2020-2021.* J Epidemiol, 2022. **32**(2): p. 105-111.
539. Schoenhals, M., et al., *SARS-CoV-2 antibody seroprevalence follow-up in Malagasy blood donors during the 2020 COVID-19 Epidemic.* EBioMedicine, 2021. **68**: p. 103419.
540. Sebastiao, C.S., et al., *Seroprevalence of anti-SARS-CoV-2 antibodies and risk factors among healthy blood donors in Luanda, Angola.* BMC Infect Dis, 2021. **21**(1): p. 1131.
541. Sharifi, Z., M. Zadsar, and S. Samiei, *A Pilot Study on Presence of SARS-CoV-2-RNA in Iranian Blood Donors.* Jundishapur Journal of Microbiology, 2021. **14**(6).
542. Sheward, D.J., et al., *Neutralisation sensitivity of the SARS-CoV-2 omicron (B.1.1.529) variant: a cross-sectional study.* Lancet Infect Dis, 2022. **22**(6): p. 813-820.
543. Simani, O.E., et al., *Low seroprevalence of hepatitis E virus in pregnant women in an urban area near Pretoria, South Africa.* IJID Reg, 2022. **2**: p. 70-73.
544. Soeorg, H., et al., *Seroprevalence and levels of IgG antibodies after COVID-19 infection or vaccination.* Infect Dis (Lond), 2022. **54**(1): p. 63-71.
545. Spinelli, M.A., et al., *SARS-CoV-2 seroprevalence, and IgG concentration and pseudovirus neutralising antibody titres after infection, compared by HIV status: a matched case-control observational study.* Lancet HIV, 2021. **8**(6): p. e334-e341.
546. Ssuuna, C., et al., *Severe Acute Respiratory Syndrome Coronavirus-2 seroprevalence in South-Central Uganda, during 2019-2021.* BMC Infect Dis, 2022. **22**(1): p. 174.
547. Stadlbauer, D., et al., *Repeated cross-sectional sero-monitoring of SARS-CoV-2 in New York City.* Nature, 2021. **590**(7844): p. 146-150.
548. Stone, M., et al., *Use of US Blood Donors for National Serosurveillance of Severe Acute Respiratory Syndrome Coronavirus 2 Antibodies: Basis for an Expanded National Donor Serosurveillance Program.* Clin Infect Dis, 2022. **74**(5): p. 871-881.
549. Suda, G., et al., *Time-dependent changes in the seroprevalence of COVID-19 in asymptomatic liver disease outpatients in an area in Japan undergoing a second wave of COVID-19.* Hepatol Res, 2020. **50**(10): p. 1196-1200.
550. Sughayer, M.A., et al., *Dramatic rise in seroprevalence rates of SARS-CoV-2 antibodies among healthy blood donors: The evolution of a pandemic.* Int J Infect Dis, 2021. **107**: p. 116-120.
551. Sutton, M., P. Cieslak, and M. Linder, *Notes from the Field: Seroprevalence Estimates of SARS-CoV-2 Infection in Convenience Sample - Oregon, May 11-June 15, 2020.* MMWR Morb Mortal Wkly Rep, 2020. **69**(32): p. 1100-1101.

552. Sykes, W., et al., *Prevalence of anti-SARS-CoV-2 antibodies among blood donors in Northern Cape, KwaZulu-Natal, Eastern Cape, and Free State provinces of South Africa in January 2021.* Res Sq, 2021.
553. Takayama, Y., et al., *Pre-Vaccination Anti-Severe Acute Respiratory Syndrome Coronavirus 2 Antibody Seroprevalence in Workers at Three Japanese Hospitals.* J Nippon Med Sch, 2022. **89**(5): p. 513-519.
554. Tanunliong, G., et al., *Age-Associated Seroprevalence of Coronavirus Antibodies: Population-Based Serosurveys in 2013 and 2020, British Columbia, Canada.* Front Immunol, 2022. **13**: p. 836449.
555. Tea, F., et al., *SARS-CoV-2 neutralizing antibodies: Longevity, breadth, and evasion by emerging viral variants.* PLoS Med, 2021. **18**(7): p. e1003656.
556. Thompson, C.P., et al., *Detection of neutralising antibodies to SARS-CoV-2 to determine population exposure in Scottish blood donors between March and May 2020.* Euro Surveill, 2020. **25**(42).
557. Tso, F.Y., et al., *High prevalence of pre-existing serological cross-reactivity against severe acute respiratory syndrome coronavirus-2 (SARS-CoV-2) in sub-Saharan Africa.* Int J Infect Dis, 2021. **102**: p. 577-583.
558. Tuite, A.R., et al., *Estimating SARS-CoV-2 Seroprevalence in Canadian Blood Donors, April 2020 to March 2021: Improving Accuracy with Multiple Assays.* Microbiol Spectr, 2022. **10**(1): p. e0256321.
559. Tunheim, G., et al., *Prevalence of antibodies against SARS-CoV-2 in the Norwegian population, August 2021.* Influenza Other Respir Viruses, 2022. **16**(6): p. 1004-1013.
560. Tunheim, G., et al., *Trends in seroprevalence of SARS-CoV-2 and infection fatality rate in the Norwegian population through the first year of the COVID-19 pandemic.* Influenza Other Respir Viruses, 2022. **16**(2): p. 204-212.
561. Uyoga, S., et al., *Seroprevalence of anti-SARS-CoV-2 IgG antibodies in Kenyan blood donors.* Science, 2021. **371**(6524): p. 79-82.
562. Valenti, L., et al., *SARS-CoV-2 seroprevalence trends in healthy blood donors during the COVID-19 outbreak in Milan.* Blood Transfus, 2021. **19**(3): p. 181-189.
563. van den Hurk, K., et al., *Low awareness of past SARS-CoV-2 infection in healthy plasma donors.* Cell Rep Med, 2021. **2**(3): p. 100222.
564. Vanroye, F., et al., *COVID-19 Antibody Detecting Rapid Diagnostic Tests Show High Cross-Reactivity When Challenged with Pre-Pandemic Malaria, Schistosomiasis and Dengue Samples.* Diagnostics (Basel), 2021. **11**(7).
565. Vassallo, R.R., et al., *Progression and Predictors of SARS-CoV-2 Antibody Seroreactivity In US Blood Donors.* Transfus Med Rev, 2021. **35**(3): p. 8-15.
566. Vette, K.M., et al., *Seroprevalence of Severe Acute Respiratory Syndrome Coronavirus 2-Specific Antibodies in Australia After the First Epidemic Wave in 2020: A National Survey.* Open Forum Infect Dis, 2022. **9**(3): p. ofac002.
567. Wachter, F., et al., *Continuous monitoring of SARS-CoV-2 seroprevalence in children using residual blood samples from routine clinical chemistry.* Clin Chem Lab Med, 2022. **60**(6): p. 941-951.
568. Walker, A.G., et al., *SARS-CoV-2 Antibody Seroprevalence Among Maintenance Dialysis Patients in the United States.* Kidney Med, 2021. **3**(2): p. 216-222 e1.
569. Weidner, L., et al., *Seroprevalence of anti-SARS-CoV-2 total antibody is higher in younger Austrian blood donors.* Infection, 2021. **49**(6): p. 1187-1194.
570. Whitaker, H.J., et al., *Pfizer-BioNTech and Oxford AstraZeneca COVID-19 vaccine effectiveness and immune response amongst individuals in clinical risk groups.* J Infect, 2022. **84**(5): p. 675-683.
571. Xiao, C., et al., *Seroprevalence of Antibodies to SARS-CoV-2 in Guangdong Province, China between March to June 2020.* Pathogens, 2021. **10**(11).

572. Younas, A., et al., *Seroprevalence of SARS-CoV-2 antibodies among healthy blood donors in Karachi, Pakistan.* Transfus Apher Sci, 2020. **59**(6): p. 102923.
573. Kriz, B., et al., *Results of the Screening of Tick-Borne Encephalitis Virus Antibodies in Human Sera from Eight Districts Collected Two Decades Apart.* Vector Borne Zoonotic Dis, 2015. **15**(8): p. 489-93.
574. Stefanoff, P., et al., *Identification of new endemic tick-borne encephalitis foci in Poland – a pilot seroprevalence study in selected regions.* International Journal of Medical Microbiology, 2008. **298**(SUPPL. 1): p. 102-107.
575. Hefele, L., et al., *An age-stratified serosurvey against purified Salmonella enterica serovar Typhi antigens in the Lao People s Democratic Republic.* PLoS Negl Trop Dis, 2021. **15**(12): p. e0010017.
576. Aebi, C., et al., *Age-specific seroprevalence to varicella-zoster virus: study in Swiss children and analysis of European data.* Vaccine, 2001. **19**(23-24): p. 3097-103.
577. Coghill, A.E., et al., *Prospective investigation of herpesvirus infection and risk of glioma.* Int J Cancer, 2022. **151**(2): p. 222-228.
578. Davidovici, B.B., et al., *Comparison of the dynamics and correlates of transmission of Herpes Simplex Virus-1 (HSV-1) and Varicella-Zoster Virus (VZV) in a sample of the Israeli population.* Eur J Epidemiol, 2007. **22**(9): p. 641-6.
579. Fatha, N., L.W. Ang, and K.T. Goh, *Changing seroprevalence of varicella zoster virus infection in a tropical city state, Singapore.* Int J Infect Dis, 2014. **22**: p. 73-7.
580. Gabutti, G., et al., *The epidemiology of Varicella Zoster Virus infection in Italy.* BMC Public Health, 2008. **8**(1): p. 372.
581. Han, S.B., et al., *Seroepidemiology of varicella-zoster virus in Korean adolescents and adults using fluorescent antibody to membrane antigen test.* Epidemiol Infect, 2015. **143**(8): p. 1643-50.
582. Izquierdo, G., et al., *[Seroprevalence of varicella-zoster virus in children with cancer in six hospitals in Santiago, Chile].* Rev Chilena Infectol, 2012. **29**(6): p. 595-9.
583. Jons, D., et al., *Follow-up after infectious mononucleosis in search of serological similarities with presymptomatic multiple sclerosis.* Mult Scler Relat Disord, 2021. **56**: p. 103288.
584. Kohlmann, R., et al., *Serological evidence of increased susceptibility to varicella-zoster virus reactivation or reinfection in natalizumab-treated patients with multiple sclerosis.* Mult Scler, 2015. **21**(14): p. 1823-32.
585. Kudesia, G., et al., *Changes in age related seroprevalence of antibody to varicella zoster virus: impact on vaccine strategy.* J Clin Pathol, 2002. **55**(2): p. 154-5.
586. Lee, H., H.K. Cho, and K.H. Kim, *Seroepidemiology of varicella-zoster virus in Korea.* J Korean Med Sci, 2013. **28**(2): p. 195-9.
587. Medic, S., et al., *Seroepidemiology of varicella zoster virus infection in Vojvodina, Serbia.* Epidemiol Infect, 2018. **146**(12): p. 1593-1601.
588. Nardone, A., et al., *The comparative sero-epidemiology of varicella zoster virus in 11 countries in the European region.* Vaccine, 2007. **25**(45): p. 7866-72.
589. Purswani, M.U., et al., *Prevalence and Persistence of Varicella Antibodies in Previously Immunized Children and Youth With Perinatal HIV-1 Infection.* Clin Infect Dis, 2016. **62**(1): p. 106-114.
590. Ratnam, S., *Varicella susceptibility in a Canadian population.* Can J Infect Dis, 2000. **11**(5): p. 249-53.
591. Reis, A.D., C.S. Pannuti, and V.A. de Souza, *[Prevalence of varicella-zoster virus antibodies in young adults from different Brazilian climatic regions].* Rev Soc Bras Med Trop, 2003. **36**(3): p. 317-20.
592. Rimseliene, G., et al., *Varicella-zoster virus susceptibility and primary healthcare consultations in Norway.* BMC Infect Dis, 2016. **16**(1): p. 254.
593. Saha, S.K., et al., *Seroepidemiology of varicella-zoster virus in Bangladesh.* Ann Trop Paediatr, 2002. **22**(4): p. 341-5.

594. Siennicka, J., et al., *Seroprevalence of varicella-zoster virus in Polish population.* Przegl Epidemiol, 2009. **63**(4): p. 495-9.
595. Thiry, N., et al., *The seroepidemiology of primary varicella-zoster virus infection in Flanders (Belgium).* Eur J Pediatr, 2002. **161**(11): p. 588-93.
596. Tseng, H.F., et al., *A seroepidemiology study of varicella among children aged 0-12 years in Taiwan.* The Southeast Asian journal of tropical medicine and public health, 2005. **36**(5): p. 1201-7.
597. Vyse, A.J., et al., *Seroprevalence of antibody to varicella zoster virus in England and Wales in children and young adults.* Epidemiol Infect, 2004. **132**(6): p. 1129-34.
598. Widgren, K., et al., *Severe chickenpox disease and seroprevalence in Sweden - implications for general vaccination.* Int J Infect Dis, 2021. **111**: p. 92-98.
599. Wiese-Posselt, M., et al., *Varicella-zoster virus seroprevalence in children and adolescents in the pre-varicella vaccine era, Germany*. BMC Infect Dis, 2017. **17**(1): p. 356.
600. Pistone, T., et al., *Immunogenicity and tolerability of yellow fever vaccination in 23 French HIV-infected patients.* Curr HIV Res, 2010. **8**(6): p. 461-6.
601. Veit, O., et al., *Long-term Immune Response to Yellow Fever Vaccination in Human Immunodeficiency Virus (HIV)-Infected Individuals Depends on HIV RNA Suppression Status: Implications for Vaccination Schedule.* Clin Infect Dis, 2018. **66**(7): p. 1099-1108.

Table S5. Paper objective excluding articles describing population seropositivity (N = 117 studies). Note the categories below are not mutually exclusive meaning that one study could have conducted testing for multiple pathogens and have had multiple objectives.

**Objective**	**Total, N (column %)**
Describe seroprevalence among a clinical subpopulation	69 (59.0)
Identify risk factors of seropositivity	38 (32.5)
Evaluate outcomes of seropositivity	19 (16.2)
Evaluate the need for blood donor screening	16 (13.7)
Estimate parameters related to the magnitude or timing of infection or understand transmission dynamics	16 (13.7)
Describe antibody kinetics following infection	12 (10.3)
Describe antibody kinetics following vaccination	11 (9.4)
Evaluate changes in seropositivity over time	10 (8.5)
Evaluate antibody cross-reactivity	10 (8.5)
Compare population seropositivity between countries or international regions	3 (2.6)
Describe viral dynamics	2 (1.7)
Compare the use of residual samples to population-based samples	1 (0.9)
Total	117 (100)

Table S6. Collection site and Storage sites of the sample (N = 601 studies). Note, the categories below are not mutually exclusive meaning that one article could have multiple collection or storage sites.

	**No. of papers (%)**
**Collection Site of Specimens**	
Blood and plasma donation site	208 (34.6)
Hospital/ Clinic	207 (34.4)
Diagnostic center/ Laboratory	163 (27.1)
Household	33 (5.5)
Unknown	79 (13.1)
Other [a]	12 (2.0)
**Storage Site**	
Diagnostic center/laboratory	235 (39.1)
Research biorepository	199 (33.1)
Blood/plasma donation center	192 (32.0)
Unspecified biorepository	30 (5.0)

a. Other collection sites included homeless shelters, schools and prisons.

Table S7. Number of articles utilizing residual specimens from serological surveillance systems

Identified Serological Surveillance System	Number of articles
Health Protection Agency serological surveillance programme (England)	12
European Sero-Epidemiology Network (ESEN)	11
National Centre for Immunisation Research and Surveillance (NCIRS) (Australia)	4
Vietnam serosurveillance (serum biobank project)	1

Table S8. Meta-data linked to residual samples by the original use of the sample (N = 601 studies). Note, the categories below are not mutually exclusive meaning that one article could have residual samples linked to multiple categories of meta-data.

Meta-data linked to residual samples	Total n (column %)	Original use of specimen n (column %)			
		Blood or plasma donation	Diagnostic specimens	Serosurvey	Other non-serological survey
Basic demographic data (i.e., age, sex, race, ethnicity)	567 (94.3)	194 (88.2)	356 (96.0)	62 (95.4)	32 (100)
Extended demographic data (e.g., residence, occupation, SES)	284 (47.3)	102 (46.4)	158 (42.6)	36 (55.4)	15 (46.9)
Epidemiologic data on VPD (e.g., vaccination status or infection history)	106 (17.6)	25 (11.4)	66 (17.8)	21 (32.3)	9 (28.1)
Epidemiologic data not on VPD (e.g., vaccination status or infection history)	97 (16.1)	33 (15)	62 (16.7)	11 (16.9)	7 (21.9)
None	25 (4.2)	19 (8.6)	9 (2.4)	3 (4.6)	0 (0)
Total	601 (100)	220 (100)	371 (100)	65 (100)	32 (100)

Table S9. Permissions and ethical considerations by original use of specimens (N=601 articles). Note, the categories below are not mutually exclusive meaning that one article could have discussed multiple permission or ethical considerations.

**Permission and Ethical Considerations**	**Total n (column %)**	**Original use of specimen n (column %)**			
		**Blood or plasma donation**	**Diagnostic specimens**	**Serosurvey**	**Other non-serological survey**
Approved by ethics board/committee	423 (70.4)	149 (67.7)	257 (69.3)	43 (66.2)	29 (90.6)
Broad consent at time of specimen collection	199 (33.1)	91 (41.4)	94 (25.3)	27 (41.5)	16 (50.0)
Samples de-identified	199 (33.1)	60 (27.3)	137 (36.9)	13 (20.0)	10 (31.3)
No statement about permissions or ethics	88 (14.6)	37 (16.8)	58 (15.6)	14 (21.5)	2 (6.3)
Waiver on re-consenting	45 (7.5)	14 (6.4)	29 (7.8)	2 (3.1)	0 (0)
Exempt as public health surveillance	20 (3.3)	4 (1.8)	15 (4.0)	2 (3.1)	0 (0)
Other	8 (1.3)	3 (1.4)	4 (1.1)	0 (0)	0 (0)
Reconsent given	2 (0.3)	0 (0)	1 (0.3)	1 (1.5)	0 (0)
Total	601 (100)	220 (100)	371 (100)	65 (100)	32 (100)

Table S10. Permissions and ethical considerations for articles mentioning residual specimens were de-identified (N=199 articles). Note, the categories below are not mutually exclusive meaning that one article could have discussed multiple permission or ethical considerations.

**Permission and Ethical Considerations**	**Total n (column %)**
Approved by ethics board/committee	144 (72.4)
Broad consent at time of specimen collection	54 (27.1)
Samples de-identified	199 (100)
No statement about permissions or ethics	
Waiver on re-consenting	28 (14.1)
Exempt as public health surveillance	8 (4.0)
Other	3 (1.5)
Reconsent given	0 (0.0)
Total	199 (100)
